# Postpartum care utilization among high-risk pregnancies in an urban safety-net health system

**DOI:** 10.1080/14767058.2026.2663199

**Published:** 2026-05-10

**Authors:** Kavita Vani, Rachel Nordlicht, Jessica Atrio, Kevin Fiori, David Lounsbury

**Affiliations:** a Department of Obstetrics & Gynecology and Women’s Health, Albert Einstein College of Medicine and Montefiore Medical Center, Bronx, NY, USA; b Department of Pediatrics, Division of Community and Population Health, Albert Einstein College of Medicine, Bronx, NY, USA; c Albert Einstein College of Medicine, Bronx, NY, USA; d Department of Family & Social Medicine, Albert Einstein College of Medicine, Bronx, NY, USA; e Department of Epidemiology and Population Health, Albert Einstein College of Medicine, Bronx, NY, USA

**Keywords:** Postpartum care, high-risk pregnancy, health services utilization, hypertensive disorders of pregnancy, gestational diabetes

## Abstract

**Introduction:**

The postpartum period carries substantial risk for preventable morbidity, particularly among individuals with high-risk pregnancies complicated by chronic or pregnancy-associated conditions. Despite recommendations for early and ongoing postpartum follow-up, patterns of postpartum and acute care utilization across high-risk conditions remain poorly characterized. We evaluated postpartum and acute care utilization among individuals with high-risk pregnancies versus low-risk pregnancies in an urban safety-net health system.

**Methods:**

We conducted a retrospective cohort study of individuals who delivered at two campuses of a tertiary academic medical center between 1 June 2018 and 31 May 2022. High-risk pregnancy status was defined using ICD-9/10 codes corresponding to chronic medical or pregnancy-associated conditions; low-risk was defined by the absence of these codes. The primary outcome was attendance of ≥1 postpartum visit (PPV) within 12 weeks of delivery. Secondary outcomes included emergency department (ED) visits and hospital readmissions within 12 weeks postpartum. Multivariable logistic regression was used to estimate adjusted odds ratios (aORs).

**Results:**

Of 13,874 included individuals, 9435 (68.0%) had ≥1 high-risk diagnosis, and nearly half had multiple coexisting conditions. High-risk individuals were more likely than low-risk individuals to attend any PPV (59.2% vs. 45.4%) and an early PPV within 21 days (40.5% vs. 24.2%, both *p* < 0.001). ED visits (16.9% vs. 13.9%) and readmissions (5.4% vs. 2.7%) were also more frequent among high-risk individuals (*p* < 0.001). In adjusted analyses, hypertensive disorders of pregnancy (aOR 1.67), mental health conditions (aOR 1.49), cesarean delivery, and greater prenatal care utilization were associated with higher odds of PPV attendance, while gestational diabetes was associated with lower odds (aOR 0.79). High-risk conditions, particularly hypertensive disorders and pregestational diabetes, were associated with increased acute care utilization.

**Conclusions:**

Although high-risk individuals were more likely to attend PPVs, overall engagement remained suboptimal and acute care utilization was high. These findings highlight the need for risk-tailored postpartum care and improved care coordination to reduce preventable morbidity.

## Introduction

The postpartum period is a critical window to address preventable complications and support long-term maternal health [1]. Individuals with high-risk pregnancies, defined as pregnancies complicated by preexisting chronic conditions or pregnancy-associated conditions, such as hypertensive disorders of pregnancy (HDP) or gestational diabetes, are at particularly high risk for postpartum morbidity and mortality and long-term cardiovascular risk [2,3]. Despite clear guidance from the American College of Obstetricians and Gynecologists (ACOG) recommending early and ongoing follow-up, postpartum care is often delayed, fragmented, or missed entirely [4–7], particularly among individuals facing social and structural barriers to care [8–10].

In the United States, more than half of pregnancy-related deaths occur during the postpartum period, and over 80% are considered preventable [11,12]. While the maternal mortality rate in the United States is unacceptably high, exceeding that of other high-income countries by a factor of 2–10 [12,13], the broader burden lies in pregnancy and postpartum morbidity and its long-term sequelae [14–16]. Pregnancy also represents a key opportunity to diagnose and initiate management of chronic conditions, making effective transition to postpartum care essential for long-term health [1,5]. However, postpartum care utilization among high-risk individuals remains incompletely characterized, particularly with respect to how patterns of scheduled and acute care vary across specific conditions.

The Bronx community served by our institution experiences a disproportionate burden of chronic medical and mental health conditions alongside high rates of socioeconomic disadvantage [17]. Pregnancy-associated mortality is high in the Bronx, where rates are several-fold higher than the national level [12,18]. Moreover, in the Bronx, for every maternal death, as many as 100 women experience severe maternal morbidity [14].

In this study, we sought to (1) characterize the distribution of high-risk conditions among pregnancies, (2) compare postpartum care utilization, including scheduled postpartum visits (PPVs), emergency department (ED) visits, and hospital readmissions, between high-risk and low-risk pregnancies, and (3) assess how these patterns differ across specific high-risk conditions. This analysis aims to establish a foundation for developing models that better support postpartum engagement and long-term health.

## Methods

We conducted a retrospective cohort study of all individuals who delivered at two campuses within our urban, academic, tertiary care medical center between 1 June 2018 and 31 May 2022. Data were extracted from the electronic medical record (EMR). Variables included maternal age, race, ethnicity, parity, insurance status, gestational age at initial prenatal visit and delivery, number of prenatal visits, mode of delivery, multifetal gestation, admission to the neonatal intensive care unit (NICU), and infant length of stay. Deliveries were identified using standardized delivery encounter codes recorded in the EMR. To ensure one observation per individual, only the first delivery of the study period was included.

The primary exposure was high-risk pregnancy status. High-risk status was determined based on the presence of at least one of 2013 ICD-9/10 maternal or fetal diagnosis codes (Appendix 1) corresponding to pregnancy-related or chronic medical conditions associated with increased morbidity in a patient’s prenatal or delivery record. The code list was compiled and manually reviewed based on high-risk conditions from published literature demonstrating their association with maternal morbidity and mortality [3,19]. Conditions were grouped into clinically relevant categories, including HDP, obesity, gestational and pregestational diabetes, and mental health conditions, and other medical comorbidities (Supplemental Table S1). Postpartum hemorrhage and stillbirth were also included as high-risk categories. Stillbirth was defined using ICD codes or Apgar scores of 0 at 1 and 5 min. Low-risk pregnancies were determined based on the absence of high-risk diagnosis code associated with chronic or pregnancy-associated conditions in a patient’s prenatal or delivery record.

Individuals were included if they delivered during the study period and received prenatal care at our institution (>1 documented obstetric or family medicine prenatal visit). Those receiving prenatal care elsewhere, seen only for consultation or delivering prior to 24 weeks were excluded. Records with incomplete or implausible EMR data were excluded following data quality review.

The primary outcome was the attendance of at least one PPV within the first 12 weeks after delivery at an affiliated obstetric or family medicine practice. During the COVID-19 period, PPVs were delivered via both in-person and telehealth modalities; however, visit type could not be distinguished in the available data. Additional postpartum care utilization measures included early PPV attendance within 21 days of delivery and total number of PPVs within 12 weeks. Secondary outcomes were ED visits or hospital readmissions within the first 12 weeks postpartum.

Patient characteristics and high-risk status were summarized using descriptive statistics. Categorical variables were compared using chi-square or Fisher’s exact tests, and continuous variables using *t*-tests and Wilcoxon’s rank-sum tests as appropriate. Multivariable logistic regression models estimated adjusted odds ratios (aORs) and 95% confidence intervals (CIs) for associations between high-risk status and PPV attendance. Co-variates were selected *a priori* based on clinical relevance and prior literature, including maternal age, race, ethnicity, insurance status, parity, preterm birth, mode of delivery, stillbirth, multifetal gestation, NICU admission, infant length of stay, number of prenatal visits, gestational age at initial prenatal visit, and delivery timing in relation to the COVID-19 pandemic. COVID-19 period definitions are provided in Supplemental methods. Secondary models evaluated acute care utilization with additional adjustment for PPV attendance. Records with missing exposure, outcome, or covariate data were excluded (<5% for all variables). Analyses were performed in Stata/BE 17 (StataCorp, College Station, TX). This study was performed in accordance with the principles stated in the Declaration of Helsinki. The study was approved by the Albert Einstein College of Medicine Review Board (IRB # 2022–14176 on 22 December 2022) with a waiver of informed consent.

## Results

### Cohort characteristics

**Figure 1.**
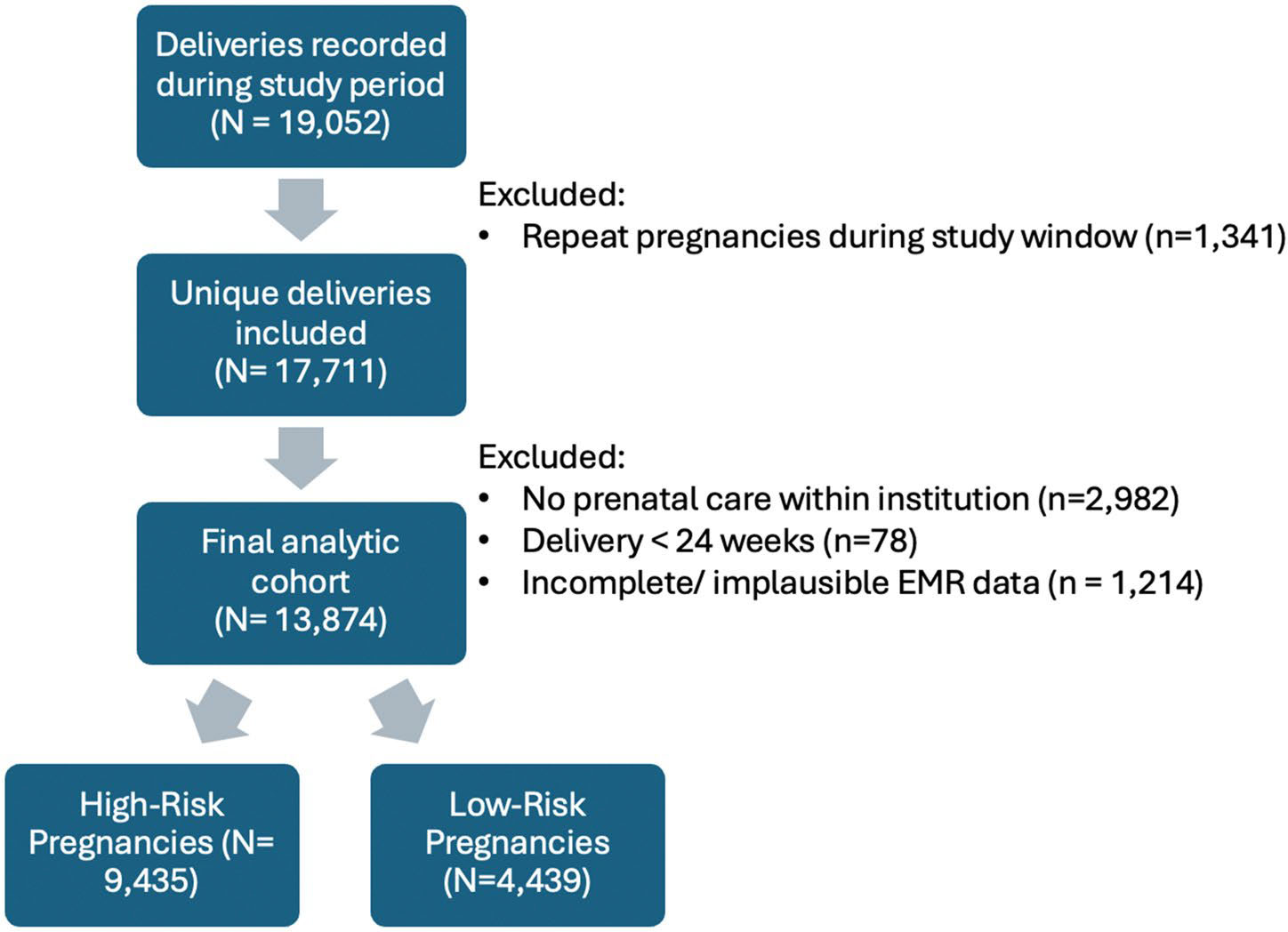
Flow diagram cohort derivation. Deliveries were identified using standardized delivery encounter codes. Exclusions were applied sequentially for repeat deliveries, lack of prenatal care within the institution, delivery before 24 weeks’ gestation, and incomplete or implausible records. The final analytic cohort included 13,874 unique deliveries, of which 9435 were classified as high-risk and 4439 as low-risk.

Among the 17,711 deliveries that occurred during the study period, 13,874 (78.3%) individuals met inclusion criteria (Figure 1). Of these, 9435 (68.0%) had at least one high-risk diagnosis. Compared with low-risk pregnancies, high-risk pregnancies were older, more likely to identify as Black, and more likely to deliver via cesarean (Table 1). High-risk pregnancies also had higher rates of preterm birth, admissions to the NICU, and greater prenatal care utilization.

Obesity (43.9%) and HDP (37.9%) were the most common high-risk conditions (Table 2). Nearly, half of high-risk individuals (45.9%) had two or more high-risk conditions, most commonly involving combinations of obesity, HDP, and gestational or pregestational diabetes.

### Postpartum follow-up and acute care utilization by risk status

Postpartum follow-up and acute care utilization differed significantly between the high-risk and low-risk pregnancies (Table 3). High-risk individuals were more likely than low-risk individuals to attend any PPV within 12 weeks of delivery (59.2% vs. 45.4%) and were more likely to attend an early PPV within 21 days of delivery (40.5% vs. 24.2%). High-risk individuals also attended a greater number of PPVs and were more likely to attend two or more PPVs (33.8% vs. 21.2%). ED visits (16.9% vs. 13.9%) and readmissions (5.4% vs. 2.7%) were also more frequent among high-risk individuals (all *p* < 0.001).

### Postpartum follow-up and acute care utilization by high-risk condition

To examine patterns of postpartum care utilization across specific high-risk conditions, subgroup analyses compared outcomes among individuals with each diagnosis relative to those without that diagnosis (Table 4). PPV attendance and acute care utilization varied across high-risk diagnostic categories. HDP was associated with the highest postpartum follow-up rates, with 66.1% attending at least one visit within 12 weeks. Individuals with obesity, gestational diabetes (GDM), mental health conditions and pregestational diabetes had higher PPV attendance compared with those without the respective diagnosis.

Acute care utilization also differed by condition. ED visits and readmissions were highest among individuals with HDP (18.4% and 6.9%) and pregestational diabetes (24.3% and 8.8%). ED visits were significantly more frequent among individuals with HDP, obesity, mental health conditions, and pregestational diabetes relative to those without these diagnoses. Across conditions, HDP and pregestational diabetes were associated with the greatest postpartum health care utilization, whereas GDM demonstrated more modest differences.

### Multivariable predictors of postpartum care and acute utilization

In adjusted analyses evaluating predictors of postpartum follow-up, HDP (aOR 1.67, 95% CI 1.53–1.83), mental health conditions (1.49, 95% CI 1.30–1.70), cesarean delivery (1.84, 95% CI 1.70–2.00), and greater prenatal care utilization (aOR 1.12, 95% CI 1.11–1.13) were independently associated with increased odds of PPV attendance (Table 5). These adjusted findings were consistent with the higher postpartum follow-up observed among individuals with these conditions in descriptive analyses. Delivery during COVID-19 pandemic periods were also associated with higher postpartum follow-up. Conversely, GDM (aOR 0.79, 95% CI 0.70–0.89) and multifetal gestation (aOR 0.28, 95% CI 0.20–0.40 for twin gestations and aOR 0.03, 95% CI 0.00–0.23 for triplet gestations) were independently associated with lower odds of postpartum follow-up.

Predictors of ED visits included attendance of a PPV within 12 weeks of delivery (aOR 1.42, 95% CI 1.28–1.57), HDP (aOR 1.15, 95% CI 0.98–1.21), pregestational diabetes (aOR 1.33, 95% CI 1.04–1.41), mental health conditions (aOR 1.31, 95% CI 1.03–1.73), cesarean delivery (aOR 1.30, 95% CI 1.18–1.44), Medicaid insurance (aOR 1.5, 95% CI 1.32–1.71), and delivery during peak (aOR 0.56, 95% CI 0.45–0.69) or late (aOR 0.83, 95% CI 0.73–0.94) COVID-19 pandemic.

Predictors of hospital readmission are shown in Supplemental Table S2. PPV attendance (aOR 2.56, 95% CI 2.08–3.16) and HDP (aOR 1.50, 95% CI 1.25–1.80) were the strongest predictors of readmission within 12 weeks postpartum. Black and Asian race, older maternal age, and primiparity were also associated with increased odds of readmission. GDM was associated with lower odds of readmission.

## Discussion

This study demonstrates substantial variation in postpartum care utilization across high-risk pregnancy conditions within a large urban safety-net health system. Although individuals with HDP, pregestational diabetes, and mental health conditions were more likely to attend PPVs than those without these diagnoses, overall postpartum engagement remained suboptimal. At the same time, acute care utilization was common, particularly among individuals with HDP and pregestational diabetes. Together, these findings suggest that while higher clinical risk may prompt greater postpartum follow-up, routine PPVs alone may not be sufficient to prevent acute care encounters or mitigate ongoing postpartum morbidity.

Our findings align with and extend prior studies reporting PPV attendance rates of 50–70% among individuals with HDP, GDM and pregestational diabetes [16,20–22]. By including a broader range of high-risk conditions, we extend this work to characterize condition-specific patterns in both PPV attendance and acute care utilization. Consistent with earlier reports, individuals with HDP, pregestational diabetes, and mental health conditions demonstrated higher PPV attendance. These higher follow-up rates likely reflect greater perceived clinical risk and more explicit postpartum care recommendations, particularly for HDP where early blood pressure monitoring is emphasized. In contrast to prior work, individuals with GDM had lower odds of PPV attendance [8,21–23]. This difference may reflect variations in postpartum care pathways across conditions, with HDP and chronic medical conditions often prompting clearer postpartum management plans than GDM. Importantly, associations between high-risk conditions and PPV attendance persisted after adjusting for known predictors of PPV attendance, including mode of delivery, prenatal care utilization, insurance status and timing relative to the COVID-19 pandemic which shaped patterns of care utilization [23–26]. Although the pandemic’s association with altered care utilization is reflected in our results, its independent associations will be explored further in future analyses.

Despite relatively higher attendance among high-risk groups, overall postpartum engagement remained suboptimal, and acute care use was high. Similar to prior studies, we found that HDP, pregestational diabetes, cesarean delivery, prior mental health conditions and Medicaid were associated with postpartum ED visits and readmissions [27,28]. These associations likely reflect both higher baseline medical risk and the need for closer clinical monitoring in the postpartum period, particularly for HDP and mental health conditions which can worsen after delivery. The preventability of these encounters remains uncertain and likely varies by indication. Our findings provide condition-specific estimates of postpartum health care utilization and demonstrate that substantial acute care use occurs even among individuals who attend PPVs, suggesting that outpatient postpartum engagement alone may not address the broader clinical needs driving acute care utilization. Some acute care encounters may represent appropriate escalation of care for severe conditions, underscoring that postpartum care improvement should prioritize patient outcomes, particularly reductions in morbidity and mortality, rather than utilization alone.

These findings highlight subgroups with higher care utilization who may benefit from enhanced monitoring or care coordination. Although high-risk individuals were more likely to engage in postpartum care, attendance fell below professional recommendations that all postpartum people receive early and ongoing follow-up [1]. For individuals with HDP, chronic medical conditions or risk of postpartum depression, ACOG emphasizes timely follow-up to mitigate cardiovascular, metabolic and psychiatric complications [1]. Ensuring that PPVs translate into evidence-based care, including blood pressure monitoring, diabetes screening, mental health evaluation and connection to primary care, is essential. Our findings support ongoing policy efforts to expand postpartum care beyond a single visit, as the association between PPV attendance and increased ED visits and readmissions suggests that a single postpartum encounter may not adequately address ongoing clinical needs among individuals with high-risk pregnancies. While some acute care encounters are clinically appropriate, others may be preventable through timely, risk-tailored outpatient care and improved care transitions. Interventions such as dedicated postpartum clinics, enhanced care coordination and community health worker (CHW) support may help close these gaps and improve continuity of care after delivery [29–33].

This study establishes a baseline for postpartum care engagement and acute care utilization across multiple high-risk pregnancy conditions and raises several critical questions for future research. Future studies should examine the quality and content of postpartum care beyond visit attendance and identify modifiable factors contributing to unnecessary acute care utilization versus appropriate escalation of care. In addition, upstream prevention strategies, including optimization of preconception health and interventions addressing social determinants of health that contribute to chronic conditions such as diabetes and obesity, warrant further study, as these efforts may better support individuals entering pregnancy with these conditions and improve postpartum outcomes. Given the heterogeneity of high-risk conditions and the need for individualized care, interventional studies are needed to evaluate targeted, risk-stratified approaches, such as CHW-supported coordination and structured postpartum care models, on comprehensive postpartum care delivery and reduction of preventable acute care utilization. Longitudinal studies linking postpartum engagement and care quality to long-term cardiovascular, metabolic and mental health outcomes are also essential.

Strengths of this study include its large, racially and ethnically diverse cohort. Additionally, exclusion of those who received prenatal care at an outside institution reduced underestimation of postpartum follow-up. Adjustment for timing during the COVID-19 pandemic helped to enhance the temporal relevance of our findings. Limitations include its single-institution design, reliance on EMR data and inability to capture individual or neighborhood-level social and structural determinants of health, such as housing instability, transportation barriers, or social support, which are known to be predictors of postpartum care engagement [8]. Although insurance type was included as a proxy for socioeconomic context, it is an incomplete measure of broader societal factors affecting access and continuity of care. Postpartum blood pressure checks performed via telemedicine may not have been captured as PPVs and some individuals may have received care outside our system. Additionally, ICD-9/10 diagnosis codes were used to define high-risk conditions. While these codes were manually reviewed for clinical relevance, the codes may be applied inconsistently across providers and encounters, potentially leading to misclassification or under-ascertainment of some conditions. Finally, the definition of “high-risk” was broad and inclusive; while this reflects real-world clinical complexity, it may underestimate heterogeneity within diagnostic categories.

## Conclusions

PPV attendance alone may not be sufficient to reduce acute care utilization among individuals with high-risk pregnancies. Although individuals with HDP, pregestational diabetes, and mental health conditions demonstrated higher postpartum follow-up, substantial ED visits and readmissions persisted across these groups. These findings highlight the need for postpartum care models that extend beyond a single visit and incorporate risk-tailored monitoring, care coordination, and longitudinal follow-up to better address ongoing postpartum morbidity. Developing and evaluating such models will be essential to improving maternal health outcomes after high-risk pregnancy.

## Supplementary Material

Supp 1

Supplemental data for this article can be accessed online at https://doi.org/10.1080/14767058.2026.2663199.

## Figures and Tables

**Table 1.** Demographic and clinical characteristics of patients with high-risk versus low-risk deliveries.

	High-risk (*n* = 9435)	Low-risk (*n* = 4439)	*p* Value

Maternal age, years, (mean ± standard deviation)	34.0 ± 6.1	32.8 ± 5.9	<0.001
Race, *n* (%)			<0.001
White	555 (5.9)	341 (7.7)	
Black	3047 (32.5)	1140 (25.4)	
Other	4383 (46.5)	2129 (48.0)	
Asian	517 (5.5)	316 (7.1)	
Native American	32 (0.3)	19 (0.4)	
Pacific Islander	39 (0.4)	20 (0.5)	
Mixed race	72 (0.8)	29 (0.7)	
Declined to report	779 (8.3)	456 (10.3)	
Ethnicity, *n* (%)			<0.001
Non-Hispanic	4250 (45.0)	1973 (44.4)	
Hispanic	4441 (47.1)	1997 (45.0)	
Declined to report	744 (7.9)	469 (10.6)	
Parity^a^, *n* (%)			0.55
0	2945 (31.2)	1362 (30.7)	
≥1	6488 (68.8)	3073 (69.3)	
Insurance^b^, *n* (%)			0.04
Private	2002 (22.1)	984 (23.1)	
Medicaid	7025 (77.6)	3277 (76.8)	
Medicare	23 (0.3)	3 (0.1)	
Gestational age at delivery^a^, weeks (median, IQR)	39 (38–39.9)	39.4 (38.7–40.1)	<0.001
Preterm birth < 37 weeks^a^, *n* (%)	1102 (11.7)	200 (4.5)	<0.001
Number of prenatal visits (median, IQR)	13 (9–16)	11 (8–14)	<0.001
Mode of delivery, *n* (%)			<0.001
Vaginal	5617 (59.9)	3285 (73.2)	
Cesarean	3766 (40.1)	1206 (26.8)	
Birthweight^a^, g (mean ± SD)	3180.5 ± 603.8	3228.2 ± 465.1	<0.001
Apgar score (median, IQR)			<0.001
1 min^a^	9 (8–9)	9 (9–9)	
5 min^a^	9 (9–9)	9 (9–9)	
Admission to neonatal intensive care unit (NICU)^a^, *n* (%)	1173 (12.5)	263 (5.9)	<0.001
Infant length of hospitalization, days^a^ (median, IQR)	2 (2–3)	2 (2–3)	<0.001

Values presented as mean ± standard deviation (SD), median (IQR), or *n* (%).

a Data were missing for parity (*n* = 7), gestational age (*n* = 16), preterm birth (*n* = 16), birthweight (*n* = 22), Apgar at 1 min (*n* = 73), Apgar at 5 min (*n* = 78), NICU admission (*n* = 26), and infant length of stay (*n* = 38).

b Data were missing for insurance status (*n* = 560).

**Table 2.** Prevalence and overlap of high-risk pregnancy conditions^a^ in the study cohort.

High-risk condition	*N* (%) of total cohort (*N* = 13,874)	% of all high-risk patients (*N* = 9435)	*N* (% of column 1) with overlap^b^

At least one high-risk diagnosis	9435 (68.0)	100.0	4337 (45.9)
Obesity	4142 (29.9)	43.9	2967 (71.6)
Hypertensive disorders of pregnancy	3574 (25.8)	37.9	2488 (69.6)
Gestational diabetes	2246 (16.2)	23.8	1390 (61.9)
Mental health condition	1343 (9.7)	14.2	931 (69.3)
Postpartum hemorrhage	1012 (7.3)	12.5	687 (67.9)
Pregestational diabetes	374 (2.7)	4.0	340 (90.9)
Other common medical conditions^c^	2291 (16.5)	24.3	1854 (72.1)

Values are presented as *n* (%). Cohort includes patients who received prenatal care and delivered at our institution between 1 June 2018 and 31 May 2022.

a High-risk pregnancy status was defined using 2013 ICD-9/10 diagnosis codes derived from published literature and clinical review. Categories are not mutually exclusive; patients may be represented in more than one category. Percentages of all high-risk add to more than 100% because of overlap.

b Overlap indicates that *n* (%) who had at least one additional high-risk diagnosis.

c Includes those with cardiac conditions, moderate or severe persistent asthma, a COVID-19 diagnosis during pregnancy, antepartum vaginal bleeding, hyperthyroidism, human immunodeficiency virus, venous thromboembolic disease, sickle cell disease, placenta accreta spectrum, stillbirth in the index pregnancy, autoimmune disease, chronic kidney disease, cerebrovascular disease, or other endocrine condition (see S1 Supplemental Table).

**Table 3.** Postpartum follow-up and acute care utilization by high-risk status.

	High-risk (*n* = 9435)	Low-risk (*n* = 4439)	*p* Value

Attendance of any PPV^a^, *n* (%)	5588 (59.2)	2015 (45.4)	<0.001
Attendance of early PPV^b^, *n* (%)	3817 (40.5)	1076 (24.2)	<0.001
Number of PPV within 12 weeks (median, IQR)	1 (0–2)	0 (0–1)	<0.001
≥2 PPV^a^, *n* (%)	3194 (33.8)	940 (21.2)	<0.001
ER visit within 12 weeks^c^, *n* (%)	1593 (16.9)	615 (13.9)	<0.001
Readmission within 12 weeks^c^, *n* (%)	506 (5.4)	121 (2.7)	<0.001

a PPV, postpartum visit. Postpartum visit attendance defined as any documented outpatient visit in an obstetrics or family medicine context within 12 weeks after delivery.

b Early PPV defined as a postpartum visit occurring within 21 days after delivery.

c Emergency room (ER) visits and hospital readmissions defined as any encounter occurring within 12 weeks postpartum.

**Table 4.** Postpartum follow-up and acute care utilization by specific high-risk diagnosis.

High-risk condition	Any PPV^a^, *n* (%)	Early PPV^b^, *n* (%)	≥2 PPV, *n* (%)	ER visit^c^, *n* (%)	Readmission, *n* (%)	*p* Value^d^

Obesity (*N* = 4142)	2434 (58.8)	1653 (39.9)	1377 (33.2)	711 (17.2)^e^	229 (5.5)	<0.001
Hypertensive disorder of pregnancy (*N* = 3574)	2364 (66.1)	1796 (50.3)	1442 (40.4)	659 (18.4)	245 (6.9)	<0.001
Mental health conditions (*N* = 1343)	867 (64.6)	595 (44.3)	560 (41.7)	274 (20.4)	74 (5.5)^f^	<0.001
Gestational diabetes (*N* = 2246)	1336 (59.5)	910 (40.5)	732 (32.6)^g^	353 (15.7)^h^	103 (4.6)^i^	<0.001
Pregestational diabetes (*N* = 347)	264 (70.6)	187 (50.0)	172 (46.0)	91 (24.3)	33 (8.8)	<0.001

a PPV, postpartum visit. Postpartum visit attendance defined as any documented outpatient visit in an obstetrics or family medicine context within 12 weeks after delivery.

b Early PPV defined as a postpartum visit occurring within 21 days after delivery.

c Emergency room (ER) visits and hospital readmissions defined as any encounter occurring within 12 weeks postpartum.

d *p* Values reflect comparisons between patients with and without each diagnosis; categories are not mutually exclusive.

e *p* Value for ER visits among patients with obesity was 0.007.

f *p* Value for readmissions among patients with mental health conditions was 0.07.

g *p* Value for ≥2 visits among patients with gestational diabetes was 0.002.

h *p* Value for ER visits among patients with gestational diabetes was 0.78.

i *p* Value for readmissions among patients with gestational diabetes was 0.87.

**Table 5.** Adjusted predictors of postpartum visit attendance and emergency room visits within 12 weeks postpartum.

Predictor	aOR for PPV^a^ (95% CI)	*p* Value	aOR for ER visits^b^ (95% CI)	*p* Value

Attendance of PPV	-	-	1.42 (1.28–1.57)	<0.001
Hypertensive disorder of pregnancy	1.67 (1.53–1.83)	<0.001	1.15 (1.03–1.29)	0.01
Obesity	0.96 (0.88–1.05)	0.36	1.09 (0.98–1.21)	0.11
Diabetes				
None (reference)	1.00	-	1.00	-
Gestational	0.79 (0.70–0.89)	<0.001	0.91 (0.78–1.06)	0.33
Pre-gestational	0.87 (0.68–1.13)	0.30	1.33 (1.03–1.73)	0.03
Prior mental health condition	1.49 (1.30–1.70)	<0.001	1.31 (1.04–1.41)	0.01
Age (years)	1.01 (1.01–1.02)	0.001	0.97 (0.97–0.98)	<0.001
Race				
Other (reference)	1.00	-	1.00	-
White	0.94 (0.80–1.10)	0.43	0.86 (0.69–1.07)	0.18
Black	1.22 (1.08–1.37)	0.001	0.98 (0.84–1.14)	0.78
Asian	1.02 (0.85–1.23)	0.82	1.26 (1.00–1.57)	0.05
Native American	1.26 (0.66–2.41)	0.48	0.95 (0.42–2.16)	0.90
Pacific Islander/Hawaiian	2.00 (1.09–3.69)	0.03	2.28 (1.28–4.03)	0.01
Mixed	0.80 (0.52–1.22)	0.30	0.60 (0.31–1.17)	0.14
Declined/unknown	0.94 (0.79–1.12)	0.52	0.63 (0.48–0.82)	0.001
Ethnicity				
Non-Hispanic (reference)	1.00	-	1.00	
Hispanic	1.11 (0.99–1.24)	0.06	0.92 (0.80–1.06)	0.25
Declined/unknown	0.99 (0.83–1.19)	0.94	0.58 (0.44–0.77)	<0.001
Insurance				
Private insurance (reference)	1.00	-	1.00	
Medicaid	1.13 (1.03–1.24)	0.01	1.50 (1.32–1.71)	<0.001
Medicare	4.22 (1.38–12.9)	0.01	2.23 (0.94–1.36)	0.21
Primiparity	1.20 (1.10–1.30)	<0.001	0.98 (0.88–1.10)	0.74
Preterm birth < 37 weeks	1.29 (1.10–1.51)	0.001	1.13 (0.94–1.36)	0.21
Cesarean section	1.84 (1.70–2.00)	<0.001	1.30 (1.18–1.44)	<0.001
Intrauterine fetal demise	0.27 (0.07–1.05)	0.06	11.47 (2.86–46.01)	<0.001
Multi-gestation				
1 (reference)	1.00	-	1.00	
2	0.28 (0.20–0.40)	<0.001	0.28 (0.20–0.40)	<0.001
3	0.03 (0.00–0.23)	0.001	0.03 (0.00–0.23)	0.001
NICU admission	0.95 (0.82–1.11)	0.54	1.02 (0.85–1.23)	0.81
Infant length of stay (days)	1.01 (1.00–1.01)	0.01	1.01 (1.00–1.01)	0.04
Number of prenatal visits attended	1.12 (1.11–1.13)	<0.001	1.01 (1.00–1.02)	0.03
Gestational age at first visit (weeks)	1.01 (1.01–1.02)	<0.001	1.00 (0.99–1.01)	0.62
Covid-19				
Pre-Covid (reference)	1.00	-	1.00	
Beginning of Covid	3.51 (2.98–4.13)	<0.001	0.56 (0.45–0.69)	<0.001
Middle/end of Covid	1.94 (1.77–2.13)	<0.001	0.83 (0.73–0.94)	0.003
After Covid	1.57 (1.42–1.74)	<0.001	0.88 (0.77–1.00)	0.06

Abbreviations: aOR, adjusted odds ratio; CI, confidence interval.

a PPV: postpartum visit. Postpartum visit attendance defined as any documented outpatient obstetrics or family medicine visit within 12 weeks after delivery.

b Emergency room (ER) visits defined as any encounter within 12 weeks postpartum. Models adjusted for all predictors listed in the table.

## Data Availability

The data that support the findings of this study are not publicly available due to ethical and privacy restrictions but are available from the corresponding author upon reasonable request.
