# Supplementary material for: Postpartum care utilization among high-risk pregnancies in an urban safety-net health system
Source: J Matern Fetal Neonatal Med. Author manuscript; Available in PMC 2026 Jul 28. (PMC13409234; doi:10.1080/14767058.2026.2663199)
Supplement: Supp 1 [file NIHMS2178955-supplement-Supp_1.docx]

Supplemental Materials

Supplemental Table S1: Description of Common Medical Conditions Category in Table 1

| High-Risk Condition | N (% of total cohort) | % of all high-risk |
| --- | --- | --- |
| Other Endocrine Disorder* | 1,176 (8.5) | 12.5 |
| Antepartum Vaginal Bleeding | 3,67 (2.7) | 3.9 |
| COVID-19 Diagnosis during Pregnancy | 324 (2.3) | 3.4 |
| Cardiac Condition | 260 (1.9) | 2.8 |
| Moderate or Severe Persistent Asthma | 209 (1.5) | 2.2 |
| Hyperthyroidism | 142 (1.0) | 1.5 |
| Human Immunodeficiency Virus | 82 (0.6) | 0.9 |
| Venous Thromboembolic Disease | 85 (0.6) | 0.9 |
| Sickle Cell Disease | 76 (0.6) | 0.8 |
| Placenta Acceta Spectrum | 51 (0.4) | 0.5 |
| Stillbirth | 47 (0.3) | 0.5 |
| Autoimmune Disease | 41 (0.3) | 0.4 |
| Chronic Kidney Disease | 27 (0.2) | 0.3 |
| Cerebrovascular Disease | 21 (0.1) | 0.2 |

*This category reflects those with the ICD-9/10 code, “Endocrine, nutritional and metabolic diseases complicating pregnancy,” and does not include gestational diabetes, pregestational diabetes or thyroid disease

Supplemental Table S2: Logistic regression model for predictors of hospital readmission within 12 weeks postpartum^*^

| Variables | | |  | | |
| --- | --- | --- | --- | --- | --- |
|  | | Adjusted odds ratio | | 95% Confidence Interval | P-value |
| Attendance of any PPV^†^ within 12 weeks after delivery | | 2.56 | | 2.08-3.16 | <0.001 |
| Hypertensive disorder of pregnancy | | 1.50 | | 1.25-1.80 | <0.001 |
| Obesity | | 1.14 | | 0.96-1.37 | 0.14 |
| Diabetes | |  | |  |  |
|  | None (reference) | 1.00 | | – | – |
|  | Gestational | 0.74 | | 0.56-0.97 | 0.03 |
|  | Pre-gestational | 1.21 | | 0.81-1.81 | 0.34 |
| Prior Mental Health Condition | | 1.08 | | 0.83-1.41 | 0.54 |
| Age (years) | | 1.04 | | 1.02-1.05 | <0.001 |
| Race | |  | |  |  |
|  | Other (reference) | 1.00 | | – | – |
|  | White | 0.64 | | 0.40-1.03 | 0.06 |
|  | Black | 1.52 | | 1.16-2.00 | 0.003 |
|  | Asian | 1.70 | | 1.13-2.57 | 0.01 |
|  | Native American | 0.57 | | 0.08-4.19 | 0.58 |
|  | Pacific Islander/Hawaiian | 1.12 | | 0.34-3.64 | 0.86 |
|  | Mixed | 1.01 | | 0.37-2.81 | 0.18 |
|  | Declined/Unknown | 0.86 | | 0.53-1.36 | 0.51 |
| Ethnicity | |  | |  |  |
|  | Non-Hispanic (reference) | 1.00 | | – | – |
|  | Hispanic | 1.30 | | 0.99-1.70 | 0.05 |
|  | Declined/Unknown | 0.68 | | 0.41-1.14 | 0.14 |
| Insurance | |  | |  |  |
|  | Private Insurance (reference) | 1.00 | | – | – |
|  | Medicaid | 1.08 | | 0.88-1.32 | 0.48 |
|  | Medicare | 0.54 | | 0.07-4.05 | 0.54 |
| Primiparity | | 1.23 | | 1.02-1.50 | 0.03 |
| Preterm Birth < 37 weeks | | 1.07 | | 0.78-1.46 | 0.66 |
| Cesarean Section | | 1.08 | | 0.90-1.29 | 0.89 |
| Multi-gestation | |  | |  |  |
|  | 1 (reference) | 1.00 | | – | – |
|  | 2 | 0.85 | | 0.45-1.60 | 0.60 |
| NICU admission | | 1.08 | | 0.79-1.47 | 0.64 |
| Infant length of stay (days) | | 1.00 | | 0.99-1.01 | 0.94 |
| Number of Prenatal visits attended | | 1.02 | | 1.00-1.04 | 0.08 |
| Gestational age at first visit (weeks) | | 1.01 | | 0.99-1.02 | 0.25 |
| Covid-19 | |  | |  |  |
|  | Pre-Covid (reference) | 1.00 | | – | – |
|  | Beginning of Covid | 1.05 | | 0.76-1.46 | 0.33 |
|  | Middle/End of Covid | 1.02 | | 0.83-1.27 | 0.21 |
|  | After Covid | 1.21 | | 0.97-1.53 | 0.09 |

aOR = adjusted odds ratio; CI = confidence interval. * Readmissions defined as any encounter within 12 weeks postpartum. ^†^PPV = postpartum visit. Postpartum visit attendance defined as any documented outpatient obstetrics or family medicine visit within 12 weeks after delivery. Models adjusted for all predictors listed in the table.

Appendix 1: List of ICD-9/10 Codes Associated with High-Risk Conditions

| O10011 Pre-existing essential hypertension complicating pregnancy, first trimester |
| --- |
| O10012 Pre-existing essential hypertension complicating pregnancy, second trimester |
| O10013 Pre-existing essential hypertension complicating pregnancy, third trimester |
| O10019 Pre-existing essential hypertension complicating pregnancy, unspecified trimester |
| O1002 Pre-existing essential hypertension complicating childbirth |
| O1003 Pre-existing essential hypertension complicating the puerperium |
| O10111 Pre-existing hypertensive heart disease complicating pregnancy, first trimester |
| O10112 Pre-existing hypertensive heart disease complicating pregnancy, second trimester |
| O10113 Pre-existing hypertensive heart disease complicating pregnancy, third trimester |
| O10119 Pre-existing hypertensive heart disease complicating pregnancy, unspecified trimester |
| O1012 Pre-existing hypertensive heart disease complicating childbirth |
| O1013 Pre-existing hypertensive heart disease complicating the puerperium |
| O10211 Pre-existing hypertensive chronic kidney disease complicating pregnancy, first trimester |
| O10212 Pre-existing hypertensive chronic kidney disease complicating pregnancy, second trimester |
| O10213 Pre-existing hypertensive chronic kidney disease complicating pregnancy, third trimester |
| O10219 Pre-existing hypertensive chronic kidney disease complicating pregnancy, unspecified trimester |
| O1022 Pre-existing hypertensive chronic kidney disease complicating childbirth |
| O1023 Pre-existing hypertensive chronic kidney disease complicating the puerperium |
| O10311 Pre-existing hypertensive heart and chronic kidney disease complicating pregnancy, first trimester |
| O10312 Pre-existing hypertensive heart and chronic kidney disease complicating pregnancy, second trimester |
| O10313 Pre-existing hypertensive heart and chronic kidney disease complicating pregnancy, third trimester |
| O10319 Pre-existing hypertensive heart and chronic kidney disease complicating pregnancy, unspecified trimester |
| O1032 Pre-existing hypertensive heart and chronic kidney disease complicating childbirth |
| O1033 Pre-existing hypertensive heart and chronic kidney disease complicating the puerperium |
| O10411 Pre-existing secondary hypertension complicating pregnancy, first trimester |
| O10412 Pre-existing secondary hypertension complicating pregnancy, second trimester |
| O10413 Pre-existing secondary hypertension complicating pregnancy, third trimester |
| O10419 Pre-existing secondary hypertension complicating pregnancy, unspecified trimester |
| O1042 Pre-existing secondary hypertension complicating childbirth |
| O1043 Pre-existing secondary hypertension complicating the puerperium |
| O10911 Unspecified pre-existing hypertension complicating pregnancy, first trimester |
| O10912 Unspecified pre-existing hypertension complicating pregnancy, second trimester |
| O10913 Unspecified pre-existing hypertension complicating pregnancy, third trimester |
| O10919 Unspecified pre-existing hypertension complicating pregnancy, unspecified trimester |
| O1092 Unspecified pre-existing hypertension complicating childbirth |
| O1093 Unspecified pre-existing hypertension complicating the puerperium |
| O111 Pre-existing hypertension with pre-eclampsia, first trimester |
| O112 Pre-existing hypertension with pre-eclampsia, second trimester |
| O113 Pre-existing hypertension with pre-eclampsia, third trimester |
| O114 Pre-existing hypertension with pre-eclampsia, complicating childbirth |
| O115 Pre-existing hypertension with pre-eclampsia, complicating the puerperium |
| O119 Pre-existing hypertension with pre-eclampsia, unspecified trimester |
| O1410 Severe pre-eclampsia, unspecified trimester |
| O1412 Severe pre-eclampsia, second trimester |
| O1413 Severe pre-eclampsia, third trimester |
| O1414 Severe pre-eclampsia complicating childbirth |
| O1415 Severe pre-eclampsia, complicating the puerperium |
| O1420 HELLP syndrome (HELLP), unspecified trimester |
| O1422 HELLP syndrome (HELLP), second trimester |
| O1423 HELLP syndrome (HELLP), third trimester |
| O1424 HELLP syndrome, complicating childbirth |
| O1425 HELLP syndrome, complicating the puerperium |
| O1490 Unspecified pre-eclampsia, unspecified trimester |
| O1492 Unspecified pre-eclampsia, second trimester |
| O1493 Unspecified pre-eclampsia, third trimester |
| O1494 Unspecified pre-eclampsia, complicating childbirth |
| O1495 Unspecified pre-eclampsia, complicating the puerperium |
| O1500 Eclampsia complicating pregnancy, unspecified trimester |
| O1502 Eclampsia complicating pregnancy, second trimester |
| O1503 Eclampsia complicating pregnancy, third trimester |
| O151 Eclampsia complicating labor |
| O152 Eclampsia complicating the puerperium |
| O159 Eclampsia, unspecified as to time period |
| O2250 Cerebral venous thrombosis in pregnancy, unspecified trimester |
| O2251 Cerebral venous thrombosis in pregnancy, first trimester |
| O2252 Cerebral venous thrombosis in pregnancy, second trimester |
| O2253 Cerebral venous thrombosis in pregnancy, third trimester |
| O24011 Pre-existing type 1 diabetes mellitus, in pregnancy, first trimester |
| O24012 Pre-existing type 1 diabetes mellitus, in pregnancy, second trimester |
| O24013 Pre-existing type 1 diabetes mellitus, in pregnancy, third trimester |
| O24019 Pre-existing type 1 diabetes mellitus, in pregnancy, unspecified trimester |
| O2402 Pre-existing type 1 diabetes mellitus, in childbirth |
| O2403 Pre-existing type 1 diabetes mellitus, in the puerperium |
| O24111 Pre-existing type 2 diabetes mellitus, in pregnancy, first trimester |
| O24112 Pre-existing type 2 diabetes mellitus, in pregnancy, second trimester |
| O24113 Pre-existing type 2 diabetes mellitus, in pregnancy, third trimester |
| O24119 Pre-existing type 2 diabetes mellitus, in pregnancy, unspecified trimester |
| O2412 Pre-existing type 2 diabetes mellitus, in childbirth |
| O2413 Pre-existing type 2 diabetes mellitus, in the puerperium |
| O24311 Unspecified pre-existing diabetes mellitus in pregnancy, first trimester |
| O24312 Unspecified pre-existing diabetes mellitus in pregnancy, second trimester |
| O24313 Unspecified pre-existing diabetes mellitus in pregnancy, third trimester |
| O24319 Unspecified pre-existing diabetes mellitus in pregnancy, unspecified trimester |
| O2432 Unspecified pre-existing diabetes mellitus in childbirth |
| O2433 Unspecified pre-existing diabetes mellitus in the puerperium |
| O24811 Other pre-existing diabetes mellitus in pregnancy, first trimester |
| O24812 Other pre-existing diabetes mellitus in pregnancy, second trimester |
| O24813 Other pre-existing diabetes mellitus in pregnancy, third trimester |
| O24819 Other pre-existing diabetes mellitus in pregnancy, unspecified trimester |
| O2482 Other pre-existing diabetes mellitus in childbirth |
| O2483 Other pre-existing diabetes mellitus in the puerperium |
| O24911 Unspecified diabetes mellitus in pregnancy, first trimester |
| O24912 Unspecified diabetes mellitus in pregnancy, second trimester |
| O24913 Unspecified diabetes mellitus in pregnancy, third trimester |
| O24919 Unspecified diabetes mellitus in pregnancy, unspecified trimester |
| O2492 Unspecified diabetes mellitus in childbirth |
| O2493 Unspecified diabetes mellitus in the puerperium |
| O29011 Aspiration pneumonitis due to anesthesia during pregnancy, first trimester |
| O29012 Aspiration pneumonitis due to anesthesia during pregnancy, second trimester |
| O29013 Aspiration pneumonitis due to anesthesia during pregnancy, third trimester |
| O29019 Aspiration pneumonitis due to anesthesia during pregnancy, unspecified trimester |
| O29021 Pressure collapse of lung due to anesthesia during pregnancy, first trimester |
| O29022 Pressure collapse of lung due to anesthesia during pregnancy, second trimester |
| O29023 Pressure collapse of lung due to anesthesia during pregnancy, third trimester |
| O29029 Pressure collapse of lung due to anesthesia during pregnancy, unspecified trimester |
| O29091 Other pulmonary complications of anesthesia during pregnancy, first trimester |
| O29092 Other pulmonary complications of anesthesia during pregnancy, second trimester |
| O29093 Other pulmonary complications of anesthesia during pregnancy, third trimester |
| O29099 Other pulmonary complications of anesthesia during pregnancy, unspecified trimester |
| O29111 Cardiac arrest due to anesthesia during pregnancy, first trimester |
| O29112 Cardiac arrest due to anesthesia during pregnancy, second trimester |
| O29113 Cardiac arrest due to anesthesia during pregnancy, third trimester |
| O29119 Cardiac arrest due to anesthesia during pregnancy, unspecified trimester |
| O29121 Cardiac failure due to anesthesia during pregnancy, first trimester |
| O29122 Cardiac failure due to anesthesia during pregnancy, second trimester |
| O29123 Cardiac failure due to anesthesia during pregnancy, third trimester |
| O29129 Cardiac failure due to anesthesia during pregnancy, unspecified trimester |
| O29191 Other cardiac complications of anesthesia during pregnancy, first trimester |
| O29192 Other cardiac complications of anesthesia during pregnancy, second trimester |
| O29193 Other cardiac complications of anesthesia during pregnancy, third trimester |
| O29199 Other cardiac complications of anesthesia during pregnancy, unspecified trimester |
| O29211 Cerebral anoxia due to anesthesia during pregnancy, first trimester |
| O29212 Cerebral anoxia due to anesthesia during pregnancy, second trimester |
| O29213 Cerebral anoxia due to anesthesia during pregnancy, third trimester |
| O29219 Cerebral anoxia due to anesthesia during pregnancy, unspecified trimester |
| O29291 Other central nervous system complications of anesthesia during pregnancy, first trimester |
| O29292 Other central nervous system complications of anesthesia during pregnancy, second trimester |
| O29293 Other central nervous system complications of anesthesia during pregnancy, third trimester |
| O29299 Other central nervous system complications of anesthesia during pregnancy, unspecified trimester |
| O293X1 Toxic reaction to local anesthesia during pregnancy, first trimester |
| O293X2 Toxic reaction to local anesthesia during pregnancy, second trimester |
| O293X3 Toxic reaction to local anesthesia during pregnancy, third trimester |
| O293X9 Toxic reaction to local anesthesia during pregnancy, unspecified trimester |
| O364XX0 Maternal care for intrauterine death, not applicable or unspecified |
| O364XX1 Maternal care for intrauterine death, fetus 1 |
| O364XX2 Maternal care for intrauterine death, fetus 2 |
| O364XX3 Maternal care for intrauterine death, fetus 3 |
| O364XX4 Maternal care for intrauterine death, fetus 4 |
| O364XX5 Maternal care for intrauterine death, fetus 5 |
| O364XX9 Maternal care for intrauterine death, other fetus |
| O873 Cerebral venous thrombosis in the puerperium |
| O88211 Thromboembolism in pregnancy, first trimester |
| O88212 Thromboembolism in pregnancy, second trimester |
| O88213 Thromboembolism in pregnancy, third trimester |
| O88219 Thromboembolism in pregnancy, unspecified trimester |
| O8822 Thromboembolism in childbirth |
| O8823 Thromboembolism in the puerperium |
| O88811 Other embolism in pregnancy, first trimester |
| O88812 Other embolism in pregnancy, second trimester |
| O88813 Other embolism in pregnancy, third trimester |
| O88819 Other embolism in pregnancy, unspecified trimester |
| O8882 Other embolism in childbirth |
| O8883 Other embolism in the puerperium |
| O8901 Aspiration pneumonitis due to anesthesia during the puerperium |
| O8909 Other pulmonary complications of anesthesia during the puerperium |
| O891 Cardiac complications of anesthesia during the puerperium |
| O892 Central nervous system complications of anesthesia during the puerperium |
| O893 Toxic reaction to local anesthesia during the puerperium |
| O894 Spinal and epidural anesthesia-induced headache during the puerperium |
| O895 Other complications of spinal and epidural anesthesia during the puerperium |
| O896 Failed or difficult intubation for anesthesia during the puerperium |
| O898 Other complications of anesthesia during the puerperium |
| O899 Complication of anesthesia during the puerperium, unspecified |
| O903 Peripartum cardiomyopathy |
| O904 Postpartum acute kidney failure |
| O98711 Human immunodeficiency virus [HIV] disease complicating pregnancy, first trimester |
| O98712 Human immunodeficiency virus [HIV] disease complicating pregnancy, second trimester |
| O98713 Human immunodeficiency virus [HIV] disease complicating pregnancy, third trimester |
| O98719 Human immunodeficiency virus [HIV] disease complicating pregnancy, unspecified trimester |
| O9872 Human immunodeficiency virus [HIV] disease complicating childbirth |
| O9873 Human immunodeficiency virus [HIV] disease complicating the puerperium |
| O99210 Obesity complicating pregnancy, unspecified trimester |
| O99211 Obesity complicating pregnancy, first trimester |
| O99212 Obesity complicating pregnancy, second trimester |
| O99213 Obesity complicating pregnancy, third trimester |
| O99214 Obesity complicating childbirth |
| O99215 Obesity complicating the puerperium |
| O99320 Drug use complicating pregnancy, unspecified trimester |
| O99321 Drug use complicating pregnancy, first trimester |
| O99322 Drug use complicating pregnancy, second trimester |
| O99323 Drug use complicating pregnancy, third trimester |
| O99324 Drug use complicating childbirth |
| O99325 Drug use complicating the puerperium |
| O9A111 Malignant neoplasm complicating pregnancy, first trimester |
| O9A112 Malignant neoplasm complicating pregnancy, second trimester |
| O9A113 Malignant neoplasm complicating pregnancy, third trimester |
| O9A119 Malignant neoplasm complicating pregnancy, unspecified trimester |
| O9A12 Malignant neoplasm complicating childbirth |
| O9A13 Malignant neoplasm complicating the puerperium |
| P95 Stillbirth |
| Z371 Single stillbirth |
| O6010X0 Preterm labor with preterm delivery, unspecified trimester, not applicable or unspecified |
| O6010X1 Preterm labor with preterm delivery, unspecified trimester, fetus 1 |
| O6010X2 Preterm labor with preterm delivery, unspecified trimester, fetus 2 |
| O6010X3 Preterm labor with preterm delivery, unspecified trimester, fetus 3 |
| O6010X4 Preterm labor with preterm delivery, unspecified trimester, fetus 4 |
| O6010X5 Preterm labor with preterm delivery, unspecified trimester, fetus 5 |
| O6010X9 Preterm labor with preterm delivery, unspecified trimester, other fetus |
| O6012X0 Preterm labor second trimester with preterm delivery second trimester, not applicable or unspecified |
| O6012X1 Preterm labor second trimester with preterm delivery second trimester, fetus 1 |
| O6012X2 Preterm labor second trimester with preterm delivery second trimester, fetus 2 |
| O6012X3 Preterm labor second trimester with preterm delivery second trimester, fetus 3 |
| O6012X4 Preterm labor second trimester with preterm delivery second trimester, fetus 4 |
| O6012X5 Preterm labor second trimester with preterm delivery second trimester, fetus 5 |
| O6012X9 Preterm labor second trimester with preterm delivery second trimester, other fetus |
| M3210 Systemic lupus erythematosus, organ or system involvement unspecified |
| M3211 Endocarditis in systemic lupus erythematosus |
| M3212 Pericarditis in systemic lupus erythematosus |
| M3213 Lung involvement in systemic lupus erythematosus |
| M3214 Glomerular disease in systemic lupus erythematosus |
| M3215 Tubulo-interstitial nephropathy in systemic lupus erythematosus |
| M3219 Other organ or system involvement in systemic lupus erythematosus |
| M328 Other forms of systemic lupus erythematosus |
| N181 Chronic kidney disease, stage 1 |
| N182 Chronic kidney disease, stage 2 (mild) |
| N1830 Chronic kidney disease, stage 3 unspecified |
| N1831 Chronic kidney disease, stage 3a |
| N1832 Chronic kidney disease, stage 3b |
| N184 Chronic kidney disease, stage 4 (severe) |
| N185 Chronic kidney disease, stage 5 |
| N186 End stage renal disease |
| N189 Chronic kidney disease, unspecified |
| N19 Unspecified kidney failure |
| E0800 Diabetes mellitus due to underlying condition with hyperosmolarity without nonketotic hyperglycemic-hyperosmolar coma (NKHHC) |
| E0801 Diabetes mellitus due to underlying condition with hyperosmolarity with coma |
| E0810 Diabetes mellitus due to underlying condition with ketoacidosis without coma |
| E0811 Diabetes mellitus due to underlying condition with ketoacidosis with coma |
| E0821 Diabetes mellitus due to underlying condition with diabetic nephropathy |
| E0822 Diabetes mellitus due to underlying condition with diabetic chronic kidney disease |
| E0829 Diabetes mellitus due to underlying condition with other diabetic kidney complication |
| E08311 Diabetes mellitus due to underlying condition with unspecified diabetic retinopathy with macular edema |
| E08319 Diabetes mellitus due to underlying condition with unspecified diabetic retinopathy without macular edema |
| E083211 Diabetes mellitus due to underlying condition with mild nonproliferative diabetic retinopathy with macular edema, right eye |
| E083212 Diabetes mellitus due to underlying condition with mild nonproliferative diabetic retinopathy with macular edema, left eye |
| E083213 Diabetes mellitus due to underlying condition with mild nonproliferative diabetic retinopathy with macular edema, bilateral |
| E083219 Diabetes mellitus due to underlying condition with mild nonproliferative diabetic retinopathy with macular edema, unspecified eye |
| E083291 Diabetes mellitus due to underlying condition with mild nonproliferative diabetic retinopathy without macular edema, right eye |
| E083292 Diabetes mellitus due to underlying condition with mild nonproliferative diabetic retinopathy without macular edema, left eye |
| E083293 Diabetes mellitus due to underlying condition with mild nonproliferative diabetic retinopathy without macular edema, bilateral |
| E083299 Diabetes mellitus due to underlying condition with mild nonproliferative diabetic retinopathy without macular edema, unspecified eye |
| E083311 Diabetes mellitus due to underlying condition with moderate nonproliferative diabetic retinopathy with macular edema, right eye |
| E083312 Diabetes mellitus due to underlying condition with moderate nonproliferative diabetic retinopathy with macular edema, left eye |
| E083313 Diabetes mellitus due to underlying condition with moderate nonproliferative diabetic retinopathy with macular edema, bilateral |
| E083319 Diabetes mellitus due to underlying condition with moderate nonproliferative diabetic retinopathy with macular edema, unspecified eye |
| E083391 Diabetes mellitus due to underlying condition with moderate nonproliferative diabetic retinopathy without macular edema, right eye |
| E083392 Diabetes mellitus due to underlying condition with moderate nonproliferative diabetic retinopathy without macular edema, left eye |
| E083393 Diabetes mellitus due to underlying condition with moderate nonproliferative diabetic retinopathy without macular edema, bilateral |
| E083399 Diabetes mellitus due to underlying condition with moderate nonproliferative diabetic retinopathy without macular edema, unspecified eye |
| E083411 Diabetes mellitus due to underlying condition with severe nonproliferative diabetic retinopathy with macular edema, right eye |
| E083412 Diabetes mellitus due to underlying condition with severe nonproliferative diabetic retinopathy with macular edema, left eye |
| E083413 Diabetes mellitus due to underlying condition with severe nonproliferative diabetic retinopathy with macular edema, bilateral |
| E083419 Diabetes mellitus due to underlying condition with severe nonproliferative diabetic retinopathy with macular edema, unspecified eye |
| E083491 Diabetes mellitus due to underlying condition with severe nonproliferative diabetic retinopathy without macular edema, right eye |
| E083492 Diabetes mellitus due to underlying condition with severe nonproliferative diabetic retinopathy without macular edema, left eye |
| E083493 Diabetes mellitus due to underlying condition with severe nonproliferative diabetic retinopathy without macular edema, bilateral |
| E083499 Diabetes mellitus due to underlying condition with severe nonproliferative diabetic retinopathy without macular edema, unspecified eye |
| E083511 Diabetes mellitus due to underlying condition with proliferative diabetic retinopathy with macular edema, right eye |
| E083512 Diabetes mellitus due to underlying condition with proliferative diabetic retinopathy with macular edema, left eye |
| E083513 Diabetes mellitus due to underlying condition with proliferative diabetic retinopathy with macular edema, bilateral |
| E083519 Diabetes mellitus due to underlying condition with proliferative diabetic retinopathy with macular edema, unspecified eye |
| E083521 Diabetes mellitus due to underlying condition with proliferative diabetic retinopathy with traction retinal detachment involving the macula, right eye |
| E083522 Diabetes mellitus due to underlying condition with proliferative diabetic retinopathy with traction retinal detachment involving the macula, left eye |
| E083523 Diabetes mellitus due to underlying condition with proliferative diabetic retinopathy with traction retinal detachment involving the macula, bilateral |
| E083529 Diabetes mellitus due to underlying condition with proliferative diabetic retinopathy with traction retinal detachment involving the macula, unspecified eye |
| E083531 Diabetes mellitus due to underlying condition with proliferative diabetic retinopathy with traction retinal detachment not involving the macula, right eye |
| E083532 Diabetes mellitus due to underlying condition with proliferative diabetic retinopathy with traction retinal detachment not involving the macula, left eye |
| E083533 Diabetes mellitus due to underlying condition with proliferative diabetic retinopathy with traction retinal detachment not involving the macula, bilateral |
| E083539 Diabetes mellitus due to underlying condition with proliferative diabetic retinopathy with traction retinal detachment not involving the macula, unspecified eye |
| E083541 Diabetes mellitus due to underlying condition with proliferative diabetic retinopathy with combined traction retinal detachment and rhegmatogenous retinal detachment, right eye |
| E083542 Diabetes mellitus due to underlying condition with proliferative diabetic retinopathy with combined traction retinal detachment and rhegmatogenous retinal detachment, left eye |
| E083543 Diabetes mellitus due to underlying condition with proliferative diabetic retinopathy with combined traction retinal detachment and rhegmatogenous retinal detachment, bilateral |
| E083549 Diabetes mellitus due to underlying condition with proliferative diabetic retinopathy with combined traction retinal detachment and rhegmatogenous retinal detachment, unspecified eye |
| E083551 Diabetes mellitus due to underlying condition with stable proliferative diabetic retinopathy, right eye |
| E083552 Diabetes mellitus due to underlying condition with stable proliferative diabetic retinopathy, left eye |
| E083553 Diabetes mellitus due to underlying condition with stable proliferative diabetic retinopathy, bilateral |
| E083559 Diabetes mellitus due to underlying condition with stable proliferative diabetic retinopathy, unspecified eye |
| E083591 Diabetes mellitus due to underlying condition with proliferative diabetic retinopathy without macular edema, right eye |
| E083592 Diabetes mellitus due to underlying condition with proliferative diabetic retinopathy without macular edema, left eye |
| E083593 Diabetes mellitus due to underlying condition with proliferative diabetic retinopathy without macular edema, bilateral |
| E083599 Diabetes mellitus due to underlying condition with proliferative diabetic retinopathy without macular edema, unspecified eye |
| E0836 Diabetes mellitus due to underlying condition with diabetic cataract |
| E0837X1 Diabetes mellitus due to underlying condition with diabetic macular edema, resolved following treatment, right eye |
| E0837X2 Diabetes mellitus due to underlying condition with diabetic macular edema, resolved following treatment, left eye |
| E0837X3 Diabetes mellitus due to underlying condition with diabetic macular edema, resolved following treatment, bilateral |
| E0837X9 Diabetes mellitus due to underlying condition with diabetic macular edema, resolved following treatment, unspecified eye |
| E0839 Diabetes mellitus due to underlying condition with other diabetic ophthalmic complication |
| E0840 Diabetes mellitus due to underlying condition with diabetic neuropathy, unspecified |
| E0841 Diabetes mellitus due to underlying condition with diabetic mononeuropathy |
| E0842 Diabetes mellitus due to underlying condition with diabetic polyneuropathy |
| E0843 Diabetes mellitus due to underlying condition with diabetic autonomic (poly)neuropathy |
| E0844 Diabetes mellitus due to underlying condition with diabetic amyotrophy |
| E0849 Diabetes mellitus due to underlying condition with other diabetic neurological complication |
| E0851 Diabetes mellitus due to underlying condition with diabetic peripheral angiopathy without gangrene |
| E0852 Diabetes mellitus due to underlying condition with diabetic peripheral angiopathy with gangrene |
| E0859 Diabetes mellitus due to underlying condition with other circulatory complications |
| E08610 Diabetes mellitus due to underlying condition with diabetic neuropathic arthropathy |
| E08618 Diabetes mellitus due to underlying condition with other diabetic arthropathy |
| E08620 Diabetes mellitus due to underlying condition with diabetic dermatitis |
| E08621 Diabetes mellitus due to underlying condition with foot ulcer |
| E08622 Diabetes mellitus due to underlying condition with other skin ulcer |
| E08628 Diabetes mellitus due to underlying condition with other skin complications |
| E08630 Diabetes mellitus due to underlying condition with periodontal disease |
| E08638 Diabetes mellitus due to underlying condition with other oral complications |
| E08641 Diabetes mellitus due to underlying condition with hypoglycemia with coma |
| E08649 Diabetes mellitus due to underlying condition with hypoglycemia without coma |
| E0865 Diabetes mellitus due to underlying condition with hyperglycemia |
| E0869 Diabetes mellitus due to underlying condition with other specified complication |
| E088 Diabetes mellitus due to underlying condition with unspecified complications |
| E089 Diabetes mellitus due to underlying condition without complications |
| E1010 Type 1 diabetes mellitus with ketoacidosis without coma |
| E1011 Type 1 diabetes mellitus with ketoacidosis with coma |
| E1021 Type 1 diabetes mellitus with diabetic nephropathy |
| E1022 Type 1 diabetes mellitus with diabetic chronic kidney disease |
| E1029 Type 1 diabetes mellitus with other diabetic kidney complication |
| E10311 Type 1 diabetes mellitus with unspecified diabetic retinopathy with macular edema |
| E10319 Type 1 diabetes mellitus with unspecified diabetic retinopathy without macular edema |
| E103211 Type 1 diabetes mellitus with mild nonproliferative diabetic retinopathy with macular edema, right eye |
| E103212 Type 1 diabetes mellitus with mild nonproliferative diabetic retinopathy with macular edema, left eye |
| E103213 Type 1 diabetes mellitus with mild nonproliferative diabetic retinopathy with macular edema, bilateral |
| E103219 Type 1 diabetes mellitus with mild nonproliferative diabetic retinopathy with macular edema, unspecified eye |
| E103291 Type 1 diabetes mellitus with mild nonproliferative diabetic retinopathy without macular edema, right eye |
| E103292 Type 1 diabetes mellitus with mild nonproliferative diabetic retinopathy without macular edema, left eye |
| E103293 Type 1 diabetes mellitus with mild nonproliferative diabetic retinopathy without macular edema, bilateral |
| E103299 Type 1 diabetes mellitus with mild nonproliferative diabetic retinopathy without macular edema, unspecified eye |
| E103311 Type 1 diabetes mellitus with moderate nonproliferative diabetic retinopathy with macular edema, right eye |
| E103312 Type 1 diabetes mellitus with moderate nonproliferative diabetic retinopathy with macular edema, left eye |
| E103313 Type 1 diabetes mellitus with moderate nonproliferative diabetic retinopathy with macular edema, bilateral |
| E103319 Type 1 diabetes mellitus with moderate nonproliferative diabetic retinopathy with macular edema, unspecified eye |
| E103391 Type 1 diabetes mellitus with moderate nonproliferative diabetic retinopathy without macular edema, right eye |
| E103392 Type 1 diabetes mellitus with moderate nonproliferative diabetic retinopathy without macular edema, left eye |
| E103393 Type 1 diabetes mellitus with moderate nonproliferative diabetic retinopathy without macular edema, bilateral |
| E103399 Type 1 diabetes mellitus with moderate nonproliferative diabetic retinopathy without macular edema, unspecified eye |
| E103411 Type 1 diabetes mellitus with severe nonproliferative diabetic retinopathy with macular edema, right eye |
| E103412 Type 1 diabetes mellitus with severe nonproliferative diabetic retinopathy with macular edema, left eye |
| E103413 Type 1 diabetes mellitus with severe nonproliferative diabetic retinopathy with macular edema, bilateral |
| E103419 Type 1 diabetes mellitus with severe nonproliferative diabetic retinopathy with macular edema, unspecified eye |
| E103491 Type 1 diabetes mellitus with severe nonproliferative diabetic retinopathy without macular edema, right eye |
| E103492 Type 1 diabetes mellitus with severe nonproliferative diabetic retinopathy without macular edema, left eye |
| E103493 Type 1 diabetes mellitus with severe nonproliferative diabetic retinopathy without macular edema, bilateral |
| E103499 Type 1 diabetes mellitus with severe nonproliferative diabetic retinopathy without macular edema, unspecified eye |
| E103511 Type 1 diabetes mellitus with proliferative diabetic retinopathy with macular edema, right eye |
| E103512 Type 1 diabetes mellitus with proliferative diabetic retinopathy with macular edema, left eye |
| E103513 Type 1 diabetes mellitus with proliferative diabetic retinopathy with macular edema, bilateral |
| E103519 Type 1 diabetes mellitus with proliferative diabetic retinopathy with macular edema, unspecified eye |
| E103521 Type 1 diabetes mellitus with proliferative diabetic retinopathy with traction retinal detachment involving the macula, right eye |
| E103522 Type 1 diabetes mellitus with proliferative diabetic retinopathy with traction retinal detachment involving the macula, left eye |
| E103523 Type 1 diabetes mellitus with proliferative diabetic retinopathy with traction retinal detachment involving the macula, bilateral |
| E103529 Type 1 diabetes mellitus with proliferative diabetic retinopathy with traction retinal detachment involving the macula, unspecified eye |
| E103531 Type 1 diabetes mellitus with proliferative diabetic retinopathy with traction retinal detachment not involving the macula, right eye |
| E103532 Type 1 diabetes mellitus with proliferative diabetic retinopathy with traction retinal detachment not involving the macula, left eye |
| E103533 Type 1 diabetes mellitus with proliferative diabetic retinopathy with traction retinal detachment not involving the macula, bilateral |
| E103539 Type 1 diabetes mellitus with proliferative diabetic retinopathy with traction retinal detachment not involving the macula, unspecified eye |
| E103541 Type 1 diabetes mellitus with proliferative diabetic retinopathy with combined traction retinal detachment and rhegmatogenous retinal detachment, right eye |
| E103542 Type 1 diabetes mellitus with proliferative diabetic retinopathy with combined traction retinal detachment and rhegmatogenous retinal detachment, left eye |
| E103543 Type 1 diabetes mellitus with proliferative diabetic retinopathy with combined traction retinal detachment and rhegmatogenous retinal detachment, bilateral |
| E103549 Type 1 diabetes mellitus with proliferative diabetic retinopathy with combined traction retinal detachment and rhegmatogenous retinal detachment, unspecified eye |
| E103551 Type 1 diabetes mellitus with stable proliferative diabetic retinopathy, right eye |
| E103552 Type 1 diabetes mellitus with stable proliferative diabetic retinopathy, left eye |
| E103553 Type 1 diabetes mellitus with stable proliferative diabetic retinopathy, bilateral |
| E103559 Type 1 diabetes mellitus with stable proliferative diabetic retinopathy, unspecified eye |
| E103591 Type 1 diabetes mellitus with proliferative diabetic retinopathy without macular edema, right eye |
| E103592 Type 1 diabetes mellitus with proliferative diabetic retinopathy without macular edema, left eye |
| E103593 Type 1 diabetes mellitus with proliferative diabetic retinopathy without macular edema, bilateral |
| E103599 Type 1 diabetes mellitus with proliferative diabetic retinopathy without macular edema, unspecified eye |
| E1036 Type 1 diabetes mellitus with diabetic cataract |
| E1037X1 Type 1 diabetes mellitus with diabetic macular edema, resolved following treatment, right eye |
| E1037X2 Type 1 diabetes mellitus with diabetic macular edema, resolved following treatment, left eye |
| E1037X3 Type 1 diabetes mellitus with diabetic macular edema, resolved following treatment, bilateral |
| E1037X9 Type 1 diabetes mellitus with diabetic macular edema, resolved following treatment, unspecified eye |
| E1039 Type 1 diabetes mellitus with other diabetic ophthalmic complication |
| E1040 Type 1 diabetes mellitus with diabetic neuropathy, unspecified |
| E1041 Type 1 diabetes mellitus with diabetic mononeuropathy |
| E1042 Type 1 diabetes mellitus with diabetic polyneuropathy |
| E1043 Type 1 diabetes mellitus with diabetic autonomic (poly)neuropathy |
| E1044 Type 1 diabetes mellitus with diabetic amyotrophy |
| E1049 Type 1 diabetes mellitus with other diabetic neurological complication |
| E1051 Type 1 diabetes mellitus with diabetic peripheral angiopathy without gangrene |
| E1052 Type 1 diabetes mellitus with diabetic peripheral angiopathy with gangrene |
| E1059 Type 1 diabetes mellitus with other circulatory complications |
| E10610 Type 1 diabetes mellitus with diabetic neuropathic arthropathy |
| E10618 Type 1 diabetes mellitus with other diabetic arthropathy |
| E10620 Type 1 diabetes mellitus with diabetic dermatitis |
| E10621 Type 1 diabetes mellitus with foot ulcer |
| E10622 Type 1 diabetes mellitus with other skin ulcer |
| E10628 Type 1 diabetes mellitus with other skin complications |
| E10630 Type 1 diabetes mellitus with periodontal disease |
| E10638 Type 1 diabetes mellitus with other oral complications |
| E10641 Type 1 diabetes mellitus with hypoglycemia with coma |
| E10649 Type 1 diabetes mellitus with hypoglycemia without coma |
| E1065 Type 1 diabetes mellitus with hyperglycemia |
| E1069 Type 1 diabetes mellitus with other specified complication |
| E108 Type 1 diabetes mellitus with unspecified complications |
| E109 Type 1 diabetes mellitus without complications |
| E1100 Type 2 diabetes mellitus with hyperosmolarity without nonketotic hyperglycemic-hyperosmolar coma (NKHHC) |
| E1101 Type 2 diabetes mellitus with hyperosmolarity with coma |
| E1110 Type 2 diabetes mellitus with ketoacidosis without coma |
| E1111 Type 2 diabetes mellitus with ketoacidosis with coma |
| E1121 Type 2 diabetes mellitus with diabetic nephropathy |
| E1122 Type 2 diabetes mellitus with diabetic chronic kidney disease |
| E1129 Type 2 diabetes mellitus with other diabetic kidney complication |
| E11311 Type 2 diabetes mellitus with unspecified diabetic retinopathy with macular edema |
| E11319 Type 2 diabetes mellitus with unspecified diabetic retinopathy without macular edema |
| E113211 Type 2 diabetes mellitus with mild nonproliferative diabetic retinopathy with macular edema, right eye |
| E113212 Type 2 diabetes mellitus with mild nonproliferative diabetic retinopathy with macular edema, left eye |
| E113213 Type 2 diabetes mellitus with mild nonproliferative diabetic retinopathy with macular edema, bilateral |
| E113219 Type 2 diabetes mellitus with mild nonproliferative diabetic retinopathy with macular edema, unspecified eye |
| E113291 Type 2 diabetes mellitus with mild nonproliferative diabetic retinopathy without macular edema, right eye |
| E113292 Type 2 diabetes mellitus with mild nonproliferative diabetic retinopathy without macular edema, left eye |
| E113293 Type 2 diabetes mellitus with mild nonproliferative diabetic retinopathy without macular edema, bilateral |
| E113299 Type 2 diabetes mellitus with mild nonproliferative diabetic retinopathy without macular edema, unspecified eye |
| E113311 Type 2 diabetes mellitus with moderate nonproliferative diabetic retinopathy with macular edema, right eye |
| E113312 Type 2 diabetes mellitus with moderate nonproliferative diabetic retinopathy with macular edema, left eye |
| E113313 Type 2 diabetes mellitus with moderate nonproliferative diabetic retinopathy with macular edema, bilateral |
| E113319 Type 2 diabetes mellitus with moderate nonproliferative diabetic retinopathy with macular edema, unspecified eye |
| E113391 Type 2 diabetes mellitus with moderate nonproliferative diabetic retinopathy without macular edema, right eye |
| E113392 Type 2 diabetes mellitus with moderate nonproliferative diabetic retinopathy without macular edema, left eye |
| E113393 Type 2 diabetes mellitus with moderate nonproliferative diabetic retinopathy without macular edema, bilateral |
| E113399 Type 2 diabetes mellitus with moderate nonproliferative diabetic retinopathy without macular edema, unspecified eye |
| E113411 Type 2 diabetes mellitus with severe nonproliferative diabetic retinopathy with macular edema, right eye |
| E113412 Type 2 diabetes mellitus with severe nonproliferative diabetic retinopathy with macular edema, left eye |
| E113413 Type 2 diabetes mellitus with severe nonproliferative diabetic retinopathy with macular edema, bilateral |
| E113419 Type 2 diabetes mellitus with severe nonproliferative diabetic retinopathy with macular edema, unspecified eye |
| E113491 Type 2 diabetes mellitus with severe nonproliferative diabetic retinopathy without macular edema, right eye |
| E113492 Type 2 diabetes mellitus with severe nonproliferative diabetic retinopathy without macular edema, left eye |
| E113493 Type 2 diabetes mellitus with severe nonproliferative diabetic retinopathy without macular edema, bilateral |
| E113499 Type 2 diabetes mellitus with severe nonproliferative diabetic retinopathy without macular edema, unspecified eye |
| E113511 Type 2 diabetes mellitus with proliferative diabetic retinopathy with macular edema, right eye |
| E113512 Type 2 diabetes mellitus with proliferative diabetic retinopathy with macular edema, left eye |
| E113513 Type 2 diabetes mellitus with proliferative diabetic retinopathy with macular edema, bilateral |
| E113519 Type 2 diabetes mellitus with proliferative diabetic retinopathy with macular edema, unspecified eye |
| E113521 Type 2 diabetes mellitus with proliferative diabetic retinopathy with traction retinal detachment involving the macula, right eye |
| E113522 Type 2 diabetes mellitus with proliferative diabetic retinopathy with traction retinal detachment involving the macula, left eye |
| E113523 Type 2 diabetes mellitus with proliferative diabetic retinopathy with traction retinal detachment involving the macula, bilateral |
| E113529 Type 2 diabetes mellitus with proliferative diabetic retinopathy with traction retinal detachment involving the macula, unspecified eye |
| E113531 Type 2 diabetes mellitus with proliferative diabetic retinopathy with traction retinal detachment not involving the macula, right eye |
| E113532 Type 2 diabetes mellitus with proliferative diabetic retinopathy with traction retinal detachment not involving the macula, left eye |
| E113533 Type 2 diabetes mellitus with proliferative diabetic retinopathy with traction retinal detachment not involving the macula, bilateral |
| E113539 Type 2 diabetes mellitus with proliferative diabetic retinopathy with traction retinal detachment not involving the macula, unspecified eye |
| E113541 Type 2 diabetes mellitus with proliferative diabetic retinopathy with combined traction retinal detachment and rhegmatogenous retinal detachment, right eye |
| E113542 Type 2 diabetes mellitus with proliferative diabetic retinopathy with combined traction retinal detachment and rhegmatogenous retinal detachment, left eye |
| E113543 Type 2 diabetes mellitus with proliferative diabetic retinopathy with combined traction retinal detachment and rhegmatogenous retinal detachment, bilateral |
| E113549 Type 2 diabetes mellitus with proliferative diabetic retinopathy with combined traction retinal detachment and rhegmatogenous retinal detachment, unspecified eye |
| E113551 Type 2 diabetes mellitus with stable proliferative diabetic retinopathy, right eye |
| E113552 Type 2 diabetes mellitus with stable proliferative diabetic retinopathy, left eye |
| E113553 Type 2 diabetes mellitus with stable proliferative diabetic retinopathy, bilateral |
| E113559 Type 2 diabetes mellitus with stable proliferative diabetic retinopathy, unspecified eye |
| E113591 Type 2 diabetes mellitus with proliferative diabetic retinopathy without macular edema, right eye |
| E113592 Type 2 diabetes mellitus with proliferative diabetic retinopathy without macular edema, left eye |
| E113593 Type 2 diabetes mellitus with proliferative diabetic retinopathy without macular edema, bilateral |
| E113599 Type 2 diabetes mellitus with proliferative diabetic retinopathy without macular edema, unspecified eye |
| E1136 Type 2 diabetes mellitus with diabetic cataract |
| E1137X1 Type 2 diabetes mellitus with diabetic macular edema, resolved following treatment, right eye |
| E1137X2 Type 2 diabetes mellitus with diabetic macular edema, resolved following treatment, left eye |
| E1137X3 Type 2 diabetes mellitus with diabetic macular edema, resolved following treatment, bilateral |
| E1137X9 Type 2 diabetes mellitus with diabetic macular edema, resolved following treatment, unspecified eye |
| E1139 Type 2 diabetes mellitus with other diabetic ophthalmic complication |
| E1140 Type 2 diabetes mellitus with diabetic neuropathy, unspecified |
| E1141 Type 2 diabetes mellitus with diabetic mononeuropathy |
| E1142 Type 2 diabetes mellitus with diabetic polyneuropathy |
| E1143 Type 2 diabetes mellitus with diabetic autonomic (poly)neuropathy |
| E1144 Type 2 diabetes mellitus with diabetic amyotrophy |
| E1149 Type 2 diabetes mellitus with other diabetic neurological complication |
| E1151 Type 2 diabetes mellitus with diabetic peripheral angiopathy without gangrene |
| E1152 Type 2 diabetes mellitus with diabetic peripheral angiopathy with gangrene |
| E1159 Type 2 diabetes mellitus with other circulatory complications |
| E11610 Type 2 diabetes mellitus with diabetic neuropathic arthropathy |
| E11618 Type 2 diabetes mellitus with other diabetic arthropathy |
| E11620 Type 2 diabetes mellitus with diabetic dermatitis |
| E11621 Type 2 diabetes mellitus with foot ulcer |
| E11622 Type 2 diabetes mellitus with other skin ulcer |
| E11628 Type 2 diabetes mellitus with other skin complications |
| E11630 Type 2 diabetes mellitus with periodontal disease |
| E11638 Type 2 diabetes mellitus with other oral complications |
| E11641 Type 2 diabetes mellitus with hypoglycemia with coma |
| E11649 Type 2 diabetes mellitus with hypoglycemia without coma |
| E1165 Type 2 diabetes mellitus with hyperglycemia |
| E1169 Type 2 diabetes mellitus with other specified complication |
| E118 Type 2 diabetes mellitus with unspecified complications |
| E119 Type 2 diabetes mellitus without complications |
| O24410 Gestational diabetes mellitus in pregnancy, diet controlled |
| O24414 Gestational diabetes mellitus in pregnancy, insulin controlled |
| O24415 Gestational diabetes mellitus in pregnancy, controlled by oral hypoglycemic drugs |
| O24419 Gestational diabetes mellitus in pregnancy, unspecified control |
| O24420 Gestational diabetes mellitus in childbirth, diet controlled |
| O24424 Gestational diabetes mellitus in childbirth, insulin controlled |
| O24425 Gestational diabetes mellitus in childbirth, controlled by oral hypoglycemic drugs |
| O24429 Gestational diabetes mellitus in childbirth, unspecified control |
| O24430 Gestational diabetes mellitus in the puerperium, diet controlled |
| O24434 Gestational diabetes mellitus in the puerperium, insulin controlled |
| O24435 Gestational diabetes mellitus in puerperium, controlled by oral hypoglycemic drugs |
| O24439 Gestational diabetes mellitus in the puerperium, unspecified control |
| D571 Sickle-cell disease without crisis |
| D5720 Sickle-cell/Hb-C disease without crisis |
| D57211 Sickle-cell/Hb-C disease with acute chest syndrome |
| D57212 Sickle-cell/Hb-C disease with splenic sequestration |
| D57213 Sickle-cell/Hb-C disease with cerebral vascular involvement |
| D57218 Sickle-cell/Hb-C disease with crisis with other specified complication |
| D57219 Sickle-cell/Hb-C disease with crisis, unspecified |
| D5740 Sickle-cell thalassemia without crisis |
| D57411 Sickle-cell thalassemia, unspecified, with acute chest syndrome |
| D57412 Sickle-cell thalassemia, unspecified, with splenic sequestration |
| D57413 Sickle-cell thalassemia, unspecified, with cerebral vascular involvement |
| D57418 Sickle-cell thalassemia, unspecified, with crisis with other specified complication |
| D57419 Sickle-cell thalassemia, unspecified, with crisis |
| D5742 Sickle-cell thalassemia beta zero without crisis |
| D57431 Sickle-cell thalassemia beta zero with acute chest syndrome |
| D57432 Sickle-cell thalassemia beta zero with splenic sequestration |
| D57433 Sickle-cell thalassemia beta zero with cerebral vascular involvement |
| D57438 Sickle-cell thalassemia beta zero with crisis with other specified complication |
| D57439 Sickle-cell thalassemia beta zero with crisis, unspecified |
| D5744 Sickle-cell thalassemia beta plus without crisis |
| D57451 Sickle-cell thalassemia beta plus with acute chest syndrome |
| D57452 Sickle-cell thalassemia beta plus with splenic sequestration |
| D57453 Sickle-cell thalassemia beta plus with cerebral vascular involvement |
| D57458 Sickle-cell thalassemia beta plus with crisis with other specified complication |
| D57459 Sickle-cell thalassemia beta plus with crisis, unspecified |
| D5780 Other sickle-cell disorders without crisis |
| D57811 Other sickle-cell disorders with acute chest syndrome |
| D57812 Other sickle-cell disorders with splenic sequestration |
| D57813 Other sickle-cell disorders with cerebral vascular involvement |
| D57818 Other sickle-cell disorders with crisis with other specified complication |
| D57819 Other sickle-cell disorders with crisis, unspecified |
| D6861 Antiphospholipid syndrome |
| D68312 Antiphospholipid antibody with hemorrhagic disorder |
| I10 Essential (primary) hypertension |
| I110 Hypertensive heart disease with heart failure |
| I119 Hypertensive heart disease without heart failure |
| I120 Hypertensive chronic kidney disease with stage 5 chronic kidney disease or end stage renal disease |
| I129 Hypertensive chronic kidney disease with stage 1 through stage 4 chronic kidney disease, or unspecified chronic kidney disease |
| I130 Hypertensive heart and chronic kidney disease with heart failure and stage 1 through stage 4 chronic kidney disease, or unspecified chronic kidney disease |
| I1310 Hypertensive heart and chronic kidney disease without heart failure, with stage 1 through stage 4 chronic kidney disease, or unspecified chronic kidney disease |
| I1311 Hypertensive heart and chronic kidney disease without heart failure, with stage 5 chronic kidney disease, or end stage renal disease |
| I132 Hypertensive heart and chronic kidney disease with heart failure and with stage 5 chronic kidney disease, or end stage renal disease |
| I150 Renovascular hypertension |
| I151 Hypertension secondary to other renal disorders |
| I152 Hypertension secondary to endocrine disorders |
| I158 Other secondary hypertension |
| I159 Secondary hypertension, unspecified |
| I160 Hypertensive urgency |
| I161 Hypertensive emergency |
| I169 Hypertensive crisis, unspecified |
| I2601 Septic pulmonary embolism with acute cor pulmonale |
| I2602 Saddle embolus of pulmonary artery with acute cor pulmonale |
| I2609 Other pulmonary embolism with acute cor pulmonale |
| I2690 Septic pulmonary embolism without acute cor pulmonale |
| I2692 Saddle embolus of pulmonary artery without acute cor pulmonale |
| I2693 Single subsegmental pulmonary embolism without acute cor pulmonale |
| I2694 Multiple subsegmental pulmonary emboli without acute cor pulmonale |
| I2699 Other pulmonary embolism without acute cor pulmonale |
| I270 Primary pulmonary hypertension |
| I2720 Pulmonary hypertension, unspecified |
| I2721 Secondary pulmonary arterial hypertension |
| I2722 Pulmonary hypertension due to left heart disease |
| I2723 Pulmonary hypertension due to lung diseases and hypoxia |
| I2724 Chronic thromboembolic pulmonary hypertension |
| I2729 Other secondary pulmonary hypertension |
| I2781 Cor pulmonale (chronic) |
| I2782 Chronic pulmonary embolism |
| I2783 Eisenmenger's syndrome |
| I2789 Other specified pulmonary heart diseases |
| I279 Pulmonary heart disease, unspecified |
| F200 Paranoid schizophrenia |
| F201 Disorganized schizophrenia |
| F202 Catatonic schizophrenia |
| F203 Undifferentiated schizophrenia |
| F205 Residual schizophrenia |
| F2081 Schizophreniform disorder |
| F2089 Other schizophrenia |
| F209 Schizophrenia, unspecified |
| F21 Schizotypal disorder |
| F22 Delusional disorders |
| F23 Brief psychotic disorder |
| F24 Shared psychotic disorder |
| F250 Schizoaffective disorder, bipolar type |
| F251 Schizoaffective disorder, depressive type |
| F258 Other schizoaffective disorders |
| F259 Schizoaffective disorder, unspecified |
| F28 Other psychotic disorder not due to a substance or known physiological condition |
| F29 Unspecified psychosis not due to a substance or known physiological condition |
| F3010 Manic episode without psychotic symptoms, unspecified |
| F3011 Manic episode without psychotic symptoms, mild |
| F3012 Manic episode without psychotic symptoms, moderate |
| F3013 Manic episode, severe, without psychotic symptoms |
| F302 Manic episode, severe with psychotic symptoms |
| F303 Manic episode in partial remission |
| F304 Manic episode in full remission |
| F308 Other manic episodes |
| F309 Manic episode, unspecified |
| F310 Bipolar disorder, current episode hypomanic |
| F3110 Bipolar disorder, current episode manic without psychotic features, unspecified |
| F3111 Bipolar disorder, current episode manic without psychotic features, mild |
| F3112 Bipolar disorder, current episode manic without psychotic features, moderate |
| F3113 Bipolar disorder, current episode manic without psychotic features, severe |
| F312 Bipolar disorder, current episode manic severe with psychotic features |
| F3130 Bipolar disorder, current episode depressed, mild or moderate severity, unspecified |
| F3131 Bipolar disorder, current episode depressed, mild |
| F3132 Bipolar disorder, current episode depressed, moderate |
| F314 Bipolar disorder, current episode depressed, severe, without psychotic features |
| F315 Bipolar disorder, current episode depressed, severe, with psychotic features |
| F3160 Bipolar disorder, current episode mixed, unspecified |
| F3161 Bipolar disorder, current episode mixed, mild |
| F3162 Bipolar disorder, current episode mixed, moderate |
| F3163 Bipolar disorder, current episode mixed, severe, without psychotic features |
| F3164 Bipolar disorder, current episode mixed, severe, with psychotic features |
| F3170 Bipolar disorder, currently in remission, most recent episode unspecified |
| F3171 Bipolar disorder, in partial remission, most recent episode hypomanic |
| F3172 Bipolar disorder, in full remission, most recent episode hypomanic |
| F3173 Bipolar disorder, in partial remission, most recent episode manic |
| F3174 Bipolar disorder, in full remission, most recent episode manic |
| F3175 Bipolar disorder, in partial remission, most recent episode depressed |
| F3176 Bipolar disorder, in full remission, most recent episode depressed |
| F3177 Bipolar disorder, in partial remission, most recent episode mixed |
| F3178 Bipolar disorder, in full remission, most recent episode mixed |
| F3181 Bipolar II disorder |
| F3189 Other bipolar disorder |
| F319 Bipolar disorder, unspecified |
| F320 Major depressive disorder, single episode, mild |
| F321 Major depressive disorder, single episode, moderate |
| F322 Major depressive disorder, single episode, severe without psychotic features |
| F323 Major depressive disorder, single episode, severe with psychotic features |
| F324 Major depressive disorder, single episode, in partial remission |
| F325 Major depressive disorder, single episode, in full remission |
| F3289 Other specified depressive episodes |
| F329 Major depressive disorder, single episode, unspecified |
| F32A Depression, unspecified |
| F330 Major depressive disorder, recurrent, mild |
| F331 Major depressive disorder, recurrent, moderate |
| F332 Major depressive disorder, recurrent severe without psychotic features |
| F333 Major depressive disorder, recurrent, severe with psychotic symptoms |
| F3340 Major depressive disorder, recurrent, in remission, unspecified |
| F3341 Major depressive disorder, recurrent, in partial remission |
| F3342 Major depressive disorder, recurrent, in full remission |
| F338 Other recurrent depressive disorders |
| F339 Major depressive disorder, recurrent, unspecified |
| I82210 Acute embolism and thrombosis of superior vena cava |
| I82211 Chronic embolism and thrombosis of superior vena cava |
| I82220 Acute embolism and thrombosis of inferior vena cava |
| I82221 Chronic embolism and thrombosis of inferior vena cava |
| I82290 Acute embolism and thrombosis of other thoracic veins |
| I82291 Chronic embolism and thrombosis of other thoracic veins |
| I823 Embolism and thrombosis of renal vein |
| I82401 Acute embolism and thrombosis of unspecified deep veins of right lower extremity |
| I82402 Acute embolism and thrombosis of unspecified deep veins of left lower extremity |
| I82403 Acute embolism and thrombosis of unspecified deep veins of lower extremity, bilateral |
| I82409 Acute embolism and thrombosis of unspecified deep veins of unspecified lower extremity |
| I82411 Acute embolism and thrombosis of right femoral vein |
| I82412 Acute embolism and thrombosis of left femoral vein |
| I82413 Acute embolism and thrombosis of femoral vein, bilateral |
| I82419 Acute embolism and thrombosis of unspecified femoral vein |
| I82421 Acute embolism and thrombosis of right iliac vein |
| I82422 Acute embolism and thrombosis of left iliac vein |
| I82423 Acute embolism and thrombosis of iliac vein, bilateral |
| I82429 Acute embolism and thrombosis of unspecified iliac vein |
| I82431 Acute embolism and thrombosis of right popliteal vein |
| I82432 Acute embolism and thrombosis of left popliteal vein |
| I82433 Acute embolism and thrombosis of popliteal vein, bilateral |
| I82439 Acute embolism and thrombosis of unspecified popliteal vein |
| I82441 Acute embolism and thrombosis of right tibial vein |
| I82442 Acute embolism and thrombosis of left tibial vein |
| I82443 Acute embolism and thrombosis of tibial vein, bilateral |
| I82449 Acute embolism and thrombosis of unspecified tibial vein |
| I82451 Acute embolism and thrombosis of right peroneal vein |
| I82452 Acute embolism and thrombosis of left peroneal vein |
| I82453 Acute embolism and thrombosis of peroneal vein, bilateral |
| I82459 Acute embolism and thrombosis of unspecified peroneal vein |
| I82461 Acute embolism and thrombosis of right calf muscular vein |
| I82462 Acute embolism and thrombosis of left calf muscular vein |
| I82463 Acute embolism and thrombosis of calf muscular vein, bilateral |
| I82469 Acute embolism and thrombosis of unspecified calf muscular vein |
| I82491 Acute embolism and thrombosis of other specified deep vein of right lower extremity |
| I82492 Acute embolism and thrombosis of other specified deep vein of left lower extremity |
| I82493 Acute embolism and thrombosis of other specified deep vein of lower extremity, bilateral |
| I82499 Acute embolism and thrombosis of other specified deep vein of unspecified lower extremity |
| I824Y1 Acute embolism and thrombosis of unspecified deep veins of right proximal lower extremity |
| I824Y2 Acute embolism and thrombosis of unspecified deep veins of left proximal lower extremity |
| I824Y3 Acute embolism and thrombosis of unspecified deep veins of proximal lower extremity, bilateral |
| I824Y9 Acute embolism and thrombosis of unspecified deep veins of unspecified proximal lower extremity |
| I824Z1 Acute embolism and thrombosis of unspecified deep veins of right distal lower extremity |
| I824Z2 Acute embolism and thrombosis of unspecified deep veins of left distal lower extremity |
| I824Z3 Acute embolism and thrombosis of unspecified deep veins of distal lower extremity, bilateral |
| I824Z9 Acute embolism and thrombosis of unspecified deep veins of unspecified distal lower extremity |
| I82501 Chronic embolism and thrombosis of unspecified deep veins of right lower extremity |
| I82502 Chronic embolism and thrombosis of unspecified deep veins of left lower extremity |
| I82503 Chronic embolism and thrombosis of unspecified deep veins of lower extremity, bilateral |
| I82509 Chronic embolism and thrombosis of unspecified deep veins of unspecified lower extremity |
| I82511 Chronic embolism and thrombosis of right femoral vein |
| I82512 Chronic embolism and thrombosis of left femoral vein |
| I82513 Chronic embolism and thrombosis of femoral vein, bilateral |
| I82519 Chronic embolism and thrombosis of unspecified femoral vein |
| I82521 Chronic embolism and thrombosis of right iliac vein |
| I82522 Chronic embolism and thrombosis of left iliac vein |
| I82523 Chronic embolism and thrombosis of iliac vein, bilateral |
| I82529 Chronic embolism and thrombosis of unspecified iliac vein |
| I82531 Chronic embolism and thrombosis of right popliteal vein |
| I82532 Chronic embolism and thrombosis of left popliteal vein |
| I82533 Chronic embolism and thrombosis of popliteal vein, bilateral |
| I82539 Chronic embolism and thrombosis of unspecified popliteal vein |
| I82541 Chronic embolism and thrombosis of right tibial vein |
| I82542 Chronic embolism and thrombosis of left tibial vein |
| I82543 Chronic embolism and thrombosis of tibial vein, bilateral |
| I82549 Chronic embolism and thrombosis of unspecified tibial vein |
| I82551 Chronic embolism and thrombosis of right peroneal vein |
| I82552 Chronic embolism and thrombosis of left peroneal vein |
| I82553 Chronic embolism and thrombosis of peroneal vein, bilateral |
| I82559 Chronic embolism and thrombosis of unspecified peroneal vein |
| I82561 Chronic embolism and thrombosis of right calf muscular vein |
| I82562 Chronic embolism and thrombosis of left calf muscular vein |
| I82563 Chronic embolism and thrombosis of calf muscular vein, bilateral |
| I82569 Chronic embolism and thrombosis of unspecified calf muscular vein |
| I82591 Chronic embolism and thrombosis of other specified deep vein of right lower extremity |
| I82592 Chronic embolism and thrombosis of other specified deep vein of left lower extremity |
| I82593 Chronic embolism and thrombosis of other specified deep vein of lower extremity, bilateral |
| I82599 Chronic embolism and thrombosis of other specified deep vein of unspecified lower extremity |
| I825Y1 Chronic embolism and thrombosis of unspecified deep veins of right proximal lower extremity |
| I825Y2 Chronic embolism and thrombosis of unspecified deep veins of left proximal lower extremity |
| I825Y3 Chronic embolism and thrombosis of unspecified deep veins of proximal lower extremity, bilateral |
| I825Y9 Chronic embolism and thrombosis of unspecified deep veins of unspecified proximal lower extremity |
| I825Z1 Chronic embolism and thrombosis of unspecified deep veins of right distal lower extremity |
| I825Z2 Chronic embolism and thrombosis of unspecified deep veins of left distal lower extremity |
| I825Z3 Chronic embolism and thrombosis of unspecified deep veins of distal lower extremity, bilateral |
| I825Z9 Chronic embolism and thrombosis of unspecified deep veins of unspecified distal lower extremity |
| I82601 Acute embolism and thrombosis of unspecified veins of right upper extremity |
| I82602 Acute embolism and thrombosis of unspecified veins of left upper extremity |
| I82603 Acute embolism and thrombosis of unspecified veins of upper extremity, bilateral |
| I82609 Acute embolism and thrombosis of unspecified veins of unspecified upper extremity |
| I82611 Acute embolism and thrombosis of superficial veins of right upper extremity |
| I82612 Acute embolism and thrombosis of superficial veins of left upper extremity |
| I82613 Acute embolism and thrombosis of superficial veins of upper extremity, bilateral |
| I82619 Acute embolism and thrombosis of superficial veins of unspecified upper extremity |
| I82621 Acute embolism and thrombosis of deep veins of right upper extremity |
| I82622 Acute embolism and thrombosis of deep veins of left upper extremity |
| I82623 Acute embolism and thrombosis of deep veins of upper extremity, bilateral |
| I82629 Acute embolism and thrombosis of deep veins of unspecified upper extremity |
| I82701 Chronic embolism and thrombosis of unspecified veins of right upper extremity |
| I82702 Chronic embolism and thrombosis of unspecified veins of left upper extremity |
| I82703 Chronic embolism and thrombosis of unspecified veins of upper extremity, bilateral |
| I82709 Chronic embolism and thrombosis of unspecified veins of unspecified upper extremity |
| I82711 Chronic embolism and thrombosis of superficial veins of right upper extremity |
| I82712 Chronic embolism and thrombosis of superficial veins of left upper extremity |
| I82713 Chronic embolism and thrombosis of superficial veins of upper extremity, bilateral |
| I82719 Chronic embolism and thrombosis of superficial veins of unspecified upper extremity |
| I82721 Chronic embolism and thrombosis of deep veins of right upper extremity |
| I82722 Chronic embolism and thrombosis of deep veins of left upper extremity |
| I82723 Chronic embolism and thrombosis of deep veins of upper extremity, bilateral |
| I82729 Chronic embolism and thrombosis of deep veins of unspecified upper extremity |
| I82A11 Acute embolism and thrombosis of right axillary vein |
| I82A12 Acute embolism and thrombosis of left axillary vein |
| I82A13 Acute embolism and thrombosis of axillary vein, bilateral |
| I82A19 Acute embolism and thrombosis of unspecified axillary vein |
| I82A21 Chronic embolism and thrombosis of right axillary vein |
| I82A22 Chronic embolism and thrombosis of left axillary vein |
| I82A23 Chronic embolism and thrombosis of axillary vein, bilateral |
| I82A29 Chronic embolism and thrombosis of unspecified axillary vein |
| I82B11 Acute embolism and thrombosis of right subclavian vein |
| I82B12 Acute embolism and thrombosis of left subclavian vein |
| I82B13 Acute embolism and thrombosis of subclavian vein, bilateral |
| I82B19 Acute embolism and thrombosis of unspecified subclavian vein |
| I82B21 Chronic embolism and thrombosis of right subclavian vein |
| I82B22 Chronic embolism and thrombosis of left subclavian vein |
| I82B23 Chronic embolism and thrombosis of subclavian vein, bilateral |
| I82B29 Chronic embolism and thrombosis of unspecified subclavian vein |
| I82C11 Acute embolism and thrombosis of right internal jugular vein |
| I82C12 Acute embolism and thrombosis of left internal jugular vein |
| I82C13 Acute embolism and thrombosis of internal jugular vein, bilateral |
| I82C19 Acute embolism and thrombosis of unspecified internal jugular vein |
| I82C21 Chronic embolism and thrombosis of right internal jugular vein |
| I82C22 Chronic embolism and thrombosis of left internal jugular vein |
| I82C23 Chronic embolism and thrombosis of internal jugular vein, bilateral |
| I82C29 Chronic embolism and thrombosis of unspecified internal jugular vein |
| I82811 Embolism and thrombosis of superficial veins of right lower extremity |
| I82812 Embolism and thrombosis of superficial veins of left lower extremity |
| I82813 Embolism and thrombosis of superficial veins of lower extremities, bilateral |
| I82819 Embolism and thrombosis of superficial veins of unspecified lower extremity |
| I82890 Acute embolism and thrombosis of other specified veins |
| I82891 Chronic embolism and thrombosis of other specified veins |
| I8290 Acute embolism and thrombosis of unspecified vein |
| I8291 Chronic embolism and thrombosis of unspecified vein |
| E0500 Thyrotoxicosis with diffuse goiter without thyrotoxic crisis or storm |
| E0501 Thyrotoxicosis with diffuse goiter with thyrotoxic crisis or storm |
| E0510 Thyrotoxicosis with toxic single thyroid nodule without thyrotoxic crisis or storm |
| E0511 Thyrotoxicosis with toxic single thyroid nodule with thyrotoxic crisis or storm |
| E0520 Thyrotoxicosis with toxic multinodular goiter without thyrotoxic crisis or storm |
| E0521 Thyrotoxicosis with toxic multinodular goiter with thyrotoxic crisis or storm |
| E0530 Thyrotoxicosis from ectopic thyroid tissue without thyrotoxic crisis or storm |
| E0531 Thyrotoxicosis from ectopic thyroid tissue with thyrotoxic crisis or storm |
| E0540 Thyrotoxicosis factitia without thyrotoxic crisis or storm |
| E0541 Thyrotoxicosis factitia with thyrotoxic crisis or storm |
| E0580 Other thyrotoxicosis without thyrotoxic crisis or storm |
| E0581 Other thyrotoxicosis with thyrotoxic crisis or storm |
| E0590 Thyrotoxicosis, unspecified without thyrotoxic crisis or storm |
| E0591 Thyrotoxicosis, unspecified with thyrotoxic crisis or storm |
| I2101 ST elevation (STEMI) myocardial infarction involving left main coronary artery |
| I2102 ST elevation (STEMI) myocardial infarction involving left anterior descending coronary artery |
| I2109 ST elevation (STEMI) myocardial infarction involving other coronary artery of anterior wall |
| I2111 ST elevation (STEMI) myocardial infarction involving right coronary artery |
| I2119 ST elevation (STEMI) myocardial infarction involving other coronary artery of inferior wall |
| I2121 ST elevation (STEMI) myocardial infarction involving left circumflex coronary artery |
| I2129 ST elevation (STEMI) myocardial infarction involving other sites |
| I213 ST elevation (STEMI) myocardial infarction of unspecified site |
| I214 Non-ST elevation (NSTEMI) myocardial infarction |
| I219 Acute myocardial infarction, unspecified |
| I21A1 Myocardial infarction type 2 |
| I21A9 Other myocardial infarction type |
| I220 Subsequent ST elevation (STEMI) myocardial infarction of anterior wall |
| I221 Subsequent ST elevation (STEMI) myocardial infarction of inferior wall |
| I222 Subsequent non-ST elevation (NSTEMI) myocardial infarction |
| I228 Subsequent ST elevation (STEMI) myocardial infarction of other sites |
| I229 Subsequent ST elevation (STEMI) myocardial infarction of unspecified site |
| I249 Acute ischemic heart disease, unspecified |
| I2510 Atherosclerotic heart disease of native coronary artery without angina pectoris |
| I25110 Atherosclerotic heart disease of native coronary artery with unstable angina pectoris |
| I25111 Atherosclerotic heart disease of native coronary artery with angina pectoris with documented spasm |
| I25118 Atherosclerotic heart disease of native coronary artery with other forms of angina pectoris |
| I25119 Atherosclerotic heart disease of native coronary artery with unspecified angina pectoris |
| I2541 Coronary artery aneurysm |
| I2542 Coronary artery dissection |
| I255 Ischemic cardiomyopathy |
| I342 Nonrheumatic mitral (valve) stenosis |
| I330 Acute and subacute infective endocarditis |
| I339 Acute and subacute endocarditis, unspecified |
| I340 Nonrheumatic mitral (valve) insufficiency |
| I342 Nonrheumatic mitral (valve) stenosis |
| I348 Other nonrheumatic mitral valve disorders |
| I349 Nonrheumatic mitral valve disorder, unspecified |
| I350 Nonrheumatic aortic (valve) stenosis |
| I351 Nonrheumatic aortic (valve) insufficiency |
| I352 Nonrheumatic aortic (valve) stenosis with insufficiency |
| I358 Other nonrheumatic aortic valve disorders |
| I359 Nonrheumatic aortic valve disorder, unspecified |
| I360 Nonrheumatic tricuspid (valve) stenosis |
| I361 Nonrheumatic tricuspid (valve) insufficiency |
| I362 Nonrheumatic tricuspid (valve) stenosis with insufficiency |
| I368 Other nonrheumatic tricuspid valve disorders |
| I369 Nonrheumatic tricuspid valve disorder, unspecified |
| I370 Nonrheumatic pulmonary valve stenosis |
| I371 Nonrheumatic pulmonary valve insufficiency |
| I372 Nonrheumatic pulmonary valve stenosis with insufficiency |
| I378 Other nonrheumatic pulmonary valve disorders |
| I379 Nonrheumatic pulmonary valve disorder, unspecified |
| I38 Endocarditis, valve unspecified |
| I39 Endocarditis and heart valve disorders in diseases classified elsewhere |
| I400 Infective myocarditis |
| I401 Isolated myocarditis |
| I408 Other acute myocarditis |
| I409 Acute myocarditis, unspecified |
| I41 Myocarditis in diseases classified elsewhere |
| I420 Dilated cardiomyopathy |
| I421 Obstructive hypertrophic cardiomyopathy |
| I422 Other hypertrophic cardiomyopathy |
| I423 Endomyocardial (eosinophilic) disease |
| I424 Endocardial fibroelastosis |
| I425 Other restrictive cardiomyopathy |
| I426 Alcoholic cardiomyopathy |
| I427 Cardiomyopathy due to drug and external agent |
| I428 Other cardiomyopathies |
| I429 Cardiomyopathy, unspecified |
| I43 Cardiomyopathy in diseases classified elsewhere |
| I440 Atrioventricular block, first degree |
| I441 Atrioventricular block, second degree |
| I442 Atrioventricular block, complete |
| I4430 Unspecified atrioventricular block |
| I4439 Other atrioventricular block |
| I444 Left anterior fascicular block |
| I445 Left posterior fascicular block |
| I4460 Unspecified fascicular block |
| I4469 Other fascicular block |
| I447 Left bundle-branch block, unspecified |
| I450 Right fascicular block |
| I4510 Unspecified right bundle-branch block |
| I4519 Other right bundle-branch block |
| I452 Bifascicular block |
| I453 Trifascicular block |
| I454 Nonspecific intraventricular block |
| I455 Other specified heart block |
| I456 Pre-excitation syndrome |
| I4581 Long QT syndrome |
| I4589 Other specified conduction disorders |
| I459 Conduction disorder, unspecified |
| I462 Cardiac arrest due to underlying cardiac condition |
| I468 Cardiac arrest due to other underlying condition |
| I469 Cardiac arrest, cause unspecified |
| I470 Re-entry ventricular arrhythmia |
| I471 Supraventricular tachycardia |
| I472 Ventricular tachycardia |
| I479 Paroxysmal tachycardia, unspecified |
| I480 Paroxysmal atrial fibrillation |
| I4811 Longstanding persistent atrial fibrillation |
| I4819 Other persistent atrial fibrillation |
| I4820 Chronic atrial fibrillation, unspecified |
| I4821 Permanent atrial fibrillation |
| I483 Typical atrial flutter |
| I484 Atypical atrial flutter |
| I4891 Unspecified atrial fibrillation |
| I4892 Unspecified atrial flutter |
| I4901 Ventricular fibrillation |
| I4902 Ventricular flutter |
| I491 Atrial premature depolarization |
| I492 Junctional premature depolarization |
| I493 Ventricular premature depolarization |
| I4940 Unspecified premature depolarization |
| I4949 Other premature depolarization |
| I495 Sick sinus syndrome |
| I498 Other specified cardiac arrhythmias |
| I499 Cardiac arrhythmia, unspecified |
| I501 Left ventricular failure, unspecified |
| I5020 Unspecified systolic (congestive) heart failure |
| I5021 Acute systolic (congestive) heart failure |
| I5022 Chronic systolic (congestive) heart failure |
| I5023 Acute on chronic systolic (congestive) heart failure |
| I5030 Unspecified diastolic (congestive) heart failure |
| I5031 Acute diastolic (congestive) heart failure |
| I5032 Chronic diastolic (congestive) heart failure |
| I5033 Acute on chronic diastolic (congestive) heart failure |
| I5040 Unspecified combined systolic (congestive) and diastolic (congestive) heart failure |
| I5041 Acute combined systolic (congestive) and diastolic (congestive) heart failure |
| I5042 Chronic combined systolic (congestive) and diastolic (congestive) heart failure |
| I5043 Acute on chronic combined systolic (congestive) and diastolic (congestive) heart failure |
| I50810 Right heart failure, unspecified |
| I50811 Acute right heart failure |
| I50812 Chronic right heart failure |
| I50813 Acute on chronic right heart failure |
| I50814 Right heart failure due to left heart failure |
| I5082 Biventricular heart failure |
| I5083 High output heart failure |
| I5084 End stage heart failure |
| I5089 Other heart failure |
| I509 Heart failure, unspecified |
| I6000 Nontraumatic subarachnoid hemorrhage from unspecified carotid siphon and bifurcation |
| I6001 Nontraumatic subarachnoid hemorrhage from right carotid siphon and bifurcation |
| I6002 Nontraumatic subarachnoid hemorrhage from left carotid siphon and bifurcation |
| I6010 Nontraumatic subarachnoid hemorrhage from unspecified middle cerebral artery |
| I6011 Nontraumatic subarachnoid hemorrhage from right middle cerebral artery |
| I6012 Nontraumatic subarachnoid hemorrhage from left middle cerebral artery |
| I602 Nontraumatic subarachnoid hemorrhage from anterior communicating artery |
| I6030 Nontraumatic subarachnoid hemorrhage from unspecified posterior communicating artery |
| I6031 Nontraumatic subarachnoid hemorrhage from right posterior communicating artery |
| I6032 Nontraumatic subarachnoid hemorrhage from left posterior communicating artery |
| I604 Nontraumatic subarachnoid hemorrhage from basilar artery |
| I6050 Nontraumatic subarachnoid hemorrhage from unspecified vertebral artery |
| I6051 Nontraumatic subarachnoid hemorrhage from right vertebral artery |
| I6052 Nontraumatic subarachnoid hemorrhage from left vertebral artery |
| I606 Nontraumatic subarachnoid hemorrhage from other intracranial arteries |
| I607 Nontraumatic subarachnoid hemorrhage from unspecified intracranial artery |
| I608 Other nontraumatic subarachnoid hemorrhage |
| I609 Nontraumatic subarachnoid hemorrhage, unspecified |
| I610 Nontraumatic intracerebral hemorrhage in hemisphere, subcortical |
| I611 Nontraumatic intracerebral hemorrhage in hemisphere, cortical |
| I612 Nontraumatic intracerebral hemorrhage in hemisphere, unspecified |
| I613 Nontraumatic intracerebral hemorrhage in brain stem |
| I614 Nontraumatic intracerebral hemorrhage in cerebellum |
| I615 Nontraumatic intracerebral hemorrhage, intraventricular |
| I616 Nontraumatic intracerebral hemorrhage, multiple localized |
| I618 Other nontraumatic intracerebral hemorrhage |
| I619 Nontraumatic intracerebral hemorrhage, unspecified |
| I6200 Nontraumatic subdural hemorrhage, unspecified |
| I6201 Nontraumatic acute subdural hemorrhage |
| I6202 Nontraumatic subacute subdural hemorrhage |
| I6203 Nontraumatic chronic subdural hemorrhage |
| I621 Nontraumatic extradural hemorrhage |
| I629 Nontraumatic intracranial hemorrhage, unspecified |
| I6300 Cerebral infarction due to thrombosis of unspecified precerebral artery |
| I63011 Cerebral infarction due to thrombosis of right vertebral artery |
| I63012 Cerebral infarction due to thrombosis of left vertebral artery |
| I63013 Cerebral infarction due to thrombosis of bilateral vertebral arteries |
| I63019 Cerebral infarction due to thrombosis of unspecified vertebral artery |
| I6302 Cerebral infarction due to thrombosis of basilar artery |
| I63031 Cerebral infarction due to thrombosis of right carotid artery |
| I63032 Cerebral infarction due to thrombosis of left carotid artery |
| I63033 Cerebral infarction due to thrombosis of bilateral carotid arteries |
| I63039 Cerebral infarction due to thrombosis of unspecified carotid artery |
| I6309 Cerebral infarction due to thrombosis of other precerebral artery |
| I6310 Cerebral infarction due to embolism of unspecified precerebral artery |
| I63111 Cerebral infarction due to embolism of right vertebral artery |
| I63112 Cerebral infarction due to embolism of left vertebral artery |
| I63113 Cerebral infarction due to embolism of bilateral vertebral arteries |
| I63119 Cerebral infarction due to embolism of unspecified vertebral artery |
| I6312 Cerebral infarction due to embolism of basilar artery |
| I63131 Cerebral infarction due to embolism of right carotid artery |
| I63132 Cerebral infarction due to embolism of left carotid artery |
| I63133 Cerebral infarction due to embolism of bilateral carotid arteries |
| I63139 Cerebral infarction due to embolism of unspecified carotid artery |
| I6319 Cerebral infarction due to embolism of other precerebral artery |
| I6320 Cerebral infarction due to unspecified occlusion or stenosis of unspecified precerebral arteries |
| I63211 Cerebral infarction due to unspecified occlusion or stenosis of right vertebral artery |
| I63212 Cerebral infarction due to unspecified occlusion or stenosis of left vertebral artery |
| I63213 Cerebral infarction due to unspecified occlusion or stenosis of bilateral vertebral arteries |
| I63219 Cerebral infarction due to unspecified occlusion or stenosis of unspecified vertebral artery |
| I6322 Cerebral infarction due to unspecified occlusion or stenosis of basilar artery |
| I63231 Cerebral infarction due to unspecified occlusion or stenosis of right carotid arteries |
| I63232 Cerebral infarction due to unspecified occlusion or stenosis of left carotid arteries |
| I63233 Cerebral infarction due to unspecified occlusion or stenosis of bilateral carotid arteries |
| I63239 Cerebral infarction due to unspecified occlusion or stenosis of unspecified carotid artery |
| I6329 Cerebral infarction due to unspecified occlusion or stenosis of other precerebral arteries |
| I6330 Cerebral infarction due to thrombosis of unspecified cerebral artery |
| I63311 Cerebral infarction due to thrombosis of right middle cerebral artery |
| I63312 Cerebral infarction due to thrombosis of left middle cerebral artery |
| I63313 Cerebral infarction due to thrombosis of bilateral middle cerebral arteries |
| I63319 Cerebral infarction due to thrombosis of unspecified middle cerebral artery |
| I63321 Cerebral infarction due to thrombosis of right anterior cerebral artery |
| I63322 Cerebral infarction due to thrombosis of left anterior cerebral artery |
| I63323 Cerebral infarction due to thrombosis of bilateral anterior cerebral arteries |
| I63329 Cerebral infarction due to thrombosis of unspecified anterior cerebral artery |
| I63331 Cerebral infarction due to thrombosis of right posterior cerebral artery |
| I63332 Cerebral infarction due to thrombosis of left posterior cerebral artery |
| I63333 Cerebral infarction due to thrombosis of bilateral posterior cerebral arteries |
| I63339 Cerebral infarction due to thrombosis of unspecified posterior cerebral artery |
| I63341 Cerebral infarction due to thrombosis of right cerebellar artery |
| I63342 Cerebral infarction due to thrombosis of left cerebellar artery |
| I63343 Cerebral infarction due to thrombosis of bilateral cerebellar arteries |
| I63349 Cerebral infarction due to thrombosis of unspecified cerebellar artery |
| I6339 Cerebral infarction due to thrombosis of other cerebral artery |
| I6340 Cerebral infarction due to embolism of unspecified cerebral artery |
| I63411 Cerebral infarction due to embolism of right middle cerebral artery |
| I63412 Cerebral infarction due to embolism of left middle cerebral artery |
| I63413 Cerebral infarction due to embolism of bilateral middle cerebral arteries |
| I63419 Cerebral infarction due to embolism of unspecified middle cerebral artery |
| I63421 Cerebral infarction due to embolism of right anterior cerebral artery |
| I63422 Cerebral infarction due to embolism of left anterior cerebral artery |
| I63423 Cerebral infarction due to embolism of bilateral anterior cerebral arteries |
| I63429 Cerebral infarction due to embolism of unspecified anterior cerebral artery |
| I63431 Cerebral infarction due to embolism of right posterior cerebral artery |
| I63432 Cerebral infarction due to embolism of left posterior cerebral artery |
| I63433 Cerebral infarction due to embolism of bilateral posterior cerebral arteries |
| I63439 Cerebral infarction due to embolism of unspecified posterior cerebral artery |
| I63441 Cerebral infarction due to embolism of right cerebellar artery |
| I63442 Cerebral infarction due to embolism of left cerebellar artery |
| I63443 Cerebral infarction due to embolism of bilateral cerebellar arteries |
| I63449 Cerebral infarction due to embolism of unspecified cerebellar artery |
| I6349 Cerebral infarction due to embolism of other cerebral artery |
| I6350 Cerebral infarction due to unspecified occlusion or stenosis of unspecified cerebral artery |
| I63511 Cerebral infarction due to unspecified occlusion or stenosis of right middle cerebral artery |
| I63512 Cerebral infarction due to unspecified occlusion or stenosis of left middle cerebral artery |
| I63513 Cerebral infarction due to unspecified occlusion or stenosis of bilateral middle cerebral arteries |
| I63519 Cerebral infarction due to unspecified occlusion or stenosis of unspecified middle cerebral artery |
| I63521 Cerebral infarction due to unspecified occlusion or stenosis of right anterior cerebral artery |
| I63522 Cerebral infarction due to unspecified occlusion or stenosis of left anterior cerebral artery |
| I63523 Cerebral infarction due to unspecified occlusion or stenosis of bilateral anterior cerebral arteries |
| I63529 Cerebral infarction due to unspecified occlusion or stenosis of unspecified anterior cerebral artery |
| I63531 Cerebral infarction due to unspecified occlusion or stenosis of right posterior cerebral artery |
| I63532 Cerebral infarction due to unspecified occlusion or stenosis of left posterior cerebral artery |
| I63533 Cerebral infarction due to unspecified occlusion or stenosis of bilateral posterior cerebral arteries |
| I63539 Cerebral infarction due to unspecified occlusion or stenosis of unspecified posterior cerebral artery |
| I63541 Cerebral infarction due to unspecified occlusion or stenosis of right cerebellar artery |
| I63542 Cerebral infarction due to unspecified occlusion or stenosis of left cerebellar artery |
| I63543 Cerebral infarction due to unspecified occlusion or stenosis of bilateral cerebellar arteries |
| I63549 Cerebral infarction due to unspecified occlusion or stenosis of unspecified cerebellar artery |
| I6359 Cerebral infarction due to unspecified occlusion or stenosis of other cerebral artery |
| I636 Cerebral infarction due to cerebral venous thrombosis, nonpyogenic |
| I6381 Other cerebral infarction due to occlusion or stenosis of small artery |
| I6389 Other cerebral infarction |
| I639 Cerebral infarction, unspecified |
| I6783 Posterior reversible encephalopathy syndrome |
| I67841 Reversible cerebrovascular vasoconstriction syndrome |
| I674 Hypertensive encephalopathy |
| I675 Moyamoya disease |
| I679 Cerebrovascular disease, unspecified |
| J4540 Moderate persistent asthma, uncomplicated |
| J4541 Moderate persistent asthma with (acute) exacerbation |
| J4542 Moderate persistent asthma with status asthmaticus |
| J4550 Severe persistent asthma, uncomplicated |
| J4551 Severe persistent asthma with (acute) exacerbation |
| J4552 Severe persistent asthma with status asthmaticus |
| J810 Acute pulmonary edema |
| K739 Chronic hepatitis, unspecified |
| K754 Autoimmune hepatitis |
| K743 Primary biliary cirrhosis |
| K8501 Idiopathic acute pancreatitis with uninfected necrosis |
| K8502 Idiopathic acute pancreatitis with infected necrosis |
| K8510 Biliary acute pancreatitis without necrosis or infection |
| K8511 Biliary acute pancreatitis with uninfected necrosis |
| K8512 Biliary acute pancreatitis with infected necrosis |
| K8520 Alcohol induced acute pancreatitis without necrosis or infection |
| K8521 Alcohol induced acute pancreatitis with uninfected necrosis |
| K8522 Alcohol induced acute pancreatitis with infected necrosis |
| K8530 Drug induced acute pancreatitis without necrosis or infection |
| K8531 Drug induced acute pancreatitis with uninfected necrosis |
| K8532 Drug induced acute pancreatitis with infected necrosis |
| K8580 Other acute pancreatitis without necrosis or infection |
| K8581 Other acute pancreatitis with uninfected necrosis |
| K8582 Other acute pancreatitis with infected necrosis |
| K8590 Acute pancreatitis without necrosis or infection, unspecified |
| K8591 Acute pancreatitis with uninfected necrosis, unspecified |
| K8592 Acute pancreatitis with infected necrosis, unspecified |
| K860 Alcohol-induced chronic pancreatitis |
| K861 Other chronic pancreatitis |
| M0560 Rheumatoid arthritis of unspecified site with involvement of other organs and systems |
| M05611 Rheumatoid arthritis of right shoulder with involvement of other organs and systems |
| M05612 Rheumatoid arthritis of left shoulder with involvement of other organs and systems |
| M05619 Rheumatoid arthritis of unspecified shoulder with involvement of other organs and systems |
| M05621 Rheumatoid arthritis of right elbow with involvement of other organs and systems |
| M05622 Rheumatoid arthritis of left elbow with involvement of other organs and systems |
| M05629 Rheumatoid arthritis of unspecified elbow with involvement of other organs and systems |
| M05631 Rheumatoid arthritis of right wrist with involvement of other organs and systems |
| M05632 Rheumatoid arthritis of left wrist with involvement of other organs and systems |
| M05639 Rheumatoid arthritis of unspecified wrist with involvement of other organs and systems |
| M05641 Rheumatoid arthritis of right hand with involvement of other organs and systems |
| M05642 Rheumatoid arthritis of left hand with involvement of other organs and systems |
| M05649 Rheumatoid arthritis of unspecified hand with involvement of other organs and systems |
| M05651 Rheumatoid arthritis of right hip with involvement of other organs and systems |
| M05652 Rheumatoid arthritis of left hip with involvement of other organs and systems |
| M05659 Rheumatoid arthritis of unspecified hip with involvement of other organs and systems |
| M05661 Rheumatoid arthritis of right knee with involvement of other organs and systems |
| M05662 Rheumatoid arthritis of left knee with involvement of other organs and systems |
| M05669 Rheumatoid arthritis of unspecified knee with involvement of other organs and systems |
| M05671 Rheumatoid arthritis of right ankle and foot with involvement of other organs and systems |
| M05672 Rheumatoid arthritis of left ankle and foot with involvement of other organs and systems |
| M05679 Rheumatoid arthritis of unspecified ankle and foot with involvement of other organs and systems |
| M0569 Rheumatoid arthritis of multiple sites with involvement of other organs and systems |
| M0570 Rheumatoid arthritis with rheumatoid factor of unspecified site without organ or systems involvement |
| M05711 Rheumatoid arthritis with rheumatoid factor of right shoulder without organ or systems involvement |
| M05712 Rheumatoid arthritis with rheumatoid factor of left shoulder without organ or systems involvement |
| M05719 Rheumatoid arthritis with rheumatoid factor of unspecified shoulder without organ or systems involvement |
| M05721 Rheumatoid arthritis with rheumatoid factor of right elbow without organ or systems involvement |
| M05722 Rheumatoid arthritis with rheumatoid factor of left elbow without organ or systems involvement |
| M05729 Rheumatoid arthritis with rheumatoid factor of unspecified elbow without organ or systems involvement |
| M05731 Rheumatoid arthritis with rheumatoid factor of right wrist without organ or systems involvement |
| M05732 Rheumatoid arthritis with rheumatoid factor of left wrist without organ or systems involvement |
| M05739 Rheumatoid arthritis with rheumatoid factor of unspecified wrist without organ or systems involvement |
| M05741 Rheumatoid arthritis with rheumatoid factor of right hand without organ or systems involvement |
| M05742 Rheumatoid arthritis with rheumatoid factor of left hand without organ or systems involvement |
| M05749 Rheumatoid arthritis with rheumatoid factor of unspecified hand without organ or systems involvement |
| M05751 Rheumatoid arthritis with rheumatoid factor of right hip without organ or systems involvement |
| M05752 Rheumatoid arthritis with rheumatoid factor of left hip without organ or systems involvement |
| M05759 Rheumatoid arthritis with rheumatoid factor of unspecified hip without organ or systems involvement |
| M05761 Rheumatoid arthritis with rheumatoid factor of right knee without organ or systems involvement |
| M05762 Rheumatoid arthritis with rheumatoid factor of left knee without organ or systems involvement |
| M05769 Rheumatoid arthritis with rheumatoid factor of unspecified knee without organ or systems involvement |
| M05771 Rheumatoid arthritis with rheumatoid factor of right ankle and foot without organ or systems involvement |
| M05772 Rheumatoid arthritis with rheumatoid factor of left ankle and foot without organ or systems involvement |
| M05779 Rheumatoid arthritis with rheumatoid factor of unspecified ankle and foot without organ or systems involvement |
| M0579 Rheumatoid arthritis with rheumatoid factor of multiple sites without organ or systems involvement |
| M057A Rheumatoid arthritis with rheumatoid factor of other specified site without organ or systems involvement |
| M0580 Other rheumatoid arthritis with rheumatoid factor of unspecified site |
| M05811 Other rheumatoid arthritis with rheumatoid factor of right shoulder |
| M05812 Other rheumatoid arthritis with rheumatoid factor of left shoulder |
| M05819 Other rheumatoid arthritis with rheumatoid factor of unspecified shoulder |
| M05821 Other rheumatoid arthritis with rheumatoid factor of right elbow |
| M05822 Other rheumatoid arthritis with rheumatoid factor of left elbow |
| M05829 Other rheumatoid arthritis with rheumatoid factor of unspecified elbow |
| M05831 Other rheumatoid arthritis with rheumatoid factor of right wrist |
| M05832 Other rheumatoid arthritis with rheumatoid factor of left wrist |
| M05839 Other rheumatoid arthritis with rheumatoid factor of unspecified wrist |
| M05841 Other rheumatoid arthritis with rheumatoid factor of right hand |
| M05842 Other rheumatoid arthritis with rheumatoid factor of left hand |
| M05849 Other rheumatoid arthritis with rheumatoid factor of unspecified hand |
| M05851 Other rheumatoid arthritis with rheumatoid factor of right hip |
| M05852 Other rheumatoid arthritis with rheumatoid factor of left hip |
| M05859 Other rheumatoid arthritis with rheumatoid factor of unspecified hip |
| M05861 Other rheumatoid arthritis with rheumatoid factor of right knee |
| M05862 Other rheumatoid arthritis with rheumatoid factor of left knee |
| M05869 Other rheumatoid arthritis with rheumatoid factor of unspecified knee |
| M05871 Other rheumatoid arthritis with rheumatoid factor of right ankle and foot |
| M05872 Other rheumatoid arthritis with rheumatoid factor of left ankle and foot |
| M05879 Other rheumatoid arthritis with rheumatoid factor of unspecified ankle and foot |
| M0589 Other rheumatoid arthritis with rheumatoid factor of multiple sites |
| M058A Other rheumatoid arthritis with rheumatoid factor of other specified site |
| M059 Rheumatoid arthritis with rheumatoid factor, unspecified |
| M0600 Rheumatoid arthritis without rheumatoid factor, unspecified site |
| M06011 Rheumatoid arthritis without rheumatoid factor, right shoulder |
| M06012 Rheumatoid arthritis without rheumatoid factor, left shoulder |
| M06019 Rheumatoid arthritis without rheumatoid factor, unspecified shoulder |
| M06021 Rheumatoid arthritis without rheumatoid factor, right elbow |
| M06022 Rheumatoid arthritis without rheumatoid factor, left elbow |
| M06029 Rheumatoid arthritis without rheumatoid factor, unspecified elbow |
| M06031 Rheumatoid arthritis without rheumatoid factor, right wrist |
| M06032 Rheumatoid arthritis without rheumatoid factor, left wrist |
| M06039 Rheumatoid arthritis without rheumatoid factor, unspecified wrist |
| M06041 Rheumatoid arthritis without rheumatoid factor, right hand |
| M06042 Rheumatoid arthritis without rheumatoid factor, left hand |
| M06049 Rheumatoid arthritis without rheumatoid factor, unspecified hand |
| M06051 Rheumatoid arthritis without rheumatoid factor, right hip |
| M06052 Rheumatoid arthritis without rheumatoid factor, left hip |
| M06059 Rheumatoid arthritis without rheumatoid factor, unspecified hip |
| M06061 Rheumatoid arthritis without rheumatoid factor, right knee |
| M06062 Rheumatoid arthritis without rheumatoid factor, left knee |
| M06069 Rheumatoid arthritis without rheumatoid factor, unspecified knee |
| M06071 Rheumatoid arthritis without rheumatoid factor, right ankle and foot |
| M06072 Rheumatoid arthritis without rheumatoid factor, left ankle and foot |
| M06079 Rheumatoid arthritis without rheumatoid factor, unspecified ankle and foot |
| M0608 Rheumatoid arthritis without rheumatoid factor, vertebrae |
| M0609 Rheumatoid arthritis without rheumatoid factor, multiple sites |
| M060A Rheumatoid arthritis without rheumatoid factor, other specified site |
| M0680 Other specified rheumatoid arthritis, unspecified site |
| M06811 Other specified rheumatoid arthritis, right shoulder |
| M06812 Other specified rheumatoid arthritis, left shoulder |
| M06819 Other specified rheumatoid arthritis, unspecified shoulder |
| M06821 Other specified rheumatoid arthritis, right elbow |
| M06822 Other specified rheumatoid arthritis, left elbow |
| M06829 Other specified rheumatoid arthritis, unspecified elbow |
| M06831 Other specified rheumatoid arthritis, right wrist |
| M06832 Other specified rheumatoid arthritis, left wrist |
| M06839 Other specified rheumatoid arthritis, unspecified wrist |
| M06841 Other specified rheumatoid arthritis, right hand |
| M06842 Other specified rheumatoid arthritis, left hand |
| M06849 Other specified rheumatoid arthritis, unspecified hand |
| M06851 Other specified rheumatoid arthritis, right hip |
| M06852 Other specified rheumatoid arthritis, left hip |
| M06859 Other specified rheumatoid arthritis, unspecified hip |
| M06861 Other specified rheumatoid arthritis, right knee |
| M06862 Other specified rheumatoid arthritis, left knee |
| M06869 Other specified rheumatoid arthritis, unspecified knee |
| M06871 Other specified rheumatoid arthritis, right ankle and foot |
| M06872 Other specified rheumatoid arthritis, left ankle and foot |
| M06879 Other specified rheumatoid arthritis, unspecified ankle and foot |
| M0688 Other specified rheumatoid arthritis, vertebrae |
| M0689 Other specified rheumatoid arthritis, multiple sites |
| M068A Other specified rheumatoid arthritis, other specified site |
| N170 Acute kidney failure with tubular necrosis |
| N171 Acute kidney failure with acute cortical necrosis |
| N172 Acute kidney failure with medullary necrosis |
| N178 Other acute kidney failure |
| N179 Acute kidney failure, unspecified |
| N186 End stage renal disease |
| N189 Chronic kidney disease, unspecified |
| N19 Unspecified kidney failure |
| O131 Gestational [pregnancy-induced] hypertension without significant proteinuria, first trimester |
| O132 Gestational [pregnancy-induced] hypertension without significant proteinuria, second trimester |
| O133 Gestational [pregnancy-induced] hypertension without significant proteinuria, third trimester |
| O134 Gestational [pregnancy-induced] hypertension without significant proteinuria, complicating childbirth |
| O135 Gestational [pregnancy-induced] hypertension without significant proteinuria, complicating the puerperium |
| O139 Gestational [pregnancy-induced] hypertension without significant proteinuria, unspecified trimester |
| O1400 Mild to moderate pre-eclampsia, unspecified trimester |
| O161 Unspecified maternal hypertension, first trimester |
| O162 Unspecified maternal hypertension, second trimester |
| O163 Unspecified maternal hypertension, third trimester |
| O164 Unspecified maternal hypertension, complicating childbirth |
| O165 Unspecified maternal hypertension, complicating the puerperium |
| O169 Unspecified maternal hypertension, unspecified trimester |
| O24410 Gestational diabetes mellitus in pregnancy, diet controlled |
| O24414 Gestational diabetes mellitus in pregnancy, insulin controlled |
| O24415 Gestational diabetes mellitus in pregnancy, controlled by oral hypoglycemic drugs |
| O24419 Gestational diabetes mellitus in pregnancy, unspecified control |
| O24420 Gestational diabetes mellitus in childbirth, diet controlled |
| O24424 Gestational diabetes mellitus in childbirth, insulin controlled |
| O24425 Gestational diabetes mellitus in childbirth, controlled by oral hypoglycemic drugs |
| O24429 Gestational diabetes mellitus in childbirth, unspecified control |
| O24430 Gestational diabetes mellitus in the puerperium, diet controlled |
| O24434 Gestational diabetes mellitus in the puerperium, insulin controlled |
| O24435 Gestational diabetes mellitus in puerperium, controlled by oral hypoglycemic drugs |
| O24439 Gestational diabetes mellitus in the puerperium, unspecified control |
| O24911 Unspecified diabetes mellitus in pregnancy, first trimester |
| O24912 Unspecified diabetes mellitus in pregnancy, second trimester |
| O24913 Unspecified diabetes mellitus in pregnancy, third trimester |
| O24919 Unspecified diabetes mellitus in pregnancy, unspecified trimester |
| O2492 Unspecified diabetes mellitus in childbirth |
| O2493 Unspecified diabetes mellitus in the puerperium |
| O26831 Pregnancy related renal disease, first trimester |
| O26832 Pregnancy related renal disease, second trimester |
| O26833 Pregnancy related renal disease, third trimester |
| O26839 Pregnancy related renal disease, unspecified trimester |
| O43211 Placenta accreta, first trimester |
| O43212 Placenta accreta, second trimester |
| O43213 Placenta accreta, third trimester |
| O43219 Placenta accreta, unspecified trimester |
| O43221 Placenta increta, first trimester |
| O43222 Placenta increta, second trimester |
| O43223 Placenta increta, third trimester |
| O43229 Placenta increta, unspecified trimester |
| O43231 Placenta percreta, first trimester |
| O43232 Placenta percreta, second trimester |
| O43233 Placenta percreta, third trimester |
| O43239 Placenta percreta, unspecified trimester |
| O46001 Antepartum hemorrhage with coagulation defect, unspecified, first trimester |
| O46002 Antepartum hemorrhage with coagulation defect, unspecified, second trimester |
| O46003 Antepartum hemorrhage with coagulation defect, unspecified, third trimester |
| O46009 Antepartum hemorrhage with coagulation defect, unspecified, unspecified trimester |
| O46011 Antepartum hemorrhage with afibrinogenemia, first trimester |
| O46012 Antepartum hemorrhage with afibrinogenemia, second trimester |
| O46013 Antepartum hemorrhage with afibrinogenemia, third trimester |
| O46019 Antepartum hemorrhage with afibrinogenemia, unspecified trimester |
| O46021 Antepartum hemorrhage with disseminated intravascular coagulation, first trimester |
| O46022 Antepartum hemorrhage with disseminated intravascular coagulation, second trimester |
| O46023 Antepartum hemorrhage with disseminated intravascular coagulation, third trimester |
| O46029 Antepartum hemorrhage with disseminated intravascular coagulation, unspecified trimester |
| O46091 Antepartum hemorrhage with other coagulation defect, first trimester |
| O46092 Antepartum hemorrhage with other coagulation defect, second trimester |
| O46093 Antepartum hemorrhage with other coagulation defect, third trimester |
| O46099 Antepartum hemorrhage with other coagulation defect, unspecified trimester |
| O468X1 Other antepartum hemorrhage, first trimester |
| O468X2 Other antepartum hemorrhage, second trimester |
| O468X3 Other antepartum hemorrhage, third trimester |
| O468X9 Other antepartum hemorrhage, unspecified trimester |
| O4690 Antepartum hemorrhage, unspecified, unspecified trimester |
| O4691 Antepartum hemorrhage, unspecified, first trimester |
| O4692 Antepartum hemorrhage, unspecified, second trimester |
| O4693 Antepartum hemorrhage, unspecified, third trimester |
| O7100 Rupture of uterus before onset of labor, unspecified trimester |
| O7102 Rupture of uterus before onset of labor, second trimester |
| O7103 Rupture of uterus before onset of labor, third trimester |
| O711 Rupture of uterus during labor |
| O712 Postpartum inversion of uterus |
| O713 Obstetric laceration of cervix |
| O721 Other immediate postpartum hemorrhage |
| O722 Delayed and secondary postpartum hemorrhage |
| O723 Postpartum coagulation defects |
| O88011 Air embolism in pregnancy, first trimester |
| O88012 Air embolism in pregnancy, second trimester |
| O88013 Air embolism in pregnancy, third trimester |
| O88019 Air embolism in pregnancy, unspecified trimester |
| O8802 Air embolism in childbirth |
| O8803 Air embolism in the puerperium |
| O88111 Amniotic fluid embolism in pregnancy, first trimester |
| O88112 Amniotic fluid embolism in pregnancy, second trimester |
| O88113 Amniotic fluid embolism in pregnancy, third trimester |
| O88119 Amniotic fluid embolism in pregnancy, unspecified trimester |
| O8812 Amniotic fluid embolism in childbirth |
| O8813 Amniotic fluid embolism in the puerperium |
| Z8616 Personal history of COVID-19 |
| J1282 Pneumonia due to coronavirus disease 2019 |
| O42011 Preterm premature rupture of membranes, onset of labor within 24 hours of rupture, first trimester |
| O42012 Preterm premature rupture of membranes, onset of labor within 24 hours of rupture, second trimester |
| O42013 Preterm premature rupture of membranes, onset of labor within 24 hours of rupture, third trimester |
| O42019 Preterm premature rupture of membranes, onset of labor within 24 hours of rupture, unspecified trimester |
| O42111 Preterm premature rupture of membranes, onset of labor more than 24 hours following rupture, first trimester |
| O42112 Preterm premature rupture of membranes, onset of labor more than 24 hours following rupture, second trimester |
| O42113 Preterm premature rupture of membranes, onset of labor more than 24 hours following rupture, third trimester |
| O42119 Preterm premature rupture of membranes, onset of labor more than 24 hours following rupture, unspecified trimester |
| O42911 Preterm premature rupture of membranes, unspecified as to length of time between rupture and onset of labor, first trimester |
| O42912 Preterm premature rupture of membranes, unspecified as to length of time between rupture and onset of labor, second trimester |
| O42913 Preterm premature rupture of membranes, unspecified as to length of time between rupture and onset of labor, third trimester |
| O42919 Preterm premature rupture of membranes, unspecified as to length of time between rupture and onset of labor, unspecified trimester |
| O45001 Premature separation of placenta with coagulation defect, unspecified, first trimester |
| O45002 Premature separation of placenta with coagulation defect, unspecified, second trimester |
| O45003 Premature separation of placenta with coagulation defect, unspecified, third trimester |
| O45009 Premature separation of placenta with coagulation defect, unspecified, unspecified trimester |
| O45011 Premature separation of placenta with afibrinogenemia, first trimester |
| O45012 Premature separation of placenta with afibrinogenemia, second trimester |
| O45013 Premature separation of placenta with afibrinogenemia, third trimester |
| O45019 Premature separation of placenta with afibrinogenemia, unspecified trimester |
| O45021 Premature separation of placenta with disseminated intravascular coagulation, first trimester |
| O45022 Premature separation of placenta with disseminated intravascular coagulation, second trimester |
| O45023 Premature separation of placenta with disseminated intravascular coagulation, third trimester |
| O45029 Premature separation of placenta with disseminated intravascular coagulation, unspecified trimester |
| O45091 Premature separation of placenta with other coagulation defect, first trimester |
| O45092 Premature separation of placenta with other coagulation defect, second trimester |
| O45093 Premature separation of placenta with other coagulation defect, third trimester |
| O45099 Premature separation of placenta with other coagulation defect, unspecified trimester |
| O458X1 Other premature separation of placenta, first trimester |
| O458X2 Other premature separation of placenta, second trimester |
| O458X3 Other premature separation of placenta, third trimester |
| O458X9 Other premature separation of placenta, unspecified trimester |
| O4590 Premature separation of placenta, unspecified, unspecified trimester |
| O4591 Premature separation of placenta, unspecified, first trimester |
| O4592 Premature separation of placenta, unspecified, second trimester |
| O4593 Premature separation of placenta, unspecified, third trimester |
| O694XX0 Labor and delivery complicated by vasa previa, not applicable or unspecified |
| O694XX1 Labor and delivery complicated by vasa previa, fetus 1 |
| O694XX2 Labor and delivery complicated by vasa previa, fetus 2 |
| O694XX3 Labor and delivery complicated by vasa previa, fetus 3 |
| O694XX4 Labor and delivery complicated by vasa previa, fetus 4 |
| O694XX5 Labor and delivery complicated by vasa previa, fetus 5 |
| O694XX9 Labor and delivery complicated by vasa previa, other fetus |
| O4400 Complete placenta previa NOS or without hemorrhage, unspecified trimester |
| O4401 Complete placenta previa NOS or without hemorrhage, first trimester |
| O4402 Complete placenta previa NOS or without hemorrhage, second trimester |
| O4403 Complete placenta previa NOS or without hemorrhage, third trimester |
| O4410 Complete placenta previa with hemorrhage, unspecified trimester |
| O4411 Complete placenta previa with hemorrhage, first trimester |
| O4412 Complete placenta previa with hemorrhage, second trimester |
| O4413 Complete placenta previa with hemorrhage, third trimester |
| O4420 Partial placenta previa NOS or without hemorrhage, unspecified trimester |
| O4421 Partial placenta previa NOS or without hemorrhage, first trimester |
| O4422 Partial placenta previa NOS or without hemorrhage, second trimester |
| O4423 Partial placenta previa NOS or without hemorrhage, third trimester |
| O4430 Partial placenta previa with hemorrhage, unspecified trimester |
| O4431 Partial placenta previa with hemorrhage, first trimester |
| O4432 Partial placenta previa with hemorrhage, second trimester |
| O4433 Partial placenta previa with hemorrhage, third trimester |
| O30001 Twin pregnancy, unspecified number of placenta and unspecified number of amniotic sacs, first trimester |
| O30002 Twin pregnancy, unspecified number of placenta and unspecified number of amniotic sacs, second trimester |
| O30003 Twin pregnancy, unspecified number of placenta and unspecified number of amniotic sacs, third trimester |
| O30009 Twin pregnancy, unspecified number of placenta and unspecified number of amniotic sacs, unspecified trimester |
| O30011 Twin pregnancy, monochorionic/monoamniotic, first trimester |
| O30012 Twin pregnancy, monochorionic/monoamniotic, second trimester |
| O30013 Twin pregnancy, monochorionic/monoamniotic, third trimester |
| O30019 Twin pregnancy, monochorionic/monoamniotic, unspecified trimester |
| O30021 Conjoined twin pregnancy, first trimester |
| O30022 Conjoined twin pregnancy, second trimester |
| O30023 Conjoined twin pregnancy, third trimester |
| O30029 Conjoined twin pregnancy, unspecified trimester |
| O30031 Twin pregnancy, monochorionic/diamniotic, first trimester |
| O30032 Twin pregnancy, monochorionic/diamniotic, second trimester |
| O30033 Twin pregnancy, monochorionic/diamniotic, third trimester |
| O30039 Twin pregnancy, monochorionic/diamniotic, unspecified trimester |
| O30041 Twin pregnancy, dichorionic/diamniotic, first trimester |
| O30042 Twin pregnancy, dichorionic/diamniotic, second trimester |
| O30043 Twin pregnancy, dichorionic/diamniotic, third trimester |
| O30049 Twin pregnancy, dichorionic/diamniotic, unspecified trimester |
| O30091 Twin pregnancy, unable to determine number of placenta and number of amniotic sacs, first trimester |
| O30092 Twin pregnancy, unable to determine number of placenta and number of amniotic sacs, second trimester |
| O30093 Twin pregnancy, unable to determine number of placenta and number of amniotic sacs, third trimester |
| O30099 Twin pregnancy, unable to determine number of placenta and number of amniotic sacs, unspecified trimester |
| O30101 Triplet pregnancy, unspecified number of placenta and unspecified number of amniotic sacs, first trimester |
| O30102 Triplet pregnancy, unspecified number of placenta and unspecified number of amniotic sacs, second trimester |
| O30103 Triplet pregnancy, unspecified number of placenta and unspecified number of amniotic sacs, third trimester |
| O30109 Triplet pregnancy, unspecified number of placenta and unspecified number of amniotic sacs, unspecified trimester |
| O30111 Triplet pregnancy with two or more monochorionic fetuses, first trimester |
| O30112 Triplet pregnancy with two or more monochorionic fetuses, second trimester |
| O30113 Triplet pregnancy with two or more monochorionic fetuses, third trimester |
| O30119 Triplet pregnancy with two or more monochorionic fetuses, unspecified trimester |
| O30121 Triplet pregnancy with two or more monoamniotic fetuses, first trimester |
| O30122 Triplet pregnancy with two or more monoamniotic fetuses, second trimester |
| O30123 Triplet pregnancy with two or more monoamniotic fetuses, third trimester |
| O30129 Triplet pregnancy with two or more monoamniotic fetuses, unspecified trimester |
| O30131 Triplet pregnancy, trichorionic/triamniotic, first trimester |
| O30132 Triplet pregnancy, trichorionic/triamniotic, second trimester |
| O30133 Triplet pregnancy, trichorionic/triamniotic, third trimester |
| O30139 Triplet pregnancy, trichorionic/triamniotic, unspecified trimester |
| O30191 Triplet pregnancy, unable to determine number of placenta and number of amniotic sacs, first trimester |
| O30192 Triplet pregnancy, unable to determine number of placenta and number of amniotic sacs, second trimester |
| O30193 Triplet pregnancy, unable to determine number of placenta and number of amniotic sacs, third trimester |
| O30199 Triplet pregnancy, unable to determine number of placenta and number of amniotic sacs, unspecified trimester |
| O30201 Quadruplet pregnancy, unspecified number of placenta and unspecified number of amniotic sacs, first trimester |
| O30202 Quadruplet pregnancy, unspecified number of placenta and unspecified number of amniotic sacs, second trimester |
| O30203 Quadruplet pregnancy, unspecified number of placenta and unspecified number of amniotic sacs, third trimester |
| O30209 Quadruplet pregnancy, unspecified number of placenta and unspecified number of amniotic sacs, unspecified trimester |
| O30211 Quadruplet pregnancy with two or more monochorionic fetuses, first trimester |
| O30212 Quadruplet pregnancy with two or more monochorionic fetuses, second trimester |
| O30213 Quadruplet pregnancy with two or more monochorionic fetuses, third trimester |
| O30219 Quadruplet pregnancy with two or more monochorionic fetuses, unspecified trimester |
| O30221 Quadruplet pregnancy with two or more monoamniotic fetuses, first trimester |
| O30222 Quadruplet pregnancy with two or more monoamniotic fetuses, second trimester |
| O30223 Quadruplet pregnancy with two or more monoamniotic fetuses, third trimester |
| O30229 Quadruplet pregnancy with two or more monoamniotic fetuses, unspecified trimester |
| O30231 Quadruplet pregnancy, quadrachorionic/quadra-amniotic, first trimester |
| O30232 Quadruplet pregnancy, quadrachorionic/quadra-amniotic, second trimester |
| O30233 Quadruplet pregnancy, quadrachorionic/quadra-amniotic, third trimester |
| O30239 Quadruplet pregnancy, quadrachorionic/quadra-amniotic, unspecified trimester |
| O30291 Quadruplet pregnancy, unable to determine number of placenta and number of amniotic sacs, first trimester |
| O30292 Quadruplet pregnancy, unable to determine number of placenta and number of amniotic sacs, second trimester |
| O30293 Quadruplet pregnancy, unable to determine number of placenta and number of amniotic sacs, third trimester |
| O30299 Quadruplet pregnancy, unable to determine number of placenta and number of amniotic sacs, unspecified trimester |
| O30801 Other specified multiple gestation, unspecified number of placenta and unspecified number of amniotic sacs, first trimester |
| O30802 Other specified multiple gestation, unspecified number of placenta and unspecified number of amniotic sacs, second trimester |
| O30803 Other specified multiple gestation, unspecified number of placenta and unspecified number of amniotic sacs, third trimester |
| O30809 Other specified multiple gestation, unspecified number of placenta and unspecified number of amniotic sacs, unspecified trimester |
| O30811 Other specified multiple gestation with two or more monochorionic fetuses, first trimester |
| O30812 Other specified multiple gestation with two or more monochorionic fetuses, second trimester |
| O30813 Other specified multiple gestation with two or more monochorionic fetuses, third trimester |
| O30819 Other specified multiple gestation with two or more monochorionic fetuses, unspecified trimester |
| O30821 Other specified multiple gestation with two or more monoamniotic fetuses, first trimester |
| O30822 Other specified multiple gestation with two or more monoamniotic fetuses, second trimester |
| O30823 Other specified multiple gestation with two or more monoamniotic fetuses, third trimester |
| O30829 Other specified multiple gestation with two or more monoamniotic fetuses, unspecified trimester |
| O30831 Other specified multiple gestation, number of chorions and amnions are both equal to the number of fetuses, first trimester |
| O30832 Other specified multiple gestation, number of chorions and amnions are both equal to the number of fetuses, second trimester |
| O30833 Other specified multiple gestation, number of chorions and amnions are both equal to the number of fetuses, third trimester |
| O30839 Other specified multiple gestation, number of chorions and amnions are both equal to the number of fetuses, unspecified trimester |
| O30891 Other specified multiple gestation, unable to determine number of placenta and number of amniotic sacs, first trimester |
| O30892 Other specified multiple gestation, unable to determine number of placenta and number of amniotic sacs, second trimester |
| O30893 Other specified multiple gestation, unable to determine number of placenta and number of amniotic sacs, third trimester |
| O30899 Other specified multiple gestation, unable to determine number of placenta and number of amniotic sacs, unspecified trimester |
| O3090 Multiple gestation, unspecified, unspecified trimester |
| O3091 Multiple gestation, unspecified, first trimester |
| O3092 Multiple gestation, unspecified, second trimester |
| O3093 Multiple gestation, unspecified, third trimester |
| O26872 Cervical shortening, second trimester |
| O26873 Cervical shortening, third trimester |
| O26879 Cervical shortening, unspecified trimester |
| O3430 Maternal care for cervical incompetence, unspecified trimester |
| O3431 Maternal care for cervical incompetence, first trimester |
| O3432 Maternal care for cervical incompetence, second trimester |
| O3433 Maternal care for cervical incompetence, third trimester |
| O09291 Supervision of pregnancy with other poor reproductive or obstetric history, first trimester |
| O09292 Supervision of pregnancy with other poor reproductive or obstetric history, second trimester |
| O09293 Supervision of pregnancy with other poor reproductive or obstetric history, third trimester |
| O09299 Supervision of pregnancy with other poor reproductive or obstetric history, unspecified trimester |
| O3120X0 Continuing pregnancy after intrauterine death of one fetus or more, unspecified trimester, not applicable or unspecified |
| O3120X1 Continuing pregnancy after intrauterine death of one fetus or more, unspecified trimester, fetus 1 |
| O3120X2 Continuing pregnancy after intrauterine death of one fetus or more, unspecified trimester, fetus 2 |
| O3120X3 Continuing pregnancy after intrauterine death of one fetus or more, unspecified trimester, fetus 3 |
| O3120X4 Continuing pregnancy after intrauterine death of one fetus or more, unspecified trimester, fetus 4 |
| O3120X5 Continuing pregnancy after intrauterine death of one fetus or more, unspecified trimester, fetus 5 |
| O3120X9 Continuing pregnancy after intrauterine death of one fetus or more, unspecified trimester, other fetus |
| O3121X0 Continuing pregnancy after intrauterine death of one fetus or more, first trimester, not applicable or unspecified |
| O3121X1 Continuing pregnancy after intrauterine death of one fetus or more, first trimester, fetus 1 |
| O3121X2 Continuing pregnancy after intrauterine death of one fetus or more, first trimester, fetus 2 |
| O3121X3 Continuing pregnancy after intrauterine death of one fetus or more, first trimester, fetus 3 |
| O3121X4 Continuing pregnancy after intrauterine death of one fetus or more, first trimester, fetus 4 |
| O3121X5 Continuing pregnancy after intrauterine death of one fetus or more, first trimester, fetus 5 |
| O3121X9 Continuing pregnancy after intrauterine death of one fetus or more, first trimester, other fetus |
| O3122X0 Continuing pregnancy after intrauterine death of one fetus or more, second trimester, not applicable or unspecified |
| O3122X1 Continuing pregnancy after intrauterine death of one fetus or more, second trimester, fetus 1 |
| O3122X2 Continuing pregnancy after intrauterine death of one fetus or more, second trimester, fetus 2 |
| O3122X3 Continuing pregnancy after intrauterine death of one fetus or more, second trimester, fetus 3 |
| O3122X4 Continuing pregnancy after intrauterine death of one fetus or more, second trimester, fetus 4 |
| O3122X5 Continuing pregnancy after intrauterine death of one fetus or more, second trimester, fetus 5 |
| O3122X9 Continuing pregnancy after intrauterine death of one fetus or more, second trimester, other fetus |
| O3123X0 Continuing pregnancy after intrauterine death of one fetus or more, third trimester, not applicable or unspecified |
| O3123X1 Continuing pregnancy after intrauterine death of one fetus or more, third trimester, fetus 1 |
| O3123X2 Continuing pregnancy after intrauterine death of one fetus or more, third trimester, fetus 2 |
| O3123X3 Continuing pregnancy after intrauterine death of one fetus or more, third trimester, fetus 3 |
| O3123X4 Continuing pregnancy after intrauterine death of one fetus or more, third trimester, fetus 4 |
| O3123X5 Continuing pregnancy after intrauterine death of one fetus or more, third trimester, fetus 5 |
| O3123X9 Continuing pregnancy after intrauterine death of one fetus or more, third trimester, other fetus |
| O3500X0 Maternal care for (suspected) central nervous system malformation or damage in fetus, unspecified, not applicable or unspecified |
| O3500X1 Maternal care for (suspected) central nervous system malformation or damage in fetus, unspecified, fetus 1 |
| O3500X2 Maternal care for (suspected) central nervous system malformation or damage in fetus, unspecified, fetus 2 |
| O3500X3 Maternal care for (suspected) central nervous system malformation or damage in fetus, unspecified, fetus 3 |
| O3500X4 Maternal care for (suspected) central nervous system malformation or damage in fetus, unspecified, fetus 4 |
| O3500X5 Maternal care for (suspected) central nervous system malformation or damage in fetus, unspecified, fetus 5 |
| O3500X9 Maternal care for (suspected) central nervous system malformation or damage in fetus, unspecified, other fetus |
| O3501X0 Maternal care for (suspected) central nervous system malformation or damage in fetus, agenesis of the corpus callosum, not applicable or unspecified |
| O3501X1 Maternal care for (suspected) central nervous system malformation or damage in fetus, agenesis of the corpus callosum, fetus 1 |
| O3501X2 Maternal care for (suspected) central nervous system malformation or damage in fetus, agenesis of the corpus callosum, fetus 2 |
| O3501X3 Maternal care for (suspected) central nervous system malformation or damage in fetus, agenesis of the corpus callosum, fetus 3 |
| O3501X4 Maternal care for (suspected) central nervous system malformation or damage in fetus, agenesis of the corpus callosum, fetus 4 |
| O3501X5 Maternal care for (suspected) central nervous system malformation or damage in fetus, agenesis of the corpus callosum, fetus 5 |
| O3501X9 Maternal care for (suspected) central nervous system malformation or damage in fetus, agenesis of the corpus callosum, other fetus |
| O3502X0 Maternal care for (suspected) central nervous system malformation or damage in fetus, anencephaly, not applicable or unspecified |
| O3502X1 Maternal care for (suspected) central nervous system malformation or damage in fetus, anencephaly, fetus 1 |
| O3502X2 Maternal care for (suspected) central nervous system malformation or damage in fetus, anencephaly, fetus 2 |
| O3502X3 Maternal care for (suspected) central nervous system malformation or damage in fetus, anencephaly, fetus 3 |
| O3502X4 Maternal care for (suspected) central nervous system malformation or damage in fetus, anencephaly, fetus 4 |
| O3502X5 Maternal care for (suspected) central nervous system malformation or damage in fetus, anencephaly, fetus 5 |
| O3502X9 Maternal care for (suspected) central nervous system malformation or damage in fetus, anencephaly, other fetus |
| O3503X0 Maternal care for (suspected) central nervous system malformation or damage in fetus, choroid plexus cysts, not applicable or unspecified |
| O3503X1 Maternal care for (suspected) central nervous system malformation or damage in fetus, choroid plexus cysts, fetus 1 |
| O3503X2 Maternal care for (suspected) central nervous system malformation or damage in fetus, choroid plexus cysts, fetus 2 |
| O3503X3 Maternal care for (suspected) central nervous system malformation or damage in fetus, choroid plexus cysts, fetus 3 |
| O3503X4 Maternal care for (suspected) central nervous system malformation or damage in fetus, choroid plexus cysts, fetus 4 |
| O3503X5 Maternal care for (suspected) central nervous system malformation or damage in fetus, choroid plexus cysts, fetus 5 |
| O3503X9 Maternal care for (suspected) central nervous system malformation or damage in fetus, choroid plexus cysts, other fetus |
| O3504X0 Maternal care for (suspected) central nervous system malformation or damage in fetus, encephalocele, not applicable or unspecified |
| O3504X1 Maternal care for (suspected) central nervous system malformation or damage in fetus, encephalocele, fetus 1 |
| O3504X2 Maternal care for (suspected) central nervous system malformation or damage in fetus, encephalocele, fetus 2 |
| O3504X3 Maternal care for (suspected) central nervous system malformation or damage in fetus, encephalocele, fetus 3 |
| O3504X4 Maternal care for (suspected) central nervous system malformation or damage in fetus, encephalocele, fetus 4 |
| O3504X5 Maternal care for (suspected) central nervous system malformation or damage in fetus, encephalocele, fetus 5 |
| O3504X9 Maternal care for (suspected) central nervous system malformation or damage in fetus, encephalocele, other fetus |
| O3505X0 Maternal care for (suspected) central nervous system malformation or damage in fetus, holoprosencephaly, not applicable or unspecified |
| O3505X1 Maternal care for (suspected) central nervous system malformation or damage in fetus, holoprosencephaly, fetus 1 |
| O3505X2 Maternal care for (suspected) central nervous system malformation or damage in fetus, holoprosencephaly, fetus 2 |
| O3505X3 Maternal care for (suspected) central nervous system malformation or damage in fetus, holoprosencephaly, fetus 3 |
| O3505X4 Maternal care for (suspected) central nervous system malformation or damage in fetus, holoprosencephaly, fetus 4 |
| O3505X5 Maternal care for (suspected) central nervous system malformation or damage in fetus, holoprosencephaly, fetus 5 |
| O3505X9 Maternal care for (suspected) central nervous system malformation or damage in fetus, holoprosencephaly, other fetus |
| O3506X0 Maternal care for (suspected) central nervous system malformation or damage in fetus, hydrocephaly, not applicable or unspecified |
| O3506X1 Maternal care for (suspected) central nervous system malformation or damage in fetus, hydrocephaly, fetus 1 |
| O3506X2 Maternal care for (suspected) central nervous system malformation or damage in fetus, hydrocephaly, fetus 2 |
| O3506X3 Maternal care for (suspected) central nervous system malformation or damage in fetus, hydrocephaly, fetus 3 |
| O3506X4 Maternal care for (suspected) central nervous system malformation or damage in fetus, hydrocephaly, fetus 4 |
| O3506X5 Maternal care for (suspected) central nervous system malformation or damage in fetus, hydrocephaly, fetus 5 |
| O3506X9 Maternal care for (suspected) central nervous system malformation or damage in fetus, hydrocephaly, other fetus |
| O3507X0 Maternal care for (suspected) central nervous system malformation or damage in fetus, microcephaly, not applicable or unspecified |
| O3507X1 Maternal care for (suspected) central nervous system malformation or damage in fetus, microcephaly, fetus 1 |
| O3507X2 Maternal care for (suspected) central nervous system malformation or damage in fetus, microcephaly, fetus 2 |
| O3507X3 Maternal care for (suspected) central nervous system malformation or damage in fetus, microcephaly, fetus 3 |
| O3507X4 Maternal care for (suspected) central nervous system malformation or damage in fetus, microcephaly, fetus 4 |
| O3507X5 Maternal care for (suspected) central nervous system malformation or damage in fetus, microcephaly, fetus 5 |
| O3507X9 Maternal care for (suspected) central nervous system malformation or damage in fetus, microcephaly, other fetus |
| O3508X0 Maternal care for (suspected) central nervous system malformation or damage in fetus, spina bifida, not applicable or unspecified |
| O3508X1 Maternal care for (suspected) central nervous system malformation or damage in fetus, spina bifida, fetus 1 |
| O3508X2 Maternal care for (suspected) central nervous system malformation or damage in fetus, spina bifida, fetus 2 |
| O3508X3 Maternal care for (suspected) central nervous system malformation or damage in fetus, spina bifida, fetus 3 |
| O3508X4 Maternal care for (suspected) central nervous system malformation or damage in fetus, spina bifida, fetus 4 |
| O3508X5 Maternal care for (suspected) central nervous system malformation or damage in fetus, spina bifida, fetus 5 |
| O3508X9 Maternal care for (suspected) central nervous system malformation or damage in fetus, spina bifida, other fetus |
| O3509X0 Maternal care for (suspected) other central nervous system malformation or damage in fetus, not applicable or unspecified |
| O3509X1 Maternal care for (suspected) other central nervous system malformation or damage in fetus, fetus 1 |
| O3509X2 Maternal care for (suspected) other central nervous system malformation or damage in fetus, fetus 2 |
| O3509X3 Maternal care for (suspected) other central nervous system malformation or damage in fetus, fetus 3 |
| O3509X4 Maternal care for (suspected) other central nervous system malformation or damage in fetus, fetus 4 |
| O3509X5 Maternal care for (suspected) other central nervous system malformation or damage in fetus, fetus 5 |
| O3509X9 Maternal care for (suspected) other central nervous system malformation or damage in fetus, other fetus |
| O3510X0 Maternal care for (suspected) chromosomal abnormality in fetus, unspecified, not applicable or unspecified |
| O3510X1 Maternal care for (suspected) chromosomal abnormality in fetus, unspecified, fetus 1 |
| O3510X2 Maternal care for (suspected) chromosomal abnormality in fetus, unspecified, fetus 2 |
| O3510X3 Maternal care for (suspected) chromosomal abnormality in fetus, unspecified, fetus 3 |
| O3510X4 Maternal care for (suspected) chromosomal abnormality in fetus, unspecified, fetus 4 |
| O3510X5 Maternal care for (suspected) chromosomal abnormality in fetus, unspecified, fetus 5 |
| O3510X9 Maternal care for (suspected) chromosomal abnormality in fetus, unspecified, other fetus |
| O3511X0 Maternal care for (suspected) chromosomal abnormality in fetus, Trisomy 13, not applicable or unspecified |
| O3511X1 Maternal care for (suspected) chromosomal abnormality in fetus, Trisomy 13, fetus 1 |
| O3511X2 Maternal care for (suspected) chromosomal abnormality in fetus, Trisomy 13, fetus 2 |
| O3511X3 Maternal care for (suspected) chromosomal abnormality in fetus, Trisomy 13, fetus 3 |
| O3511X4 Maternal care for (suspected) chromosomal abnormality in fetus, Trisomy 13, fetus 4 |
| O3511X5 Maternal care for (suspected) chromosomal abnormality in fetus, Trisomy 13, fetus 5 |
| O3511X9 Maternal care for (suspected) chromosomal abnormality in fetus, Trisomy 13, other fetus |
| O3512X0 Maternal care for (suspected) chromosomal abnormality in fetus, Trisomy 18, not applicable or unspecified |
| O3512X1 Maternal care for (suspected) chromosomal abnormality in fetus, Trisomy 18, fetus 1 |
| O3512X2 Maternal care for (suspected) chromosomal abnormality in fetus, Trisomy 18, fetus 2 |
| O3512X3 Maternal care for (suspected) chromosomal abnormality in fetus, Trisomy 18, fetus 3 |
| O3512X4 Maternal care for (suspected) chromosomal abnormality in fetus, Trisomy 18, fetus 4 |
| O3512X5 Maternal care for (suspected) chromosomal abnormality in fetus, Trisomy 18, fetus 5 |
| O3512X9 Maternal care for (suspected) chromosomal abnormality in fetus, Trisomy 18, other fetus |
| O3513X0 Maternal care for (suspected) chromosomal abnormality in fetus, Trisomy 21, not applicable or unspecified |
| O3513X1 Maternal care for (suspected) chromosomal abnormality in fetus, Trisomy 21, fetus 1 |
| O3513X2 Maternal care for (suspected) chromosomal abnormality in fetus, Trisomy 21, fetus 2 |
| O3513X3 Maternal care for (suspected) chromosomal abnormality in fetus, Trisomy 21, fetus 3 |
| O3513X4 Maternal care for (suspected) chromosomal abnormality in fetus, Trisomy 21, fetus 4 |
| O3513X5 Maternal care for (suspected) chromosomal abnormality in fetus, Trisomy 21, fetus 5 |
| O3513X9 Maternal care for (suspected) chromosomal abnormality in fetus, Trisomy 21, other fetus |
| O3514X0 Maternal care for (suspected) chromosomal abnormality in fetus, Turner Syndrome, not applicable or unspecified |
| O3514X1 Maternal care for (suspected) chromosomal abnormality in fetus, Turner Syndrome, fetus 1 |
| O3514X2 Maternal care for (suspected) chromosomal abnormality in fetus, Turner Syndrome, fetus 2 |
| O3514X3 Maternal care for (suspected) chromosomal abnormality in fetus, Turner Syndrome, fetus 3 |
| O3514X4 Maternal care for (suspected) chromosomal abnormality in fetus, Turner Syndrome, fetus 4 |
| O3514X5 Maternal care for (suspected) chromosomal abnormality in fetus, Turner Syndrome, fetus 5 |
| O3514X9 Maternal care for (suspected) chromosomal abnormality in fetus, Turner Syndrome, other fetus |
| O3515X0 Maternal care for (suspected) chromosomal abnormality in fetus, sex chromosome abnormality, not applicable or unspecified |
| O3515X1 Maternal care for (suspected) chromosomal abnormality in fetus, sex chromosome abnormality, fetus 1 |
| O3515X2 Maternal care for (suspected) chromosomal abnormality in fetus, sex chromosome abnormality, fetus 2 |
| O3515X3 Maternal care for (suspected) chromosomal abnormality in fetus, sex chromosome abnormality, fetus 3 |
| O3515X4 Maternal care for (suspected) chromosomal abnormality in fetus, sex chromosome abnormality, fetus 4 |
| O3515X5 Maternal care for (suspected) chromosomal abnormality in fetus, sex chromosome abnormality, fetus 5 |
| O3515X9 Maternal care for (suspected) chromosomal abnormality in fetus, sex chromosome abnormality, other fetus |
| O3519X0 Maternal care for (suspected) chromosomal abnormality in fetus, other chromosomal abnormality, not applicable or unspecified |
| O3519X1 Maternal care for (suspected) chromosomal abnormality in fetus, other chromosomal abnormality, fetus 1 |
| O3519X2 Maternal care for (suspected) chromosomal abnormality in fetus, other chromosomal abnormality, fetus 2 |
| O3519X3 Maternal care for (suspected) chromosomal abnormality in fetus, other chromosomal abnormality, fetus 3 |
| O3519X4 Maternal care for (suspected) chromosomal abnormality in fetus, other chromosomal abnormality, fetus 4 |
| O3519X5 Maternal care for (suspected) chromosomal abnormality in fetus, other chromosomal abnormality, fetus 5 |
| O3519X9 Maternal care for (suspected) chromosomal abnormality in fetus, other chromosomal abnormality, other fetus |
| O35AXX0 Maternal care for other (suspected) fetal abnormality and damage, fetal facial anomalies, not applicable or unspecified |
| O35AXX1 Maternal care for other (suspected) fetal abnormality and damage, fetal facial anomalies, fetus 1 |
| O35AXX2 Maternal care for other (suspected) fetal abnormality and damage, fetal facial anomalies, fetus 2 |
| O35AXX3 Maternal care for other (suspected) fetal abnormality and damage, fetal facial anomalies, fetus 3 |
| O35AXX4 Maternal care for other (suspected) fetal abnormality and damage, fetal facial anomalies, fetus 4 |
| O35AXX5 Maternal care for other (suspected) fetal abnormality and damage, fetal facial anomalies, fetus 5 |
| O35AXX9 Maternal care for other (suspected) fetal abnormality and damage, fetal facial anomalies, other fetus |
| O35BXX0 Maternal care for other (suspected) fetal abnormality and damage, fetal cardiac anomalies, not applicable or unspecified |
| O35BXX1 Maternal care for other (suspected) fetal abnormality and damage, fetal cardiac anomalies, fetus 1 |
| O35BXX2 Maternal care for other (suspected) fetal abnormality and damage, fetal cardiac anomalies, fetus 2 |
| O35BXX3 Maternal care for other (suspected) fetal abnormality and damage, fetal cardiac anomalies, fetus 3 |
| O35BXX4 Maternal care for other (suspected) fetal abnormality and damage, fetal cardiac anomalies, fetus 4 |
| O35BXX5 Maternal care for other (suspected) fetal abnormality and damage, fetal cardiac anomalies, fetus 5 |
| O35BXX9 Maternal care for other (suspected) fetal abnormality and damage, fetal cardiac anomalies, other fetus |
| O35CXX0 Maternal care for other (suspected) fetal abnormality and damage, fetal pulmonary anomalies, not applicable or unspecified |
| O35CXX1 Maternal care for other (suspected) fetal abnormality and damage, fetal pulmonary anomalies, fetus 1 |
| O35CXX2 Maternal care for other (suspected) fetal abnormality and damage, fetal pulmonary anomalies, fetus 2 |
| O35CXX3 Maternal care for other (suspected) fetal abnormality and damage, fetal pulmonary anomalies, fetus 3 |
| O35CXX4 Maternal care for other (suspected) fetal abnormality and damage, fetal pulmonary anomalies, fetus 4 |
| O35CXX5 Maternal care for other (suspected) fetal abnormality and damage, fetal pulmonary anomalies, fetus 5 |
| O35CXX9 Maternal care for other (suspected) fetal abnormality and damage, fetal pulmonary anomalies, other fetus |
| O35DXX0 Maternal care for other (suspected) fetal abnormality and damage, fetal gastrointestinal anomalies, not applicable or unspecified |
| O35DXX1 Maternal care for other (suspected) fetal abnormality and damage, fetal gastrointestinal anomalies, fetus 1 |
| O35DXX2 Maternal care for other (suspected) fetal abnormality and damage, fetal gastrointestinal anomalies, fetus 2 |
| O35DXX3 Maternal care for other (suspected) fetal abnormality and damage, fetal gastrointestinal anomalies, fetus 3 |
| O35DXX4 Maternal care for other (suspected) fetal abnormality and damage, fetal gastrointestinal anomalies, fetus 4 |
| O35DXX5 Maternal care for other (suspected) fetal abnormality and damage, fetal gastrointestinal anomalies, fetus 5 |
| O35DXX9 Maternal care for other (suspected) fetal abnormality and damage, fetal gastrointestinal anomalies, other fetus |
| O35EXX0 Maternal care for other (suspected) fetal abnormality and damage, fetal genitourinary anomalies, not applicable or unspecified |
| O35EXX1 Maternal care for other (suspected) fetal abnormality and damage, fetal genitourinary anomalies, fetus 1 |
| O35EXX2 Maternal care for other (suspected) fetal abnormality and damage, fetal genitourinary anomalies, fetus 2 |
| O35EXX3 Maternal care for other (suspected) fetal abnormality and damage, fetal genitourinary anomalies, fetus 3 |
| O35EXX4 Maternal care for other (suspected) fetal abnormality and damage, fetal genitourinary anomalies, fetus 4 |
| O35EXX5 Maternal care for other (suspected) fetal abnormality and damage, fetal genitourinary anomalies, fetus 5 |
| O35EXX9 Maternal care for other (suspected) fetal abnormality and damage, fetal genitourinary anomalies, other fetus |
| O35FXX0 Maternal care for other (suspected) fetal abnormality and damage, fetal musculoskeletal anomalies of trunk, not applicable or unspecified |
| O35FXX1 Maternal care for other (suspected) fetal abnormality and damage, fetal musculoskeletal anomalies of trunk, fetus 1 |
| O35FXX2 Maternal care for other (suspected) fetal abnormality and damage, fetal musculoskeletal anomalies of trunk, fetus 2 |
| O35FXX3 Maternal care for other (suspected) fetal abnormality and damage, fetal musculoskeletal anomalies of trunk, fetus 3 |
| O35FXX4 Maternal care for other (suspected) fetal abnormality and damage, fetal musculoskeletal anomalies of trunk, fetus 4 |
| O35FXX5 Maternal care for other (suspected) fetal abnormality and damage, fetal musculoskeletal anomalies of trunk, fetus 5 |
| O35FXX9 Maternal care for other (suspected) fetal abnormality and damage, fetal musculoskeletal anomalies of trunk, other fetus |
| O35GXX0 Maternal care for other (suspected) fetal abnormality and damage, fetal upper extremities anomalies, not applicable or unspecified |
| O35GXX1 Maternal care for other (suspected) fetal abnormality and damage, fetal upper extremities anomalies, fetus 1 |
| O35GXX2 Maternal care for other (suspected) fetal abnormality and damage, fetal upper extremities anomalies, fetus 2 |
| O35GXX3 Maternal care for other (suspected) fetal abnormality and damage, fetal upper extremities anomalies, fetus 3 |
| O35GXX4 Maternal care for other (suspected) fetal abnormality and damage, fetal upper extremities anomalies, fetus 4 |
| O35GXX5 Maternal care for other (suspected) fetal abnormality and damage, fetal upper extremities anomalies, fetus 5 |
| O35GXX9 Maternal care for other (suspected) fetal abnormality and damage, fetal upper extremities anomalies, other fetus |
| O35HXX0 Maternal care for other (suspected) fetal abnormality and damage, fetal lower extremities anomalies, not applicable or unspecified |
| O35HXX1 Maternal care for other (suspected) fetal abnormality and damage, fetal lower extremities anomalies, fetus 1 |
| O35HXX2 Maternal care for other (suspected) fetal abnormality and damage, fetal lower extremities anomalies, fetus 2 |
| O35HXX3 Maternal care for other (suspected) fetal abnormality and damage, fetal lower extremities anomalies, fetus 3 |
| O35HXX4 Maternal care for other (suspected) fetal abnormality and damage, fetal lower extremities anomalies, fetus 4 |
| O35HXX5 Maternal care for other (suspected) fetal abnormality and damage, fetal lower extremities anomalies, fetus 5 |
| O35HXX9 Maternal care for other (suspected) fetal abnormality and damage, fetal lower extremities anomalies, other fetus |
| O352XX0 Maternal care for (suspected) hereditary disease in fetus, not applicable or unspecified |
| O352XX1 Maternal care for (suspected) hereditary disease in fetus, fetus 1 |
| O352XX2 Maternal care for (suspected) hereditary disease in fetus, fetus 2 |
| O352XX3 Maternal care for (suspected) hereditary disease in fetus, fetus 3 |
| O352XX4 Maternal care for (suspected) hereditary disease in fetus, fetus 4 |
| O352XX5 Maternal care for (suspected) hereditary disease in fetus, fetus 5 |
| O352XX9 Maternal care for (suspected) hereditary disease in fetus, other fetus |
| O353XX0 Maternal care for (suspected) damage to fetus from viral disease in mother, not applicable or unspecified |
| O353XX1 Maternal care for (suspected) damage to fetus from viral disease in mother, fetus 1 |
| O353XX2 Maternal care for (suspected) damage to fetus from viral disease in mother, fetus 2 |
| O353XX3 Maternal care for (suspected) damage to fetus from viral disease in mother, fetus 3 |
| O353XX4 Maternal care for (suspected) damage to fetus from viral disease in mother, fetus 4 |
| O353XX5 Maternal care for (suspected) damage to fetus from viral disease in mother, fetus 5 |
| O353XX9 Maternal care for (suspected) damage to fetus from viral disease in mother, other fetus |
| O354XX0 Maternal care for (suspected) damage to fetus from alcohol, not applicable or unspecified |
| O354XX1 Maternal care for (suspected) damage to fetus from alcohol, fetus 1 |
| O354XX2 Maternal care for (suspected) damage to fetus from alcohol, fetus 2 |
| O354XX3 Maternal care for (suspected) damage to fetus from alcohol, fetus 3 |
| O354XX4 Maternal care for (suspected) damage to fetus from alcohol, fetus 4 |
| O354XX5 Maternal care for (suspected) damage to fetus from alcohol, fetus 5 |
| O354XX9 Maternal care for (suspected) damage to fetus from alcohol, other fetus |
| O355XX0 Maternal care for (suspected) damage to fetus by drugs, not applicable or unspecified |
| O355XX1 Maternal care for (suspected) damage to fetus by drugs, fetus 1 |
| O355XX2 Maternal care for (suspected) damage to fetus by drugs, fetus 2 |
| O355XX3 Maternal care for (suspected) damage to fetus by drugs, fetus 3 |
| O355XX4 Maternal care for (suspected) damage to fetus by drugs, fetus 4 |
| O355XX5 Maternal care for (suspected) damage to fetus by drugs, fetus 5 |
| O355XX9 Maternal care for (suspected) damage to fetus by drugs, other fetus |
| O356XX0 Maternal care for (suspected) damage to fetus by radiation, not applicable or unspecified |
| O356XX1 Maternal care for (suspected) damage to fetus by radiation, fetus 1 |
| O356XX2 Maternal care for (suspected) damage to fetus by radiation, fetus 2 |
| O356XX3 Maternal care for (suspected) damage to fetus by radiation, fetus 3 |
| O356XX4 Maternal care for (suspected) damage to fetus by radiation, fetus 4 |
| O356XX5 Maternal care for (suspected) damage to fetus by radiation, fetus 5 |
| O356XX9 Maternal care for (suspected) damage to fetus by radiation, other fetus |
| O357XX0 Maternal care for (suspected) damage to fetus by other medical procedures, not applicable or unspecified |
| O357XX1 Maternal care for (suspected) damage to fetus by other medical procedures, fetus 1 |
| O357XX2 Maternal care for (suspected) damage to fetus by other medical procedures, fetus 2 |
| O357XX3 Maternal care for (suspected) damage to fetus by other medical procedures, fetus 3 |
| O357XX4 Maternal care for (suspected) damage to fetus by other medical procedures, fetus 4 |
| O357XX5 Maternal care for (suspected) damage to fetus by other medical procedures, fetus 5 |
| O357XX9 Maternal care for (suspected) damage to fetus by other medical procedures, other fetus |
| O358XX0 Maternal care for other (suspected) fetal abnormality and damage, not applicable or unspecified |
| O358XX1 Maternal care for other (suspected) fetal abnormality and damage, fetus 1 |
| O358XX2 Maternal care for other (suspected) fetal abnormality and damage, fetus 2 |
| O358XX3 Maternal care for other (suspected) fetal abnormality and damage, fetus 3 |
| O358XX4 Maternal care for other (suspected) fetal abnormality and damage, fetus 4 |
| O358XX5 Maternal care for other (suspected) fetal abnormality and damage, fetus 5 |
| O358XX9 Maternal care for other (suspected) fetal abnormality and damage, other fetus |
| O359XX0 Maternal care for (suspected) fetal abnormality and damage, unspecified, not applicable or unspecified |
| O359XX1 Maternal care for (suspected) fetal abnormality and damage, unspecified, fetus 1 |
| O359XX2 Maternal care for (suspected) fetal abnormality and damage, unspecified, fetus 2 |
| O359XX3 Maternal care for (suspected) fetal abnormality and damage, unspecified, fetus 3 |
| O359XX4 Maternal care for (suspected) fetal abnormality and damage, unspecified, fetus 4 |
| O359XX5 Maternal care for (suspected) fetal abnormality and damage, unspecified, fetus 5 |
| O359XX9 Maternal care for (suspected) fetal abnormality and damage, unspecified, other fetus |
| O360110 Maternal care for anti-D [Rh] antibodies, first trimester, not applicable or unspecified |
| O360111 Maternal care for anti-D [Rh] antibodies, first trimester, fetus 1 |
| O360112 Maternal care for anti-D [Rh] antibodies, first trimester, fetus 2 |
| O360113 Maternal care for anti-D [Rh] antibodies, first trimester, fetus 3 |
| O360114 Maternal care for anti-D [Rh] antibodies, first trimester, fetus 4 |
| O360115 Maternal care for anti-D [Rh] antibodies, first trimester, fetus 5 |
| O360119 Maternal care for anti-D [Rh] antibodies, first trimester, other fetus |
| O360120 Maternal care for anti-D [Rh] antibodies, second trimester, not applicable or unspecified |
| O360121 Maternal care for anti-D [Rh] antibodies, second trimester, fetus 1 |
| O360122 Maternal care for anti-D [Rh] antibodies, second trimester, fetus 2 |
| O360123 Maternal care for anti-D [Rh] antibodies, second trimester, fetus 3 |
| O360124 Maternal care for anti-D [Rh] antibodies, second trimester, fetus 4 |
| O360125 Maternal care for anti-D [Rh] antibodies, second trimester, fetus 5 |
| O360129 Maternal care for anti-D [Rh] antibodies, second trimester, other fetus |
| O360130 Maternal care for anti-D [Rh] antibodies, third trimester, not applicable or unspecified |
| O360131 Maternal care for anti-D [Rh] antibodies, third trimester, fetus 1 |
| O360132 Maternal care for anti-D [Rh] antibodies, third trimester, fetus 2 |
| O360133 Maternal care for anti-D [Rh] antibodies, third trimester, fetus 3 |
| O360134 Maternal care for anti-D [Rh] antibodies, third trimester, fetus 4 |
| O360135 Maternal care for anti-D [Rh] antibodies, third trimester, fetus 5 |
| O360139 Maternal care for anti-D [Rh] antibodies, third trimester, other fetus |
| O360190 Maternal care for anti-D [Rh] antibodies, unspecified trimester, not applicable or unspecified |
| O360191 Maternal care for anti-D [Rh] antibodies, unspecified trimester, fetus 1 |
| O360192 Maternal care for anti-D [Rh] antibodies, unspecified trimester, fetus 2 |
| O360193 Maternal care for anti-D [Rh] antibodies, unspecified trimester, fetus 3 |
| O360194 Maternal care for anti-D [Rh] antibodies, unspecified trimester, fetus 4 |
| O360195 Maternal care for anti-D [Rh] antibodies, unspecified trimester, fetus 5 |
| O360199 Maternal care for anti-D [Rh] antibodies, unspecified trimester, other fetus |
| O360910 Maternal care for other rhesus isoimmunization, first trimester, not applicable or unspecified |
| O360911 Maternal care for other rhesus isoimmunization, first trimester, fetus 1 |
| O360912 Maternal care for other rhesus isoimmunization, first trimester, fetus 2 |
| O360913 Maternal care for other rhesus isoimmunization, first trimester, fetus 3 |
| O360914 Maternal care for other rhesus isoimmunization, first trimester, fetus 4 |
| O360915 Maternal care for other rhesus isoimmunization, first trimester, fetus 5 |
| O360919 Maternal care for other rhesus isoimmunization, first trimester, other fetus |
| O360920 Maternal care for other rhesus isoimmunization, second trimester, not applicable or unspecified |
| O360921 Maternal care for other rhesus isoimmunization, second trimester, fetus 1 |
| O360922 Maternal care for other rhesus isoimmunization, second trimester, fetus 2 |
| O360923 Maternal care for other rhesus isoimmunization, second trimester, fetus 3 |
| O360924 Maternal care for other rhesus isoimmunization, second trimester, fetus 4 |
| O360925 Maternal care for other rhesus isoimmunization, second trimester, fetus 5 |
| O360929 Maternal care for other rhesus isoimmunization, second trimester, other fetus |
| O360930 Maternal care for other rhesus isoimmunization, third trimester, not applicable or unspecified |
| O360931 Maternal care for other rhesus isoimmunization, third trimester, fetus 1 |
| O360932 Maternal care for other rhesus isoimmunization, third trimester, fetus 2 |
| O360933 Maternal care for other rhesus isoimmunization, third trimester, fetus 3 |
| O360934 Maternal care for other rhesus isoimmunization, third trimester, fetus 4 |
| O360935 Maternal care for other rhesus isoimmunization, third trimester, fetus 5 |
| O360939 Maternal care for other rhesus isoimmunization, third trimester, other fetus |
| O360990 Maternal care for other rhesus isoimmunization, unspecified trimester, not applicable or unspecified |
| O360991 Maternal care for other rhesus isoimmunization, unspecified trimester, fetus 1 |
| O360992 Maternal care for other rhesus isoimmunization, unspecified trimester, fetus 2 |
| O360993 Maternal care for other rhesus isoimmunization, unspecified trimester, fetus 3 |
| O360994 Maternal care for other rhesus isoimmunization, unspecified trimester, fetus 4 |
| O360995 Maternal care for other rhesus isoimmunization, unspecified trimester, fetus 5 |
| O360999 Maternal care for other rhesus isoimmunization, unspecified trimester, other fetus |
| O361110 Maternal care for Anti-A sensitization, first trimester, not applicable or unspecified |
| O361111 Maternal care for Anti-A sensitization, first trimester, fetus 1 |
| O361112 Maternal care for Anti-A sensitization, first trimester, fetus 2 |
| O361113 Maternal care for Anti-A sensitization, first trimester, fetus 3 |
| O361114 Maternal care for Anti-A sensitization, first trimester, fetus 4 |
| O361115 Maternal care for Anti-A sensitization, first trimester, fetus 5 |
| O361119 Maternal care for Anti-A sensitization, first trimester, other fetus |
| O361120 Maternal care for Anti-A sensitization, second trimester, not applicable or unspecified |
| O361121 Maternal care for Anti-A sensitization, second trimester, fetus 1 |
| O361122 Maternal care for Anti-A sensitization, second trimester, fetus 2 |
| O361123 Maternal care for Anti-A sensitization, second trimester, fetus 3 |
| O361124 Maternal care for Anti-A sensitization, second trimester, fetus 4 |
| O361125 Maternal care for Anti-A sensitization, second trimester, fetus 5 |
| O361129 Maternal care for Anti-A sensitization, second trimester, other fetus |
| O361130 Maternal care for Anti-A sensitization, third trimester, not applicable or unspecified |
| O361131 Maternal care for Anti-A sensitization, third trimester, fetus 1 |
| O361132 Maternal care for Anti-A sensitization, third trimester, fetus 2 |
| O361133 Maternal care for Anti-A sensitization, third trimester, fetus 3 |
| O361134 Maternal care for Anti-A sensitization, third trimester, fetus 4 |
| O361135 Maternal care for Anti-A sensitization, third trimester, fetus 5 |
| O361139 Maternal care for Anti-A sensitization, third trimester, other fetus |
| O361190 Maternal care for Anti-A sensitization, unspecified trimester, not applicable or unspecified |
| O361191 Maternal care for Anti-A sensitization, unspecified trimester, fetus 1 |
| O361192 Maternal care for Anti-A sensitization, unspecified trimester, fetus 2 |
| O361193 Maternal care for Anti-A sensitization, unspecified trimester, fetus 3 |
| O361194 Maternal care for Anti-A sensitization, unspecified trimester, fetus 4 |
| O361195 Maternal care for Anti-A sensitization, unspecified trimester, fetus 5 |
| O361199 Maternal care for Anti-A sensitization, unspecified trimester, other fetus |
| O361910 Maternal care for other isoimmunization, first trimester, not applicable or unspecified |
| O361911 Maternal care for other isoimmunization, first trimester, fetus 1 |
| O361912 Maternal care for other isoimmunization, first trimester, fetus 2 |
| O361913 Maternal care for other isoimmunization, first trimester, fetus 3 |
| O361914 Maternal care for other isoimmunization, first trimester, fetus 4 |
| O361915 Maternal care for other isoimmunization, first trimester, fetus 5 |
| O361919 Maternal care for other isoimmunization, first trimester, other fetus |
| O361920 Maternal care for other isoimmunization, second trimester, not applicable or unspecified |
| O361921 Maternal care for other isoimmunization, second trimester, fetus 1 |
| O361922 Maternal care for other isoimmunization, second trimester, fetus 2 |
| O361923 Maternal care for other isoimmunization, second trimester, fetus 3 |
| O361924 Maternal care for other isoimmunization, second trimester, fetus 4 |
| O361925 Maternal care for other isoimmunization, second trimester, fetus 5 |
| O361929 Maternal care for other isoimmunization, second trimester, other fetus |
| O361930 Maternal care for other isoimmunization, third trimester, not applicable or unspecified |
| O361931 Maternal care for other isoimmunization, third trimester, fetus 1 |
| O361932 Maternal care for other isoimmunization, third trimester, fetus 2 |
| O361933 Maternal care for other isoimmunization, third trimester, fetus 3 |
| O361934 Maternal care for other isoimmunization, third trimester, fetus 4 |
| O361935 Maternal care for other isoimmunization, third trimester, fetus 5 |
| O361939 Maternal care for other isoimmunization, third trimester, other fetus |
| O361990 Maternal care for other isoimmunization, unspecified trimester, not applicable or unspecified |
| O361991 Maternal care for other isoimmunization, unspecified trimester, fetus 1 |
| O361992 Maternal care for other isoimmunization, unspecified trimester, fetus 2 |
| O361993 Maternal care for other isoimmunization, unspecified trimester, fetus 3 |
| O361994 Maternal care for other isoimmunization, unspecified trimester, fetus 4 |
| O361995 Maternal care for other isoimmunization, unspecified trimester, fetus 5 |
| O361999 Maternal care for other isoimmunization, unspecified trimester, other fetus |
| O3620X0 Maternal care for hydrops fetalis, unspecified trimester, not applicable or unspecified |
| O3620X1 Maternal care for hydrops fetalis, unspecified trimester, fetus 1 |
| O3620X2 Maternal care for hydrops fetalis, unspecified trimester, fetus 2 |
| O3620X3 Maternal care for hydrops fetalis, unspecified trimester, fetus 3 |
| O3620X4 Maternal care for hydrops fetalis, unspecified trimester, fetus 4 |
| O3620X5 Maternal care for hydrops fetalis, unspecified trimester, fetus 5 |
| O3620X9 Maternal care for hydrops fetalis, unspecified trimester, other fetus |
| O3621X0 Maternal care for hydrops fetalis, first trimester, not applicable or unspecified |
| O3621X1 Maternal care for hydrops fetalis, first trimester, fetus 1 |
| O3621X2 Maternal care for hydrops fetalis, first trimester, fetus 2 |
| O3621X3 Maternal care for hydrops fetalis, first trimester, fetus 3 |
| O3621X4 Maternal care for hydrops fetalis, first trimester, fetus 4 |
| O3621X5 Maternal care for hydrops fetalis, first trimester, fetus 5 |
| O3621X9 Maternal care for hydrops fetalis, first trimester, other fetus |
| O3622X0 Maternal care for hydrops fetalis, second trimester, not applicable or unspecified |
| O3622X1 Maternal care for hydrops fetalis, second trimester, fetus 1 |
| O3622X2 Maternal care for hydrops fetalis, second trimester, fetus 2 |
| O3622X3 Maternal care for hydrops fetalis, second trimester, fetus 3 |
| O3622X4 Maternal care for hydrops fetalis, second trimester, fetus 4 |
| O3622X5 Maternal care for hydrops fetalis, second trimester, fetus 5 |
| O3622X9 Maternal care for hydrops fetalis, second trimester, other fetus |
| O3623X0 Maternal care for hydrops fetalis, third trimester, not applicable or unspecified |
| O3623X1 Maternal care for hydrops fetalis, third trimester, fetus 1 |
| O3623X2 Maternal care for hydrops fetalis, third trimester, fetus 2 |
| O3623X3 Maternal care for hydrops fetalis, third trimester, fetus 3 |
| O3623X4 Maternal care for hydrops fetalis, third trimester, fetus 4 |
| O3623X5 Maternal care for hydrops fetalis, third trimester, fetus 5 |
| O3623X9 Maternal care for hydrops fetalis, third trimester, other fetus |
| O364XX0 Maternal care for intrauterine death, not applicable or unspecified |
| O364XX1 Maternal care for intrauterine death, fetus 1 |
| O364XX2 Maternal care for intrauterine death, fetus 2 |
| O364XX3 Maternal care for intrauterine death, fetus 3 |
| O364XX4 Maternal care for intrauterine death, fetus 4 |
| O364XX5 Maternal care for intrauterine death, fetus 5 |
| O364XX9 Maternal care for intrauterine death, other fetus |
| O365110 Maternal care for known or suspected placental insufficiency, first trimester, not applicable or unspecified |
| O365111 Maternal care for known or suspected placental insufficiency, first trimester, fetus 1 |
| O365112 Maternal care for known or suspected placental insufficiency, first trimester, fetus 2 |
| O365113 Maternal care for known or suspected placental insufficiency, first trimester, fetus 3 |
| O365114 Maternal care for known or suspected placental insufficiency, first trimester, fetus 4 |
| O365115 Maternal care for known or suspected placental insufficiency, first trimester, fetus 5 |
| O365119 Maternal care for known or suspected placental insufficiency, first trimester, other fetus |
| O365120 Maternal care for known or suspected placental insufficiency, second trimester, not applicable or unspecified |
| O365121 Maternal care for known or suspected placental insufficiency, second trimester, fetus 1 |
| O365122 Maternal care for known or suspected placental insufficiency, second trimester, fetus 2 |
| O365123 Maternal care for known or suspected placental insufficiency, second trimester, fetus 3 |
| O365124 Maternal care for known or suspected placental insufficiency, second trimester, fetus 4 |
| O365125 Maternal care for known or suspected placental insufficiency, second trimester, fetus 5 |
| O365129 Maternal care for known or suspected placental insufficiency, second trimester, other fetus |
| O365130 Maternal care for known or suspected placental insufficiency, third trimester, not applicable or unspecified |
| O365131 Maternal care for known or suspected placental insufficiency, third trimester, fetus 1 |
| O365132 Maternal care for known or suspected placental insufficiency, third trimester, fetus 2 |
| O365133 Maternal care for known or suspected placental insufficiency, third trimester, fetus 3 |
| O365134 Maternal care for known or suspected placental insufficiency, third trimester, fetus 4 |
| O365135 Maternal care for known or suspected placental insufficiency, third trimester, fetus 5 |
| O365139 Maternal care for known or suspected placental insufficiency, third trimester, other fetus |
| O365190 Maternal care for known or suspected placental insufficiency, unspecified trimester, not applicable or unspecified |
| O365191 Maternal care for known or suspected placental insufficiency, unspecified trimester, fetus 1 |
| O365192 Maternal care for known or suspected placental insufficiency, unspecified trimester, fetus 2 |
| O365193 Maternal care for known or suspected placental insufficiency, unspecified trimester, fetus 3 |
| O365194 Maternal care for known or suspected placental insufficiency, unspecified trimester, fetus 4 |
| O365195 Maternal care for known or suspected placental insufficiency, unspecified trimester, fetus 5 |
| O365199 Maternal care for known or suspected placental insufficiency, unspecified trimester, other fetus |
| O365910 Maternal care for other known or suspected poor fetal growth, first trimester, not applicable or unspecified |
| O365911 Maternal care for other known or suspected poor fetal growth, first trimester, fetus 1 |
| O365912 Maternal care for other known or suspected poor fetal growth, first trimester, fetus 2 |
| O365913 Maternal care for other known or suspected poor fetal growth, first trimester, fetus 3 |
| O365914 Maternal care for other known or suspected poor fetal growth, first trimester, fetus 4 |
| O365915 Maternal care for other known or suspected poor fetal growth, first trimester, fetus 5 |
| O365919 Maternal care for other known or suspected poor fetal growth, first trimester, other fetus |
| O365920 Maternal care for other known or suspected poor fetal growth, second trimester, not applicable or unspecified |
| O365921 Maternal care for other known or suspected poor fetal growth, second trimester, fetus 1 |
| O365922 Maternal care for other known or suspected poor fetal growth, second trimester, fetus 2 |
| O365923 Maternal care for other known or suspected poor fetal growth, second trimester, fetus 3 |
| O365924 Maternal care for other known or suspected poor fetal growth, second trimester, fetus 4 |
| O365925 Maternal care for other known or suspected poor fetal growth, second trimester, fetus 5 |
| O365929 Maternal care for other known or suspected poor fetal growth, second trimester, other fetus |
| O365930 Maternal care for other known or suspected poor fetal growth, third trimester, not applicable or unspecified |
| O365931 Maternal care for other known or suspected poor fetal growth, third trimester, fetus 1 |
| O365932 Maternal care for other known or suspected poor fetal growth, third trimester, fetus 2 |
| O365933 Maternal care for other known or suspected poor fetal growth, third trimester, fetus 3 |
| O365934 Maternal care for other known or suspected poor fetal growth, third trimester, fetus 4 |
| O365935 Maternal care for other known or suspected poor fetal growth, third trimester, fetus 5 |
| O365939 Maternal care for other known or suspected poor fetal growth, third trimester, other fetus |
| O365990 Maternal care for other known or suspected poor fetal growth, unspecified trimester, not applicable or unspecified |
| O365991 Maternal care for other known or suspected poor fetal growth, unspecified trimester, fetus 1 |
| O365992 Maternal care for other known or suspected poor fetal growth, unspecified trimester, fetus 2 |
| O365993 Maternal care for other known or suspected poor fetal growth, unspecified trimester, fetus 3 |
| O365994 Maternal care for other known or suspected poor fetal growth, unspecified trimester, fetus 4 |
| O365995 Maternal care for other known or suspected poor fetal growth, unspecified trimester, fetus 5 |
| O365999 Maternal care for other known or suspected poor fetal growth, unspecified trimester, other fetus |
| O368210 Fetal anemia and thrombocytopenia, first trimester, not applicable or unspecified |
| O368211 Fetal anemia and thrombocytopenia, first trimester, fetus 1 |
| O368212 Fetal anemia and thrombocytopenia, first trimester, fetus 2 |
| O368213 Fetal anemia and thrombocytopenia, first trimester, fetus 3 |
| O368214 Fetal anemia and thrombocytopenia, first trimester, fetus 4 |
| O368215 Fetal anemia and thrombocytopenia, first trimester, fetus 5 |
| O368219 Fetal anemia and thrombocytopenia, first trimester, other fetus |
| O368220 Fetal anemia and thrombocytopenia, second trimester, not applicable or unspecified |
| O368221 Fetal anemia and thrombocytopenia, second trimester, fetus 1 |
| O368222 Fetal anemia and thrombocytopenia, second trimester, fetus 2 |
| O368223 Fetal anemia and thrombocytopenia, second trimester, fetus 3 |
| O368224 Fetal anemia and thrombocytopenia, second trimester, fetus 4 |
| O368225 Fetal anemia and thrombocytopenia, second trimester, fetus 5 |
| O368229 Fetal anemia and thrombocytopenia, second trimester, other fetus |
| O368230 Fetal anemia and thrombocytopenia, third trimester, not applicable or unspecified |
| O368231 Fetal anemia and thrombocytopenia, third trimester, fetus 1 |
| O368232 Fetal anemia and thrombocytopenia, third trimester, fetus 2 |
| O368233 Fetal anemia and thrombocytopenia, third trimester, fetus 3 |
| O368234 Fetal anemia and thrombocytopenia, third trimester, fetus 4 |
| O368235 Fetal anemia and thrombocytopenia, third trimester, fetus 5 |
| O368239 Fetal anemia and thrombocytopenia, third trimester, other fetus |
| O368290 Fetal anemia and thrombocytopenia, unspecified trimester, not applicable or unspecified |
| O368291 Fetal anemia and thrombocytopenia, unspecified trimester, fetus 1 |
| O368292 Fetal anemia and thrombocytopenia, unspecified trimester, fetus 2 |
| O368293 Fetal anemia and thrombocytopenia, unspecified trimester, fetus 3 |
| O368294 Fetal anemia and thrombocytopenia, unspecified trimester, fetus 4 |
| O368295 Fetal anemia and thrombocytopenia, unspecified trimester, fetus 5 |
| O368299 Fetal anemia and thrombocytopenia, unspecified trimester, other fetus |
| O368910 Maternal care for other specified fetal problems, first trimester, not applicable or unspecified |
| O368911 Maternal care for other specified fetal problems, first trimester, fetus 1 |
| O368912 Maternal care for other specified fetal problems, first trimester, fetus 2 |
| O368913 Maternal care for other specified fetal problems, first trimester, fetus 3 |
| O368914 Maternal care for other specified fetal problems, first trimester, fetus 4 |
| O368915 Maternal care for other specified fetal problems, first trimester, fetus 5 |
| O368919 Maternal care for other specified fetal problems, first trimester, other fetus |
| O368920 Maternal care for other specified fetal problems, second trimester, not applicable or unspecified |
| O368921 Maternal care for other specified fetal problems, second trimester, fetus 1 |
| O368922 Maternal care for other specified fetal problems, second trimester, fetus 2 |
| O368923 Maternal care for other specified fetal problems, second trimester, fetus 3 |
| O368924 Maternal care for other specified fetal problems, second trimester, fetus 4 |
| O368925 Maternal care for other specified fetal problems, second trimester, fetus 5 |
| O368929 Maternal care for other specified fetal problems, second trimester, other fetus |
| O368930 Maternal care for other specified fetal problems, third trimester, not applicable or unspecified |
| O368931 Maternal care for other specified fetal problems, third trimester, fetus 1 |
| O368932 Maternal care for other specified fetal problems, third trimester, fetus 2 |
| O368933 Maternal care for other specified fetal problems, third trimester, fetus 3 |
| O368934 Maternal care for other specified fetal problems, third trimester, fetus 4 |
| O368935 Maternal care for other specified fetal problems, third trimester, fetus 5 |
| O368939 Maternal care for other specified fetal problems, third trimester, other fetus |
| O368990 Maternal care for other specified fetal problems, unspecified trimester, not applicable or unspecified |
| O368991 Maternal care for other specified fetal problems, unspecified trimester, fetus 1 |
| O368992 Maternal care for other specified fetal problems, unspecified trimester, fetus 2 |
| O368993 Maternal care for other specified fetal problems, unspecified trimester, fetus 3 |
| O368994 Maternal care for other specified fetal problems, unspecified trimester, fetus 4 |
| O368995 Maternal care for other specified fetal problems, unspecified trimester, fetus 5 |
| O368999 Maternal care for other specified fetal problems, unspecified trimester, other fetus |
| O3690X0 Maternal care for fetal problem, unspecified, unspecified trimester, not applicable or unspecified |
| O3690X1 Maternal care for fetal problem, unspecified, unspecified trimester, fetus 1 |
| O3690X2 Maternal care for fetal problem, unspecified, unspecified trimester, fetus 2 |
| O3690X3 Maternal care for fetal problem, unspecified, unspecified trimester, fetus 3 |
| O3690X4 Maternal care for fetal problem, unspecified, unspecified trimester, fetus 4 |
| O3690X5 Maternal care for fetal problem, unspecified, unspecified trimester, fetus 5 |
| O3690X9 Maternal care for fetal problem, unspecified, unspecified trimester, other fetus |
| O3691X0 Maternal care for fetal problem, unspecified, first trimester, not applicable or unspecified |
| O3691X1 Maternal care for fetal problem, unspecified, first trimester, fetus 1 |
| O3691X2 Maternal care for fetal problem, unspecified, first trimester, fetus 2 |
| O3691X3 Maternal care for fetal problem, unspecified, first trimester, fetus 3 |
| O3691X4 Maternal care for fetal problem, unspecified, first trimester, fetus 4 |
| O3691X5 Maternal care for fetal problem, unspecified, first trimester, fetus 5 |
| O3691X9 Maternal care for fetal problem, unspecified, first trimester, other fetus |
| O3692X0 Maternal care for fetal problem, unspecified, second trimester, not applicable or unspecified |
| O3692X1 Maternal care for fetal problem, unspecified, second trimester, fetus 1 |
| O3692X2 Maternal care for fetal problem, unspecified, second trimester, fetus 2 |
| O3692X3 Maternal care for fetal problem, unspecified, second trimester, fetus 3 |
| O3692X4 Maternal care for fetal problem, unspecified, second trimester, fetus 4 |
| O3692X5 Maternal care for fetal problem, unspecified, second trimester, fetus 5 |
| O3692X9 Maternal care for fetal problem, unspecified, second trimester, other fetus |
| O3693X0 Maternal care for fetal problem, unspecified, third trimester, not applicable or unspecified |
| O3693X1 Maternal care for fetal problem, unspecified, third trimester, fetus 1 |
| O3693X2 Maternal care for fetal problem, unspecified, third trimester, fetus 2 |
| O3693X3 Maternal care for fetal problem, unspecified, third trimester, fetus 3 |
| O3693X4 Maternal care for fetal problem, unspecified, third trimester, fetus 4 |
| O3693X5 Maternal care for fetal problem, unspecified, third trimester, fetus 5 |
| O3693X9 Maternal care for fetal problem, unspecified, third trimester, other fetus |
